# Multi-objective optimizing spring placement and stiffness in slider-crank mechanisms for enhanced dynamic parameters

**DOI:** 10.1371/journal.pone.0331341

**Published:** 2025-09-08

**Authors:** Hoang Minh Dang, Van Binh Phung, Van Thanh Tien Nguyen

**Affiliations:** 1 Department of design fundamentals, Faculty of Mechanical Engineering, Industrial University of Ho Chi Minh City, Ho Chi Minh City, Vietnam; 2 Faculty of Aerospace Engineering, Le Quy Don Technical University, Hanoi, Vietnam; National Chung Cheng University, Taiwan & Australian Center for Sustainable Development Research and Innovation (ACSDRI), AUSTRALIA

## Abstract

The slider-crank mechanism (SCM) is fundamental to various mechanical systems. However, optimizing its dynamic performance remains a pressing challenge due to excessive torque, joint reactions, and energy consumption. This study introduces two key innovations to address these challenges: (1) the integration of springs into SCM to optimize dynamic performance and (2) a novel hybrid optimization approach combining the Conjugate Direction with Orthogonal Shift (CDOS) method and Parameter Space Investigation (PSI). The mathematical model evaluates the effects of spring placement and stiffness on critical performance parameters such as energy efficiency, torque demands, and joint forces. The hybrid CDOS-PSI approach systematically identifies optimal design configurations to balance these performance objectives. The methodology’s efficacy is validated through a case study on a wood splitter, a commonly used agricultural and industrial machine. Experimental tests were carried out to measure splitting forces for different wood types, enabling accurate model calibration. Results demonstrate that the spring-integrated SCM reduces dynamic loads significantly compared to conventional designs. Comparative numerical analysis confirms the proposed model’s accuracy, with less than 5% deviations. This research offers innovative contributions to SCM design by combining spring-based dynamic enhancement with a novel hybrid optimization framework for improved efficiency and durability in practical applications.

## 1. Introduction

The slider-crank mechanism (SCM) is fundamental in numerous mechanical systems, from industrial machinery to agricultural equipment. Despite its widespread use, SCMs are inherently challenged by cyclic inertial forces and moments of inertia, contributing to system vibrations, excessive energy consumption, and reduced fatigue strength. Addressing these challenges remains a key research focus to enhance the efficiency and durability of SCM-based systems. A preliminary version of this study was presented at the 2023 International Conference on Sustainable Energy Technologies (ICSET2023) [1]. This article builds upon that work with significant improvements and updated findings that provide deeper insights into SCM optimization.

Extensive studies have been conducted to minimize the impact of inertial forces in SCMs. Arakelian and Smith [2] provided a comprehensive historical review on balancing shaking forces and moments in mechanical systems. They highlighted that a balanced design can significantly reduce wear and improve system reliability. Building on this, Arakelian and Briot [3] demonstrated the feasibility of simultaneously balancing inertial forces and compensating torque in SCMs. These foundational studies paved the way for exploring various balancing techniques, including counterweights and spring-based systems. However, the limitations of counterweights, such as increased size and system bulkiness, often make them unsuitable for compact designs [3,4].

Recent advancements, such as the work of Dang et al. [5], introduced a generalized mathematical model for SCM synthesis based on multi-objective optimization principles. Similarly, Nga et al. [6] applied dynamic analysis and optimization techniques to specific applications, highlighting the importance of minimizing energy consumption and torque requirements in SCM-based systems. However, these studies did not comprehensively address the interplay between spring placement and stiffness, nor did they evaluate their combined impact on dynamic performance metrics.

While various optimization approaches have been applied to mechanical systems, existing literature lacks a systematic hybrid methodology that combines the strengths of different optimization techniques specifically for SCM design. Most studies rely on single optimization methods or sequential application of other various algorithms without proper integration, limiting their effectiveness in finding optimal solutions in complex, multi-dimensional parameter spaces.

An alternative approach involves integrating springs into SCMs to reduce unbalanced forces and moments. Prior research has shown the potential of springs to balance dynamic forces while maintaining compact designs partially [5]. Groza and Antonya [7,8] demonstrated the effectiveness of dynamically spring-balanced SCMs for reciprocating machines, while Frischknecht and Howell [9] introduced spring elements into SCMs to develop constant-force compliant mechanisms. Tarnita [10] and Baokun [11] further explored the geometric and elastic characteristics of springs in SCMs. Despite these advancements, most existing studies focus on specific spring configurations, such as torsional springs or extension springs with fixed insertion points, leaving a critical gap in understanding how the placement and stiffness of springs influence dynamic behavior.

Building on this foundation, the authors of a preliminary study [1] presented an initial exploration of SCM optimization with spring integration, specifically in the context of a wood splitter application. While this work demonstrated springs’ potential to improve performance, it lacked a detailed mathematical model. It did not analyze the influence of spring placement and stiffness on key dynamic parameters such as energy consumption, torque, and reaction forces. These limitations underscore the need for a more thorough investigation.

This study presents two key innovations to address the identified research gaps:

First, we propose a novel approach to integrating springs into the slider-crank mechanism with optimized placement and stiffness parameters. Unlike previous studies that used springs in fixed configurations, our approach systematically evaluates various spring insertion points and stiffness values to identify optimal configurations. This innovation enables significant reductions in dynamic loads while maintaining or improving operational efficiency, representing a substantial advancement over conventional SCM designs.

Second, we introduce a novel hybrid optimization approach combining the Conjugate Direction with the Orthogonal Shift (CDOS) method and Parameter Space Investigation (PSI). This innovative methodology leverages the complementary strengths of both algorithms: CDOS’s ability to efficiently identify promising regions in the parameter space and PSI’s capability to investigate these regions to find optimal solutions thoroughly. Our hybrid approach addresses the limitations of existing optimization methods by providing more comprehensive and efficient multi-objective optimization for complex mechanical systems.

This study aims to address these gaps by presenting an enhanced mathematical model for SCMs with spring integration. The model incorporates critical design parameters, including the insertion points and stiffness of the spring, and evaluates their impact on dynamic performance metrics. Additionally, the study employs the hybrid CDOS-PSI algorithm to perform multi-objective optimization. A detailed analysis, supported by 3D visualizations, reveals intriguing configurations where spring-assisted SCMs achieve superior performance compared to non-spring designs. These insights are validated through comparative simulations with Siemens NX software and further applied to optimize the wood splitter mechanism. This study provides valuable guidance for designing and optimizing SCMs in various mechanical systems by bridging the gap between theoretical modeling and practical application.

## 2. Dynamic of the slider-crank mechanism with Spring

**Fig 1:**
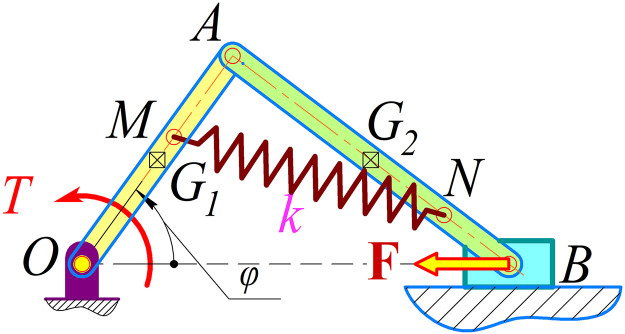
The diagram of the slider crank mechanism system with spring application [1].

**Fig 2:**
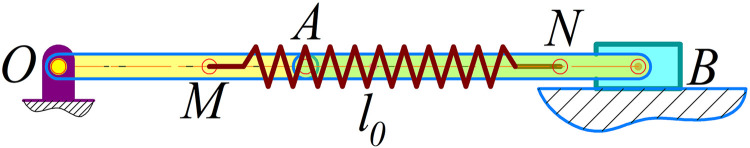
Non-deformed position of spring of the mechanism [1].

The diagram of the slider crank mechanism system with spring application is shown in Fig 1:

The configuration of the slider-crank mechanism incorporating a spring is illustrated in Fig 1. In this setup, *OA* represents the driving link with a length *l*_1_, mass *m*_1_, applied torque *T*, angular velocity *ω*_*OA*_, and angular acceleration *ε*_*OA*_. The angle *Φ* denotes the orientation of the driving link relative to the *x*-*axis*. The connecting rod *AB* has a length of l_2_ and a mass of m_2_ and is connected to slider B, which has a mass of m_3_. A spring, designated *MN*, is characterized by a stiffness constant *k*, with one end fixed at point *M* on the driving link *OA* and the other at point *N* on the connecting rod *AB*. The spring is undeformed when the two links, OA and AB, are collinear, corresponding to an initial length of l0 (Fig 2).

Key ratios are as follows: *β* = *AB*/*OA* = *l*_2_*/l*_1_, *α*_1_ = *OM/OA*, and *α*_2_ = *AN/AB*. The geometry of *OA* and *AB* can vary depending on their three-dimensional structure (e.g., flywheels or crankshafts). To determine the positions of the centers of gravity *G*_1_ and *G*_2_, the distances *OG*_1_ and *AG*_2_ are used, respectively. The moments of inertia about the axes *G*_1_*z* and *G*_2_*z* (perpendicular to the plane in Fig 1) are denoted as *J*_1_ and *J*_2_. During operation, slider *B* moves along its path under friction *μ* and an external force **F**.

The fundamental relationships required to evaluate the dynamic of the slider-crank mechanism with spring are presented as follows:

Location coordinates of points *O*, *A*, *B*, *G*_1_, *G*_2_, *M*, *N*:


$$\begin{array}{c} x_{O}=y_{O}=y_{B}=0;\;\;x_{A}=l_{1}\cos\varphi ;\;\;\;y_{A}=l_{1}\sin\varphi \;\;;x_{B}=l_{1}\cos\varphi +\sqrt{l22-{\left(l_{1}\sin\varphi \right)}^{2}}\\ x_{G_{1}}=OG_{1}\cos\varphi ;\;\;\;\;y_{G_{1}}=OG_{1}\sin\varphi ;\;\;x_{G_{2}}=x_{A}+AG_{2}\sqrt{1-\frac{l12}{l22}\sin^{2}\varphi };\;\;\;\;y_{G_{2}}=\left(1-\frac{AG_{2}}{l_{2}}\right)\cdot y_{A}\\ x_{M}=\alpha_{1}l_{1}\cos\varphi ;\;\;\;\;y_{M}=\alpha_{1}l_{1}\sin\varphi ;\;\;x_{N}=l_{1}\cos\varphi +\alpha_{2}\sqrt{l22-{\left(l_{1}\sin\varphi \right)}^{2}};\;\;\;y_{N}=\left(1-\alpha_{2}\right)l_{1}\sin\varphi \end{array}$$

Speed of slider *B*: $v_{B}=-\left(1+\frac{l_{1}\cos\varphi }{\sqrt{l22-{\left(l_{1}\sin\varphi \right)}^{2}}}\right)\cdot \omega_{OA}l_{1}\sin\varphi$

Acceleration of slider *B* and points *G*_1_, *G*_2_:


aB=((l22−l12)(l1sinφ)2[l22−(l1sinφ)2]\raise0.7ex3/32\nulldelimiterspace\lower0.7ex2−l1cosφ−(l1cosφ)2l22−(l1sinφ)2)·(ωOA)2−(1+l1cosφl22−(l1sinφ)2)·l1sinφ·εOA;aG1x=−OG1·(εOAsinφ+ωOA2cosφ);aG1y=OG1·(εOAcosφ−ωOA2sinφ);

aG2x=−(l1cosφ+(l12sin4φ+l22cos2φ)AG2l12l2[l22−(l1sinφ)2]\raise0.7ex3/32\nulldelimiterspace\lower0.7ex2)(ωOA)2−(1+AG2l1cosφl2l22−(l1sinφ)2)l1sinφεOA;aG2y=(1−AG2l2)l1(cosφεOA−sinφωOA2);

Angular acceleration of the connecting rod *AB*:εAB=(l22−l12)l1sinφ[l22−(l1sinφ)2]\raise0.7ex3/32\nulldelimiterspace\lower0.7ex2·(ωOA)2−l1cosφl22−(l1sinφ)2·εOA

The undeformed length and the working length of the spring are determined using the following equations:


$$l_{0}=\left(1-\alpha_{1}\right)l_{1}+\alpha_{2}l_{2};\;\;MN=l=\sqrt{{\left(x_{N}-x_{M}\right)}^{2}+{\left(y_{N}-y_{M}\right)}^{2}}$$

Based on this, the spring’s deformation and elastic force are determined as follows: Δ*l* = *l*–*l*_0_; *F*_elastic_ = *k*‧Δ*l*. Using the kinematic values derived above, the corresponding dynamic equations for the system are established as follows.


$$\begin{array}{c} \cdot \;\;X_{O}+X_{A}+F_{elastic}\cdot \frac{x_{N}-x_{M}}{MN}=m_{1}a_{G_{1}x}\\ \cdot \;\;Y_{O}+Y_{A}-m_{1}g+F_{elastic}\cdot \frac{y_{N}-y_{M}}{MN}=m_{1}a_{G_{1}y}\\ \cdot \;\;T+\left(x_{A}-x_{G_{1}}\right)\cdot Y_{A}-\left(y_{A}-y_{G_{1}}\right)\cdot X_{A}+\left(x_{O}-x_{G_{1}}\right)\cdot Y_{O}-\left(y_{O}-y_{G_{1}}\right)\cdot X_{O}\\ +\left(x_{M}-x_{G_{1}}\right)\cdot F_{elastic}\cdot \frac{y_{N}-y_{M}}{MN}-\left(y_{M}-y_{G_{1}}\right)\cdot F_{elastic}\cdot \frac{x_{N}-x_{M}}{MN}=J_{1}\varepsilon_{OA}\\ \cdot \;\;-X_{A}+X_{B}+F_{elastic}\cdot \frac{x_{M}-x_{N}}{MN}=m_{2}a_{G_{2}x}\\ \cdot \;\;-Y_{A}+Y_{B}-m_{2}g+F_{elastic}\cdot \frac{y_{M}-y_{N}}{MN}=m_{2}a_{G_{2}y} \end{array}$$

$$\begin{array}{c} \cdot \;\;\left(x_{A}-x_{G_{2}}\right)\cdot \left(-Y_{A}\right)-\left(y_{A}-y_{G_{2}}\right)\cdot \left(-X_{A}\right)+\left(x_{B}-x_{G_{2}}\right)\cdot Y_{B}-\left(y_{B}-y_{G_{2}}\right)\cdot X_{B}\\ +\left(x_{N}-x_{G_{2}}\right)\cdot F_{elastic}\cdot \frac{y_{M}-y_{N}}{MN}-\left(y_{N}-y_{G_{2}}\right)\cdot F_{elastic}\cdot \frac{x_{M}-x_{N}}{MN}=J_{2}\varepsilon_{AB}\\ \cdot \;\;Y_{B}+m_{3}g=N_{B}\\ \cdot \;\;-X_{B}+F_{friction}+F=m_{3}a_{B}\\ \cdot \;\;F_{friction}=-\mu \cdot \left|N_{B}\right|\cdot sign\left(v_{B}\right) \end{array}$$

This represents a system of nonlinear algebraic equations with eight unknowns {*X*_*O*_, *Y*_*O*_, *X*_*A*_, *Y*_*A*_, *X*_*B*_, *Y*_*B*_, *N*_*B*_, *T*}, where *X*_*O/A/B*_ and *Y*_*O/A/B*_ correspond to the reaction forces at the hinge joints *O*, *A,* and *B* in the *x* and *y*-directions, respectively. These equations can be solved using numerical approaches, such as the Sharma and Gupta methods [12].

## 3. Multi-objective design optimization of the SCM with Spring in the wood splitter

### 3.1. Multi-objective model of the SCM with spring in the wood splitter

**Fig 3:**
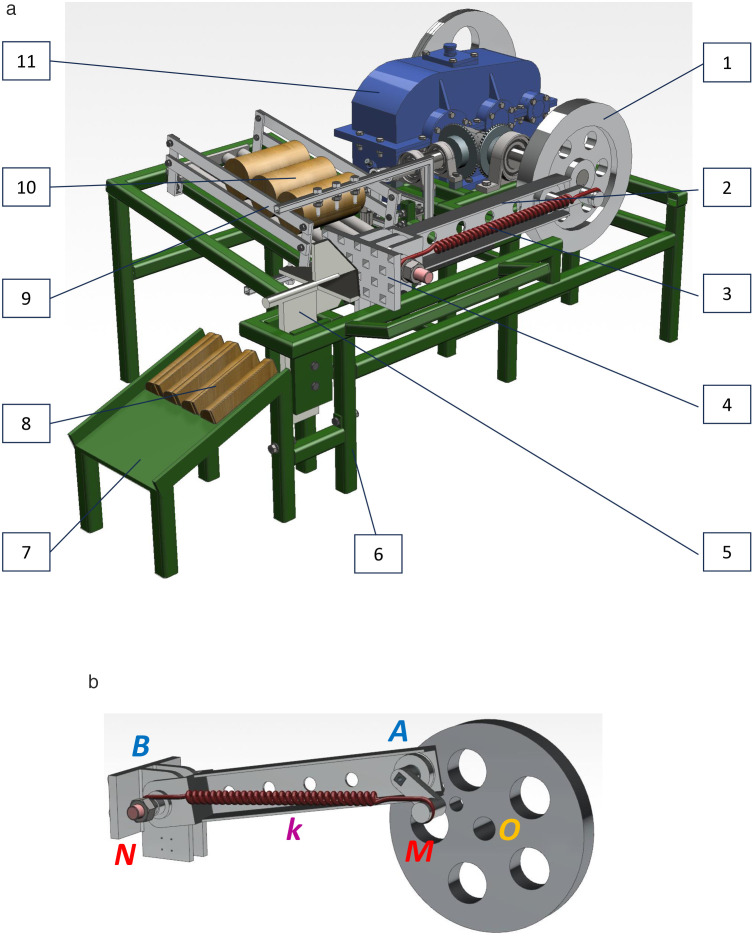
(a) wood splitter: 1 – flywheel (driving link); 2 – connecting rod; 3 – springs; 4 – slider (pressing plate); 5 – split knife; 6 – machine body frame; 7 – trough to drain firewood; 8 – firewood after being split; 9 – firewood supply structure; 10 – firewood before being split; 11 – gearbox; (*b*) Actuator structure of wood splitter: the slider crank mechanism system with spring application [1].

The slider-crank mechanism (SCM) is extensively used in industrial and agricultural machinery, with the wood splitter being one notable application. Firewood remains a vital fuel source in Vietnam and many other parts of the world. Processed wood waste is often repurposed as fuel for traditional bakery ovens or heating during winter in colder regions. Furthermore, charcoal derived from burned wood has diverse applications, including its use as a plant substrate, in producing gunpowder and fireworks, as a desiccant material, fertilizer, antioxidant, and even in skincare products. The structural design of the wood splitter incorporating a spring-based mechanism is illustrated in Fig 3.

The working principle of the wood splitter (Fig 3-a) operates as follows: Firewood (10), shaped as round pillars, is placed in the inclined chute of the firewood supply mechanism (9) to await processing. Torque from the motor is transmitted through the gearbox (11) to the flywheel (1), causing it to rotate. This rotation drives the connecting rod (2), which moves the presser, designed as a slider (4), to push the firewood deeper into the splitting knife (5). The wood is then split into four pieces (8) and dropped into the trough (7) for collection. The entire machine is mounted on a sturdy frame (6).

A spring (3) is integrated into the mechanism (Fig 3-b), where one end is fixed at a specific point on the flywheel (1) and the other end on the connecting rod (2). Since the wood splitter uses a slider-crank mechanism, structurally similar to the one in Fig 1, the optimization task focuses on determining the parameters *β* = *AB/OA* = *l*_2_*/l*_1_; *α*_1_ = *OM/OA*; *α*_2_ = *AN/AB* and the spring stiffness *k*. These parameters are optimized to minimize dynamic objectives, including energy consumption *A*, maximum torque *T*_*max*_, maximum reaction force at joint A *R*_*Amax*_, and maximum slide reaction *N*_*Bmax*_.

In the primary mechanism of the wood splitter, the following parameters are applied to the mathematical model: *l*_1_ = 175 mm; *l*_2_ = 580 mm; *ω*_*OA*_ = 30 rpm = const; *ε*_*OA*_ = 0; *m*_1_ = 41.5147 kg; *m*_2_ = 7.656875 kg; *m*_3_ = 9.8996 kg; *μ* = 0.3; *OG*_1_ = 1.9 mm; *AG*_2_ = 316.5732 mm; *J*_1_ = 0.889678 kg.m^2^, and *J*_2_ = 0.620448 kg.m^2^. The splitting force, which corresponds to certain types of firewood (such as rubber, eucalyptus, and cashew) with diameters ranging from Ø = 10–15 cm, has been experimentally determined in [13] to be *F* = 3.233 kN. For denser wood species and larger log diameters, further testing following the methodology outlined in the referenced study is required to determine the appropriate splitting force. The parameter ranges under investigation include *α*_1_ = 0...1; *α*_2_ = 0..1; *k* = 0...20000 N/m. The parameter ranges for *α*_1_ and *α*_2_ are determined by the spring’s installation points on the driving link *OA* and the connecting rod *AB*. When *α*_1_ and *α*_2_ lie between 0 and 1, the spring attachment points *M* or *N* remain within each respective link. Although values below 0 or above 1 are theoretically possible, they would lengthen the driving link or connecting rod beyond the limits of the wood splitter’s design. The selected spring stiffness range (*k*) is based on the typical stiffness values of commercially available springs.

The mathematical model for designing the mechanism is structured as follows:

a) Design parameters: $𝐱={\left\{x_{1},x_{2},x_{3},x_{4}\right\}}^{T}={\left\{\alpha_{1},\alpha_{2},\beta ,k\right\}}^{T}$ with range constraints: *i*=1..4
b) The performance objectives for the mechanism are defined as follows: *T*_*max*_ → min; *A* → min; {*R*_*Omax*_, *R*_*Amax*_, *R*_*Bmax*_}→min; *N*_*Bmax*_→min. Here, *T*_*max*_ represents the peak torque of the driving link during one complete rotation [N.m]. Minimizing this value allows selecting a motor with lower capacity, ultimately reducing the machine’s overall cost. Objective *A* corresponds to the energy (or work) the motor consumes over one revolution (J). Lowering *A* directly reduces the mechanism’s energy consumption. The terms {*R*_*Omax*_, *R*_*Amax*_, *R*_*Bmax*_} denote the maximum reaction forces [N] at joints *O*, *A,* and *B* within a single rotation period. Reducing these reactions enhances the machine’s durability by mitigating stress at critical joints. Finally, *N*_*Bmax*_ is the maximum reaction force acting on the slider [N]. A smaller *N*_*Bmax*_ value minimizes friction and energy loss, improving mechanism efficiency.

For each set of design parameters **x**={*x*_1_,*x*_2_,*x*_3_,*x*_4_}^*T*^={*α*_1_, *α*_2_, *β*, *k*}^*T*^, the equations must be solved 360 times, representing 360 discrete positions of the driving link during one full rotation. The maximum values for *T*_*max*_, {*R*_*Omax*_, *R*_*Amax*_, *R*_*Bmax*_}, and *N*_*Bmax*_ are extracted from these 360 results for each parameter. The energy consumption *A* is determined through integration concerning the angular position *Φ*.


$$\begin{array}{c} T_{\max}=\max\left|T\left(\varphi \right)\right|;\varphi =0^{\circ }.{\mathrm{.359}}^{\circ };\;A=\int_{t_{1}}^{t_{2}}Pdt=\omega_{OA}\int_{t_{1}}^{t_{2}}T\left(\varphi \right)dt=\frac{\pi }{180^{\circ }}\int_{0^{\circ }}^{359^{\circ }}T\left(\varphi \right)d\varphi \\ R_{\left\{O/A/B\right\}\max}=\max\sqrt{X_{\left\{O/A/B\right\}}^{2}\left(\varphi \right)+Y_{\left\{O/A/B\right\}}^{2}\left(\varphi \right)};\varphi =0^{\circ }.{\mathrm{.359}}^{\circ };\;\;N_{B\max}=\max\left|N_{B}\left(\varphi \right)\right|;\varphi =0^{\circ }.{\mathrm{.359}}^{\circ } \end{array}$$

The presented mathematical model represents a complex multi-objective framework. The mechanism includes three hinge joints – *O*, *A,* and *B*. However, joints *O* and *B* are typically connected to components made from robust materials, such as flywheels or sliding steel blocks, making them more durable. In contrast, joint *A*, which connects two moving links, requires weight reduction and is, therefore, more stress-resistant. To streamline the mathematical model, the reaction force *R*_*A*_ at hinge *A* is prioritized for consideration, while reactions at joints *O* and *B* are excluded from detailed analysis.

### 3.2. Influence of spring placement and stiffness on dynamic performance of the SCM with spring in the wood splitter

**Fig 4:**
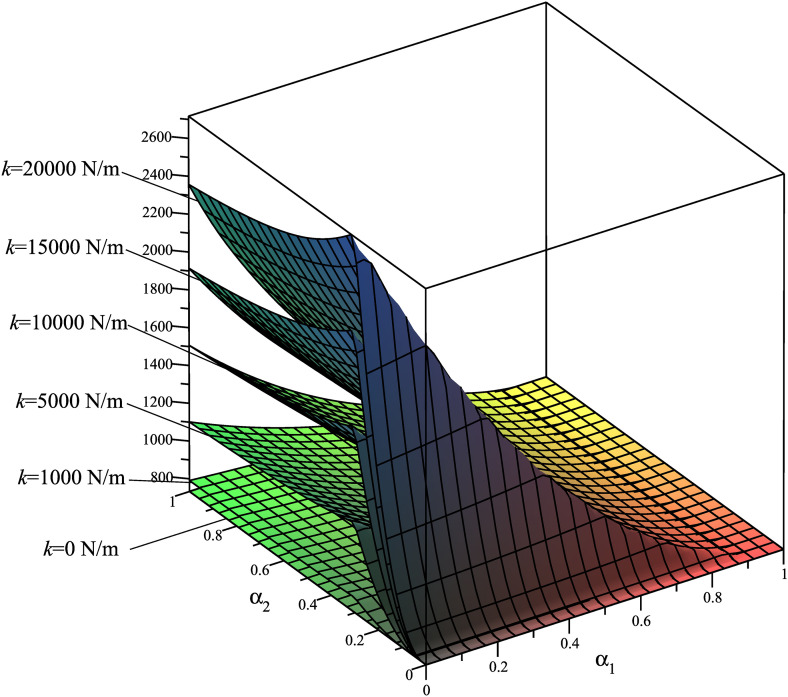
Surfaces Φ_1_(*α*_1_,*α*_2_)=*A*(*α*_1_,*α*_2_) [J].

**Fig 5:**
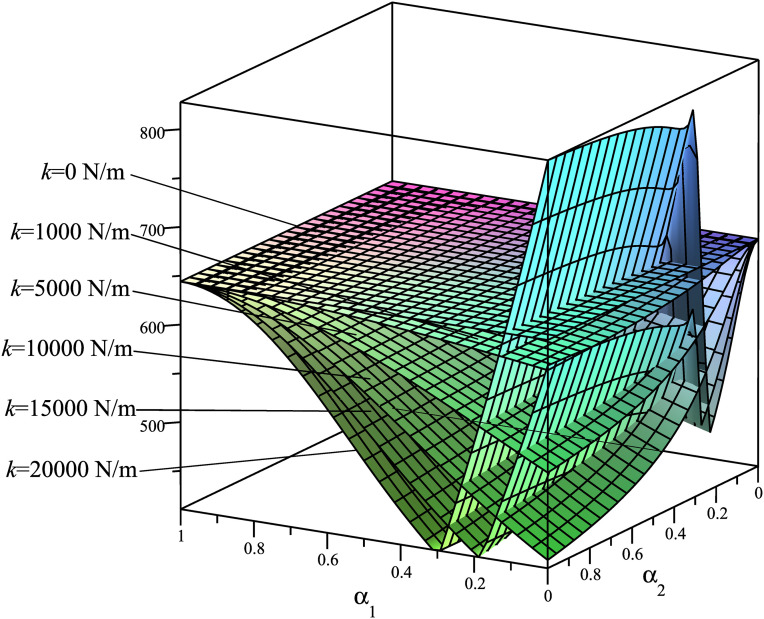
Surfaces Φ_2_(*α*_1_,*α*_2_)=*T*_*max*_(*α*_1_,*α*_2_) [N.m].

**Fig 6:**
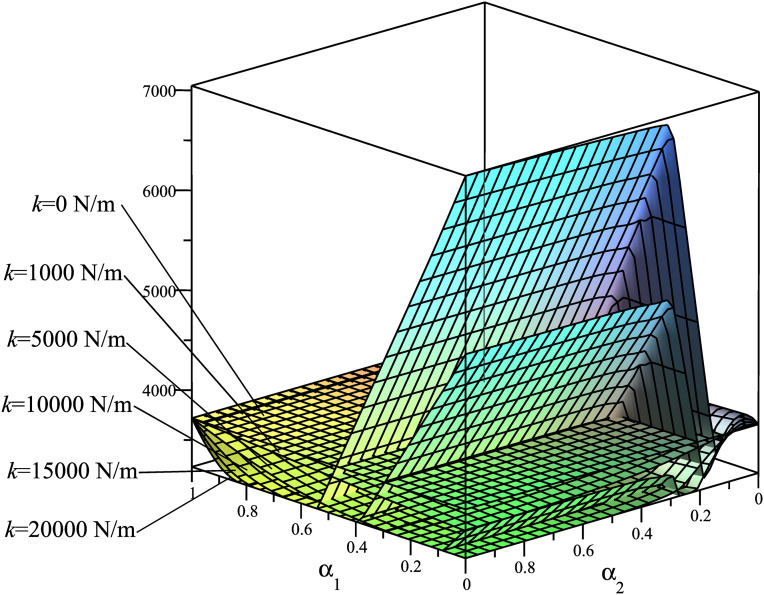
Surfaces Φ_3_(*α*_1_,*α*_2_)=*R*_*Amax*_(*α*_1_,*α*_2_) [N].

**Fig 7:**
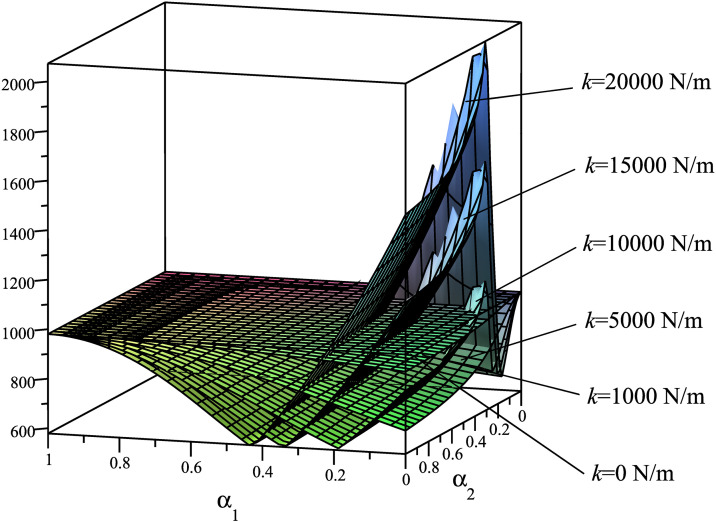
Surfaces Φ_4_(*α*_1_,*α*_2_)=*N*_*Bmax*_(*α*_1_,*α*_2_) [N].

Using the mathematical model described in Section 3.1, we analyze the dependency of the criteria **Φ**={Φ_1_ = *A*; Φ_2_ = *T*_*max*_; Φ_3_ = *R*_*Amax*_; Φ_4_ = *N*_*Bmax*_} on the spring placement, considering six different stiffness values: *k*_1_ = 0 N/m, *k*_2_ = 1000 N/m, *k*_3_ = 5000 N/m, *k*_4_ = 10000 N/m, *k*_5_ = 15000 N/m, *k*_6_ = 20000 N/m. The results are presented in Figs 4–7, demonstrating how these parameters influence the system’s dynamic behavior.

These rule surfaces are only intuitive about the relationship among the four objectives **Φ**={Φ_1_ = *A*; Φ_2_ = *T*_*max*_; Φ_3_ = *R*_*Amax*_; Φ_4_ = *N*_*Bmax*_} and with spring mounting positions and with six distinct *k* values. Looking at these graphs, we have the following observations:

Fig 4 illustrates the graph of Φ_1_(*α*_1_,*α*_2_)=*A*(*α*_1_,*α*_2_). It is observed that as *α*_1_ approaches 1 (i.e., as the left mounting point *M* of the spring gradually moves closer to the hinge *A*), the energy consumption decreases. Conversely, when the left mounting point *M* of the spring progressively shifts toward the rotation axis *O* of the driving link, the energy consumption increases; furthermore, a higher spring stiffness *k* results in correspondingly greater energy consumption.

Based on the graph of Φ_2_(*α*_1_,*α*_2_)=*T*_*max*_(*α*_1_,*α*_2_) (Fig 5), it is observed that a higher spring stiffness *k* increases the likelihood of attaining the minimum value of *T*_*max*_. In particular, the stiffness *k* lies in the range of 12000–20000 N/m, the parameter *α*_2_ is equal to 1 (i.e., the correct mounting point *N* of the spring is located exactly at the sliding block *B*), and the parameter *α*_1_ is within the range of 0.1 ÷ 0.4. Moreover, it is noted that the difference between the *T*_*max*_ value without the spring and the minimum *T*_*max*_ value with the spring is approximately 650–400 = 250 N·m, while the difference between the *T*_*max*_ value without the spring and the maximum *T*_*max*_ value with the spring is about 850–650 = 200 N·m. This indicates that a reduction of approximately 55.5% in *T*_*max*_ can be achieved when the spring is judiciously applied in the SCM.

Based on the graph of Φ_3_(*α*_1_,*α*_2_)=*R*_*Amax*_(*α*_1_,*α*_2_) (Fig 6), it is observed that when the spring stiffness *k* is low (below 1000 N/m), the minimum value of *R*_*Amax*_ is concentrated at *α*_1_ = 0 and *α*_2 _= 1 (i.e., the left mounting point *M* of the spring moves progressively closer to the rotation axis *O* of the driving link, while the right mounting point *N* of the spring shifts toward the sliding block *B*). When the spring stiffness *k* increases to 5000 N/m or higher, the minimum *R*_*Amax*_ is concentrated within the *α*_1_ range of 0.1 to 0.8. The minimum *R*_*Amax*_ for different values of *k* is approximately within the range of 3232–3400 N. This suggests that an appropriate use of the spring can reduce the reaction force at joint *A* by 12.8%.

Based on the graph of Φ_4_(*α*_1_,*α*_2_)=*N*_*Bmax*_(*α*_1_,*α*_2_) (Fig 7), it is observed that when the spring stiffness *k* is low (below 1000 N/m), the minimum value of *N*_*Bmax*_ is concentrated at *α*_1_ = 0 and *α*_2 _= 1 (i.e., the left mounting point *M* of the spring moves progressively closer to the rotation axis *O* of the driving link, while the right mounting point *N* of the spring shifts toward the sliding block *B*). When the spring stiffness *k* increases to 5000 N/m or higher, the minimum *N*_*Bmax*_ is concentrated within the *α*_1_ range of 0.2 to 0.5. This indicates that an appropriate use of the spring can reduce the reaction force at the sliding block *B* by 25.3%.

Thus, it can be concluded that all the dynamic parameters of the SCM can be optimized (minimized) if the spring is utilized with an appropriate stiffness and placement. The greater the stiffness *k* of the spring, the higher the maximum values of all the objective functions; however, the minimum values of the criteria tend to converge. Nevertheless, these graphs alone are insufficient to determine the optimal value, as this is a multi-objective optimization problem that also considers functional constraints, along with the stiffness parameter *k*, which varies from 0 to 20000 N/m.

### 3.3. Hybridization and combination of the CDOS–PSI method for multi-objective design optimization

#### 3.3.1. Introduction to CDOS and PSI optimization methods.

In increasingly complex engineering design optimization problems, selecting and combining appropriate optimization methods has become crucial. To effectively address multi-objective optimization problems, this research examines two powerful methods: CDOS (Conjugate Direction with Orthogonal Shift) and PSI (Parameter Space Investigation), each with distinct advantages that complement each other when combined.

#### 3.3.2. CDOS optimization method.

**Fig 8:**
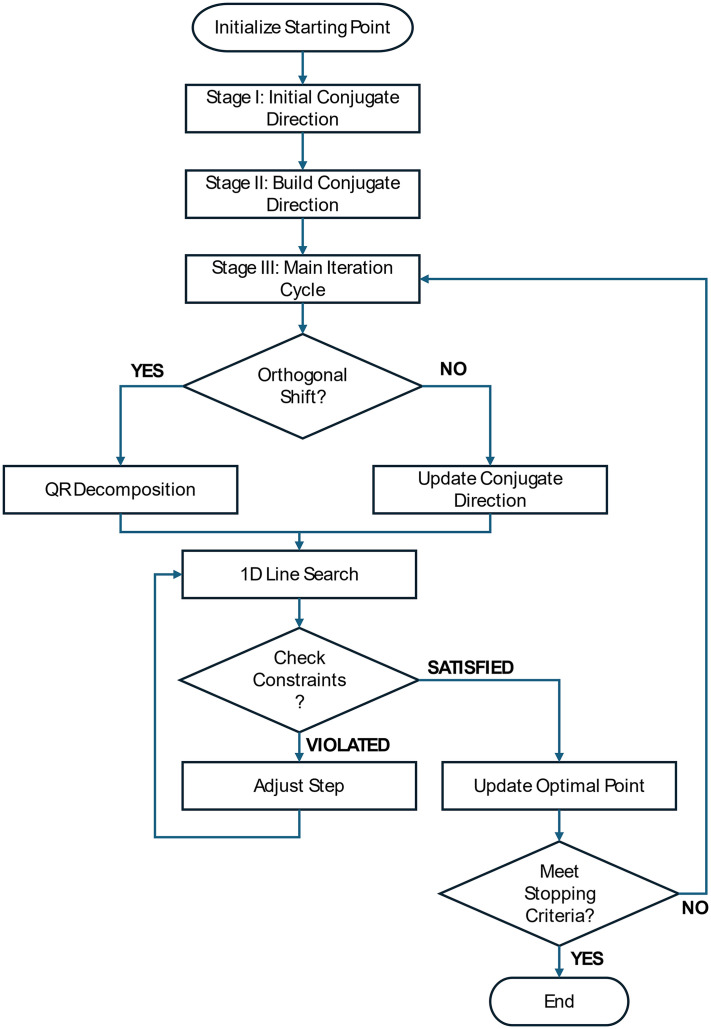
Flowchart of the CDOS (Conjugate Direction with Orthogonal Shift) Algorithm.

The CDOS (Conjugate Direction with Orthogonal Shift) method [14], as illustrated in Fig 8, is an efficient single-objective optimization technique with quadratic convergence for quadratic and near-quadratic functions. CDOS is a derivative-free method specifically developed to solve optimization problems where the objective function and constraints are black boxes. A significant advantage of this method is its ability to handle non-differentiable and non-continuous objective functions, while constraints can also be non-differentiable and non-continuous.

The flowchart in Fig 8 shows that the CDOS algorithm consists of three main stages. The first stage initializes the starting point, followed by Stage I, which determines the initial conjugate direction based on a quasi-gradient. Stage II builds conjugate directions using an orthogonal shift method. Finally, Stage III performs the primary iteration cycle to update the constructed conjugate directions.

The algorithm checks whether an orthogonal shift is needed in each iteration cycle. If required, the algorithm performs QR decomposition; otherwise, it updates the conjugate direction. Subsequently, the algorithm conducts a one-dimensional line search along the conjugate direction. If the constraint check is violated, the step is adjusted. If constraints are satisfied, the optimal point is updated. This process repeats until the stopping criteria are met.

A notable feature of CDOS is that it is not entirely greedy, allowing movement in directions that do not contain the current extremum point. This characteristic extends the method into an effective global optimization tool.

#### 3.3.3. PSI optimization method.

**Fig 9:**
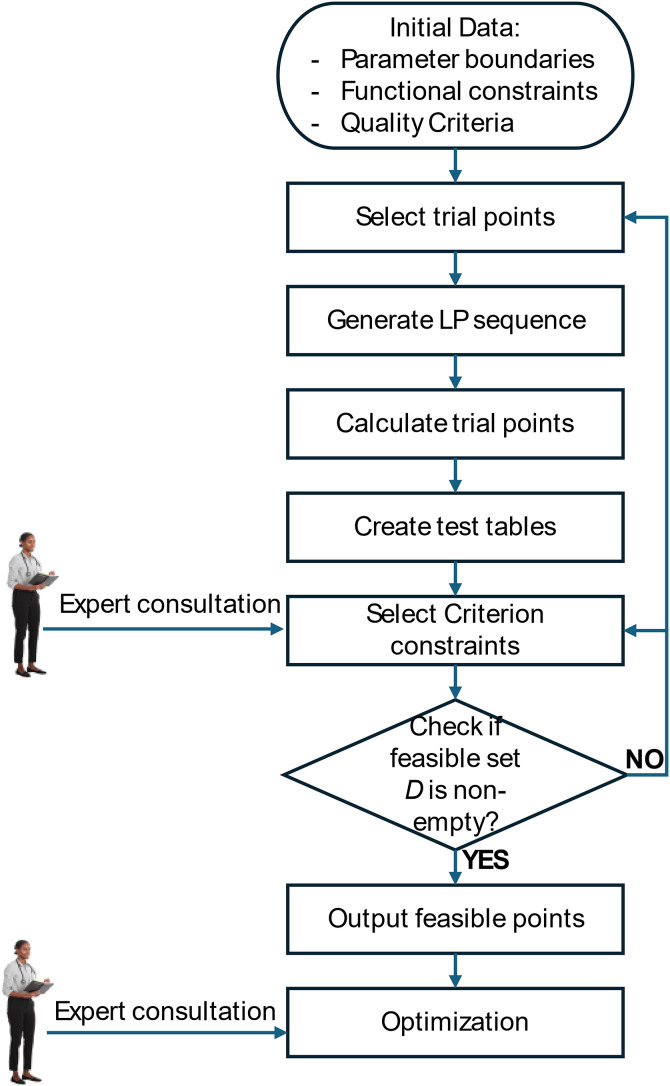
Flowchart of the PSI (Parameter Space Investigation) Algorithm.

The PSI (Parameter Space Investigation) method [15], as illustrated in Fig 9, is a parameter space exploration technique designed explicitly for multi-objective optimization problems. Unlike traditional multi-objective optimization algorithms, PSI does not aim to find a single Pareto solution set that forces users to accept but instead provides a visual representation of feasible and Pareto-distributed solutions.

As depicted in the flowchart in Fig 9, PSI begins with initial data, including parameter boundaries, functional constraints, and quality criteria. The process involves selecting trial points, generating an LP sequence, calculating the trial points, and creating test tables. Through expert consultation, criterion constraints are chosen, and the next step is to check if the set of feasible points *D* is non-empty.

A key characteristic of PSI is its use of uniformly distributed sequences (ЛПτ-sequences or LP-sequences) to populate the parameter space with a limited number of test points. The number of these points depends on the computational time required to evaluate the objective function vector and the constraint function, as well as the acceptable waiting time for the user. The process involves comparing constraint functions with predefined limits and assessing the values of the same criterion to identify feasible and optimal solutions.

If set D is non-empty, the process continues with outputting feasible points and optimizing with expert consultation. If set *D* is empty, it is necessary to return to adjust parameters and boundaries, generate a new LP sequence, and recalculate.

The strength of PSI lies in its interactive nature, allowing users to analyze, select, and customize optimal solutions based on specific criteria. This flexibility assists users in dynamically adjusting mathematical models to match practical engineering constraints and actual operating conditions. However, one limitation of PSI is that improving the quality of the optimal solution necessitates increasing the number of test points, which results in a higher computational cost.

#### 3.3.4. Integration of CDOS and PSI for multi-objective optimization.

The next step involves applying the CDOS-PSI hybrid approach to determine the optimal design parameters for the actuator of the wood splitter, which operates based on the slider-crank mechanism incorporating a spring.

**Fig 10:**
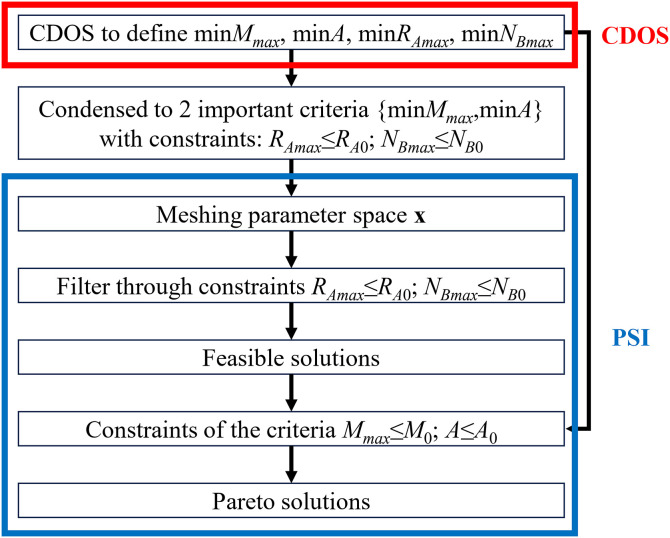
Diagram of the CDOS-PSI hybrid approach for multi-objective optimization.

Considering the distinct advantages of both methods, this paper proposes their hybridization and integration to address the multi-objective optimization problem, as illustrated in Fig 10.

The CDOS method is initially applied to determine the minimum value of each objective, independently providing insight into the best possible outcome for each criterion without considering the influence of other objectives. If the CDOS method is not utilized initially to determine the minimum values of the objective functions, the PSI method would require significantly more time to subdivide the parameter space using a massive number of trial points, and the calculated results would still be inferior. Therefore, CDOS plays a critical role in establishing threshold values for the objective functions within the PSI method. Following this, the two reaction-based objectives, *R*_*A*_ and *N*_*B*_, are transformed into constraints, as optimizing these criteria is not particularly meaningful. Instead, the focus shifts to optimizing two key factors related to the machine’s energy consumption and cost. The PSI method is utilized to address the multi-objective nature of the problem. The process begins by discretizing the parameter space using a uniformly distributed sequence of test points. These test points are then used to compute the values of both objective and constraint functions. Any test points that do not satisfy the constraints are eliminated, leaving only feasible solutions. The resulting dataset provides an overview of the achievable objective values. A Pareto set is obtained by selecting desired values for both objectives, representing solutions that cannot be improved across all criteria simultaneously. The final selection among these solutions depends on user preferences and the judgment of design experts.

However, when dealing with real-world data, an initial observation can be made: the larger the parameter *β*, the more effectively the criteria are satisfied. This implies that increasing the length of the connecting rod yields better performance. Nevertheless, the mechanism’s available working space constrains the connecting rod length. This insight simplifies the problem by reducing the design parameters under consideration. As a result, the optimization problem is ultimately reduced to three design parameters: {*α*_1_, *α*_2_, *k*}.

## 4. Result and discussion

**Step 1**: Use CDOS to find min*A*, min*T*_*max*_, min*R*_*Amax*_, min*N*_*Bmax*_:

Table 1 presents four solutions, each corresponding to the optimal value of a specific criterion: min*A*, min*T*_*max*_, min*R*_*Amax*_, and min*N*_*Bmax*_. These optimal values are highlighted in bold and blue and have been determined using the CDOS method. Simultaneously, the values of the remaining three objectives are computed and displayed in the same row for each optimal solution.

**Table 1:** Optimal solution according to each objective.

Optimal solution	Parameters	min*A*	min*T*_*max*_	min*R*_*Amax*_	min*N*_*Bmax*_
Without spring	*k* = 0	**733.346**	**644.1**	**3720**	**980.465**
min*A*	α1 = 0.9174, *α*_2_ = 1, *k* = 12005.2	**732.4144** (–0.13%)	641 (–0.482%)	3504.655 (–5.96%)	974.256 (–0.64%)
min*T*_*max*_	α_1_ = 0.278, *α*_2_ = 1, *k* = 18998	1468.29 (+66.8%)	**405.78** (–45.4%)	4814.71 (+25.7%)	811.2 (–18.9%)
min*R*_*Amax*_	*α*_1_ = 0.402, *α*_2_ = 1, *k* = 15354.4	1116.45 (+41.4%)	482.34 (–28.72%)	**3231.72** (–14.05%)	622.22 (–44.7%)
min*N*_*Bmax*_	*α*_1_ = 0.429, *α*_2_ = 1, *k* = 20000	1192.6 (+47.7%)	458.35 (–33.7%)	4011.93 (+7.55%)	**570.54** (–52.9%)

The table further includes the percentage improvement of each objective compared to the initial case (without a spring). The parentheses values indicate each criterion’s relative change when incorporating a spring. For instance, optimizing *T*_*max*_ leads to a 45.4% reduction, but other objectives may experience an increase or decrease in value. This underscores the nature of multi-objective optimization, where enhancing one parameter may negatively impact others.

Additionally, it is observed that higher spring stiffness increases its impact on the dynamic parameters. However, increasing stiffness does not always yield universally favorable results; it requires careful selection to balance multiple competing factors.

Table 1 also suggests that placing the spring’s correct attachment point (N) at slider *B* (*α*_2_ = 1) is optimal when considering individual objectives separately. However, when evaluating the overall system–accounting for multiple constraints and real-world effects such as friction (*μ*)–a more comprehensive analysis is necessary to determine the most effective design choice.

**Fig 11:**
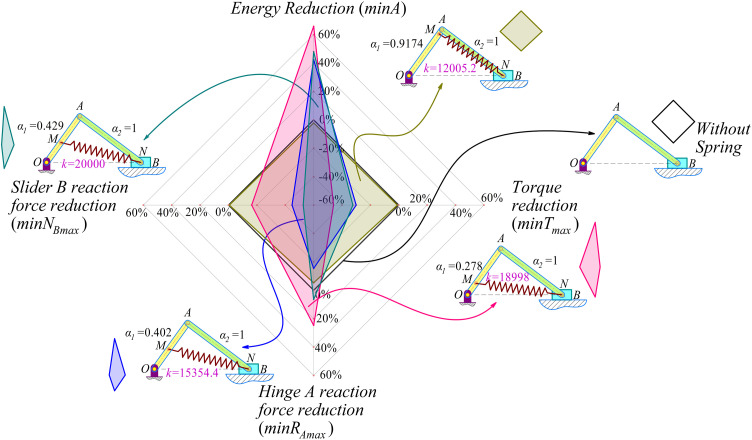
Radar chart comparing dynamic criteria of the slider-crank mechanism with Springs.

The radar chart below (Fig 11) compares the dynamic indicators of a slider-crank mechanism under 5 cases, including the baseline case without a spring and four other optimized designs for different dynamic objectives.

- Black-bordered square: This represents the slider-crank mechanism without a spring, with all indicators connected at the four vertices with values set to 0%. This serves as a reference for comparison with the optimized designs using springs.
- Gold-colored quadrilateral: This configuration uses a spring with parameters *α*_1_ = 0.9174, *α*_2_ = 1, *k* = 12005.2, optimized to minimize energy consumption. The shape’s vertices have values of –0.13%, –0.482%, –5.96%, and –0.64%. All indicators show improvement compared to the mechanism without a spring; however, the magnitude of improvement is relatively small.
- Pink quadrilateral: In this design, the spring with *α*_1_ = 0.278, *α*_2_ = 1, *k* = 18998 is optimized to minimize the maximum torque. The vertices have values at +66.8%, –45.4%, + 25.7%, –18.9%. With this design, the torque indicator improves by 45.4%, and the reaction force at the slider *B* is reduced by 18.9%. However, energy consumption increases by 66.8%, and the hinge reaction force at hinge *A* deteriorates by 25.7%.
- Sky-blue quadrilateral: This design uses a spring with *α*_1_ = 0.402, *α*_2_ = 1, *k* = 15354.4, optimized to minimize the reaction force at hinge *A*. The achieved values are + 41.4%, –28.7%, –14.05%, –44.7%. This configuration reduces the torque by 28.72%, the reaction force at hinge A by 14.05%, and the reaction force at slider *B* by 44.7%. However, energy consumption increases by 41.4%.
- Deep-blue quadrilateral: This configuration incorporates a spring with *α*_1_ = 0.429, *α*_2_ = 1, *k* = 20000, optimized to minimize the reaction force at slider *B*. The vertices connect at +47.7%, –33.7%, + 7.55%, and –52.86%. This design achieves a 52.86% reduction in reaction force at slider *B* and a 33.7% reduction in torque, but energy consumption increases by 47.7%, and the reaction force at hinge *A* deteriorates by 7.55%.

The radar chart clearly illustrates that optimizing one dynamic criterion in the slider-crank mechanism with a spring often comes with trade-offs in other criteria. While using a spring consistently provides improvements compared to the no-spring design, the extent of improvements and the criterion benefiting most depend on the configuration. There is no globally optimal solution for all requirements, as each design focuses on improving its primary objective (such as energy, torque, or reaction forces) while compromising others. The energy optimization design offers better balance across criteria, whereas designs focusing on torque or slider reaction forces typically achieve significant improvements in their primary objective but introduce more considerable trade-offs in other metrics. This highlights the importance of carefully considering trade-offs when selecting a design that meets specific requirements.

**Step 2**: Based on the analysis in Table 1, the objectives *R*_*Amax*_ and *N*_*Bmax*_ are converted into constraints, specifically *R*_*Amax*_≤3500 N and *N*_*Bmax*_≤750 N, as their variations have minimal impact on the overall performance of the wood splitter. Regarding reaction forces, the differences in these values do not significantly influence the design of the hinge shaft diameter, nor does the reaction force acting on slider *B* substantially affect the system’s functionality.

With this adjustment, the mathematical model is now simplified to focus on two primary objectives:

- Objectives **Φ**={Φ_1_ = *A*; Φ_2_ = *T*_*max*_}→min
- Constraints: {*R*_*Amax*_≤3500 N; *N*_*Bmax*_≤750 N}
- Design parameters: *α*_1_ = 0..1; *α*_2_ = 0..1; *k* = 0..20000 N/m

The first objective, Φ_1_ = *A*, represents the machine’s power consumption, directly related to the energy required for operation. The second objective, Φ_2_ = *T*_*max*_, is associated with motor power selection, which correlates with the initial cost of the machine. These two factors are typically the most critical considerations for consumers when evaluating machine performance and cost efficiency.

**Fig 12:**
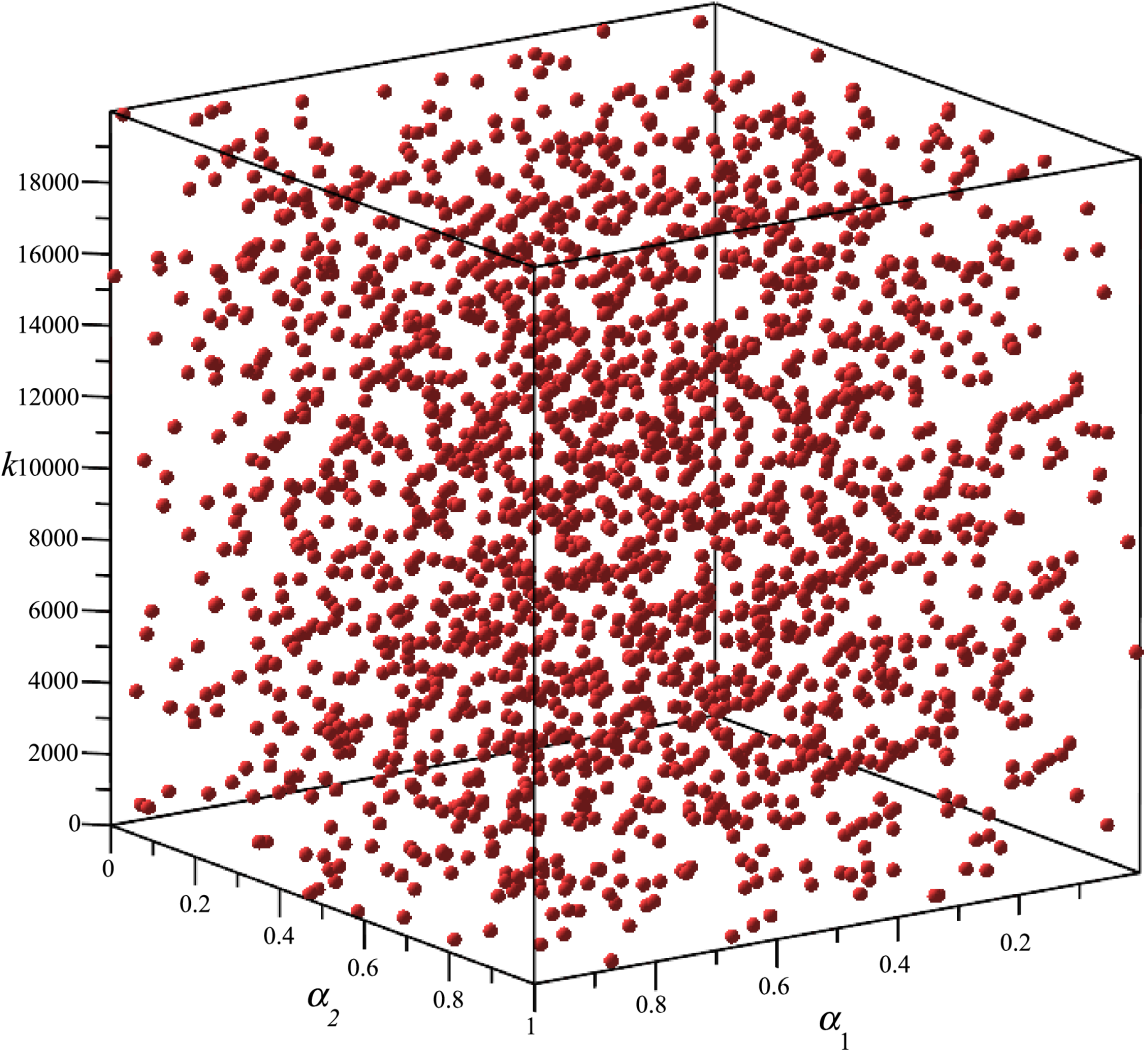
Parameter space-filling diagram by uniformly distributed series.

**Step 3**: The parameter space $𝐱={\left\{x_{1},x_{2},x_{3}\right\}}^{T}={\left\{\alpha_{1},\alpha_{2},k\right\}}^{T}$ is discretized using Sobol and Random sequences with *N* = 2^11^ = 2048 points. This ensures a uniform distribution of sampling points across the defined parameter space. The spatial distribution of these points is visually represented in Fig 12, illustrating how different sampling strategies populate the parameter domain.

**Step 4**: 2048 parameter vectors are generated for the four objective functions {*A*; *T*_*max*_; *R*_*Amax*_; *N*_*Bmax*_}. Among these, only the vectors that satisfy the constraints {*R*_*Amax*_≤3500 N; *N*_*Bmax*_≤750 N} are retained, resulting in 255 feasible parameter vectors. The symbol *A*_*i*_ represents the index of each test point vector, where *i* = 1..2048. Table 2 provides the selection values of parameter vectors from the 255 feasible solutions, while Table 3 presents a subset of the corresponding standardized vectors for these solutions.

**Table 2:** Table of parameter values of feasible solutions.

*A*_*i*_	α1	α2	*k*
*A*_3_	0.022424	0.842623	7728.166148
*A*_7_	0.412286	0.396413	12314.64138
*A*_28_	0.078606	0.819571	7174.946574
*A*_32_	0.430137	0.868572	12852.29106
*A*_36_	0.497202	0.970012	16704.19934
⁝	⁝	⁝	⁝
255 solutions			

**Table 3:** Table of objective values of feasible solutions.

*A*_*i*_	Φ_1_ = *A*	*A*_*i*_	Φ_2_ = *T*_*max*_
*A*_3_	1307.598	*A*_3_	467.895
*A*_7_	1063.753	*A*_7_	539.751
*A*_28_	1198	*A*_28_	490.33
*A*_32_	1018.2954	*A*_32_	517.16
*A*_36_	1015.385	*A*_36_	511.58
⁝	⁝	⁝	⁝
255 solutions			

Sort the values of the criteria in ascending order from the top of the two objectives (Table 4):

**Table 4:** Table of objective values of feasible solutions by increasing value from top to bottom.

*A*_*i*_	Φ_1_ = *A*	*A*_*i*_	Φ_2_ = *M*_*max*_
*A*_394_	938.0624	*A*_1464_	446.6871
*A*_1641_	944.0735	*A*_1124_	450.3182
⁝	⁝	⁝	⁝
*A*_1184_	1292.41	*A*_348_	529.509
*A*_572_	1302.202	*A*_115_	530.0144
⁝	⁝	⁝	⁝
*A*_652_	1420.97	*A*_242_	547.0937
*A*_1348_	1425.303	*A*_682_	547.2042
255 solutions			

**Step 5**: From Table 4, constraints are applied to the objective criteria based on expert recommendations:

- Φ_1_ = *A* ≤ 1300 J (energy consumption constraint)
- Φ_2_ = *T*_*max*_ ≤ 530 N.m (torque constraint)

After filtering the feasible solutions according to these conditions, 157 solutions satisfy the imposed constraints. Among them, 19 Pareto-optimal solutions are identified, representing trade-offs where no objective can be improved without worsening another. These Pareto solutions are summarized in Table 5.

**Table 5:** Set of Pareto solutions.

*A*_*i*_	α_1_	α_2_	*k*	Φ_1_ = *A*	Φ_2_ = *T*_*max*_
*A*_36_	0.4972	0.97	16704.1993	1015.3846	511.5811
*A*_51_	0.3469	0.9277	14744.7918	1184.5585	469.0856
*A*_61_	0.4191	0.8761	15664.6787	1104.1777	489.8151
*A*_264_	0.4444	0.9145	15858.378	1070.968	497.2036
*A*_310_	0.4107	0.9967	16013.2004	1120.8958	480.5664
*A*_389_	0.5003	0.9574	18783.8336	1050.7436	499.3378
*A*_612_	0.5419	0.9291	19744.4023	1008.2069	512.9468
*A*_691_	0.3205	0.9815	11585.1889	1110.8505	489.4397
*A*_693_	0.4596	0.8547	17695.1634	1093.1716	491.4834
*A*_808_	0.3083	0.8432	13691.7739	1213.5555	468.707
*A*_849_	0.5479	0.8187	19962.8862	1006.2303	516.4642
*A*_993_	0.384	0.8745	15833.5382	1162.8394	475.1062
*A*_1046_	0.4063	0.9063	15625	1120.8547	484.3182
*A*_1106_	0.2891	0.8828	13593.75	1237.7086	461.7053
*A*_1121_	0.5234	0.8984	18906.25	1021.308	510.0542
*A*_1406_	0.4941	0.9941	17695.3125	1037.8373	503.1495
*A*_1422_	0.4434	0.9355	14335.9375	1035.7565	508.2373
*A*_1644_	0.2119	0.9639	11738.2813	1271.8665	454.2354
*A*_1713_	0.5518	0.9834	18066.4063	968.3152	526.2885

**Fig 13:**
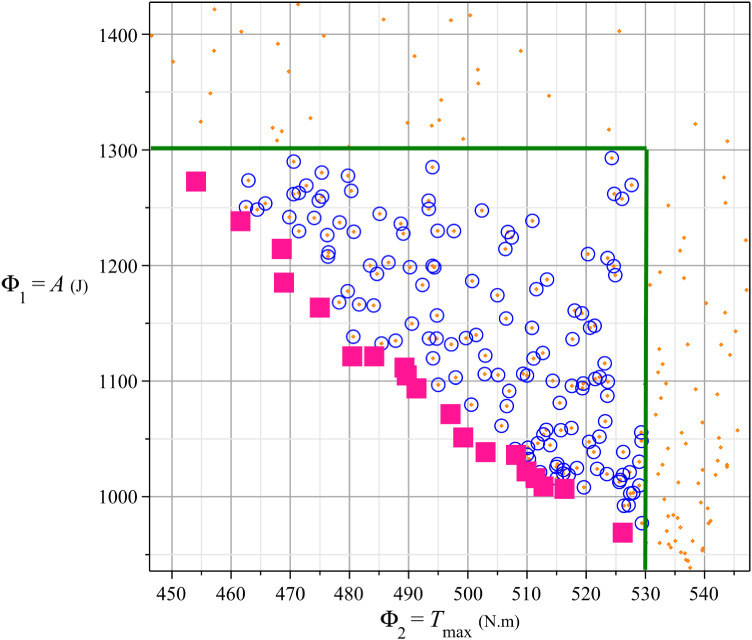
Pareto curve between 2 objectives (Φ_1_; Φ_2_).

Fig 13 provides a clear visualization of the different solution sets in the (Φ_1_; Φ_2_) plane, along with the constraints applied to the objective functions:

- Feasible solutions (255 points) are represented by orange dots, which satisfy the general constraints {*R*_*Amax*_≤3500 N; *N*_*Bmax*_≤750 N}.
- Solutions satisfying the objective conditions (157 points) are marked by blue circles surrounding the orange dots. These solutions comply with the additional constraints on the objective functions: {Φ_1_ = *A* ≤ 1300 J, Φ_2_ = *T*_*max*_ ≤ 530 N.m}
- Pareto-optimal solutions (19 points) are shown as pink squares, representing the best trade-off solutions where improving one criterion would worsen the other.

The two green lines also represent the boundary constraints Φ_1_ = 1300 J and Φ_2_ = 530 N.m. The 157 solutions satisfying the objective conditions and the 19 Pareto-optimal solutions are located below and to the left of these constraint lines, indicating that they fully comply with the imposed limits.

This hierarchical relationship between the solutions is visually evident:

- All Pareto solutions belong to the set of solutions that satisfy the objective constraints.
- All solutions satisfying the objective constraints are feasible solutions that meet the initial constraints.

The figure effectively demonstrates how filtering through constraints refines the solution space, ultimately leading to the selection of optimal design configurations.

Fig 13 illustrates the trade-off between two conflicting objectives: energy consumption (Φ_1_ = *A*) and maximum torque (Φ_2_ = *T*_*max*_). This trade-off implies that if the spring reduces energy consumption, it cannot simultaneously enhance the torque capability, and vice versa. Based on this result, experts must select the most suitable solution among the 19 Pareto-optimal solutions according to specific design requirements.

For example, if the motor power requirement is limited to 1.5 kW, corresponding to a maximum permissible torque of 477.5 N·m, the selection must be made accordingly. Referring to the table, the optimal choice is solution *A*_993_, with Energy consumption: Φ_1_ = *A* ≈ 1163 J, Maximum torque: Φ_2_ = *T*_*max*_ = 475.1 N·m. Given that one revolution takes 2 seconds, the hourly energy consumption of this configuration is 1163 J/ 2 s × 3600 s = 2,093,400 J = 0.5815 kWh.

If solution *A*_993_ is selected, the design parameters are: {*α*_1_ = 0.384; *α*_2_ = 0.8745; *k* = 15833.5382 N/m}. Comparing this result to the case without a spring (*k* = 0 for all values (*α*_1_; *α*_2_)), as shown in Table 6, we observe the following:

**Table 6:** Compare the selected optimal solution with the solution without springs.

Optimal solution	(*α*_1_; *α*_2_; *k*)	min*A*	min*T*_*max*_	min*R*_*Amax*_	min*N*_*Bmax*_
No springs	*k* = 0	**733.346**	**644.0935**	**3719.9**	**980.4651**
*A*_993_	(0.384; 0.8745; 15833.538)	**1162.8394**	**475.1062**	**3429.38**	**604.2**

- The energy consumption standard is already nearly optimal, with min*A* = 733.346 J, slightly higher than the best possible solution (Φ_1_ = *A = *732.4144 J).
- However, the torque objective is significantly worse, with *T*_*max*_ = 644.0935 N·m, making it one of the least favorable solutions.
- The constraint functions exceed the permissible limits, with values: *R*_*Amax*_ = 3719.9 N (exceeding the limit of 3500 N), *N*_*Bmax*_ = 980.4651 N (exceeding the limit of 750 N)

This comparison strongly supports the advantage of using a properly designed spring. By selecting an appropriate stiffness and installation position, as demonstrated by the 19 Pareto-optimal solutions, significant improvements can be achieved in energy efficiency and compliance with functional constraints, making the system much more effective than operating without a spring.

To validate the accuracy of the mathematical model, we compare the lead moment *T*(*Φ*) obtained from the analytical calculations with the results generated using NX software at the optimal design choice *A*_993_. The selected parameters for this verification are: *A*_993_={*α*_1_ = 0.384; *α*_2_ = 0.8745; *k* = 15833.538N/m}. Fig 18 presents the graphical comparison of *T*(*Φ*) derived from both methods. This verification step ensures that the mathematical model accurately represents the system’s behavior and aligns with numerical simulations from NX software, confirming the reliability of the proposed optimization approach.

**Fig 14:**
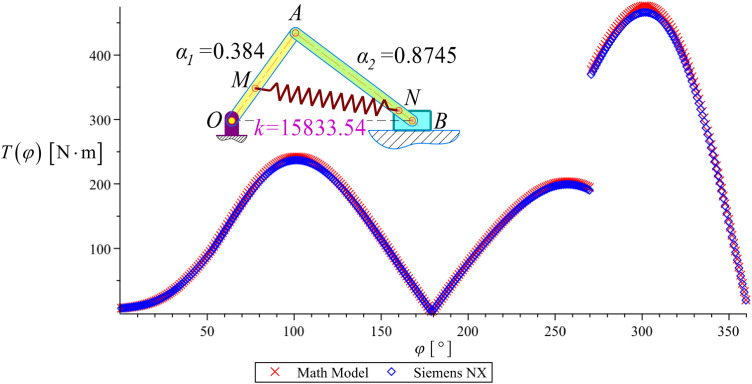
Compare the graph of the lead moment *T*(*Φ*) between the mathematical model and the NX in the optimal solution *A*_993 [_ [1]_]._

As observed in Fig 14, the results obtained from the mathematical model closely align with those from Siemens NX simulations, with a minor deviation of approximately 2%. This minimal discrepancy validates the accuracy and reliability of the proposed optimization approach. Consequently, the optimal solution identified in this study can be confidently applied to the design and construction of the wood splitter. Implementing this optimized configuration ensures the system meets the specified requirements for the two key performance criteria: **Φ**={Φ_1_ = *A*; Φ_2_ = *T*_*max*_}.

This validation further strengthens the effectiveness of combining analytical modeling with numerical simulation to optimize energy efficiency and torque performance in the wood splitter mechanism.

Due to financial and technical constraints, an experimental setup with force sensors, torque sensors, and power consumption monitoring systems has not been established. Nevertheless, the computational results from the mathematical model demonstrated less than 2% deviation when cross-validated with NX simulation software, confirming the reliability of the numerical data. Practical implementations would require additional considerations of rotational joint friction and manufacturing/assembly tolerances. This study theoretically elucidates the spring’s influence and the benefits of spring design optimization in enhancing operational efficiency and market competitiveness of slider-crank mechanisms and their derivative machinery systems.

## 5. Conclusions

This study presents a dynamic mathematical model for a spring-assisted slider-crank mechanism and introduces a CDOS-PSI hybrid optimization method to enhance the wood splitter’s primary mechanism design. This approach identified 19 Pareto-optimal solutions, providing various feasible configurations. Visualized graphs illustrating relationships between key performance criteria and spring installation positions offer more profound insights into system behavior.

The model involves complex dynamic computations with black-box objective and constraint functions (min*A*, min*T*_max_, min*R*_*Amax*_, min*N*_*Bmax*_), requiring numerous evaluations and numerical solutions of nonlinear equations per rotational cycle. On average, each function requires three seconds per parameter vector, resulting in twelve seconds when four functions are processed. GA and PSO algorithms ran for several days without converging and even caused the computer to crash. Meanwhile, using 2048 grid points, the PSI method took nearly seven hours and was completed successfully. Building on these 2048 evaluated points, the authors intend to train an equivalent ANN model for further GA or PSO trials and will present the findings in a subsequent publication.

This research’s key contribution investigates the influence of spring design variables—such as stiffness and placement—on dynamic parameters, demonstrating significant improvements in energy efficiency and mechanical load reductions. These insights provide valuable guidelines beyond wood splitters, benefiting the broader design of slider-crank mechanisms with elastic components.

Furthermore, results highlight potential cost savings in manufacturing and operational phases through strategic spring integration. Adopting this optimized design framework enables engineers to achieve enhanced system efficiency and cost-effectiveness, laying the foundational groundwork for future mechanical system advancements.

## Supporting information

S1 Data Mathematical model code, the hybrid CDOS–PSI method, and calculation data in MAPLE, along with simulation data from NX. (ZIP)
