# Supplementary material for: Multi-objective optimizing spring placement and stiffness in slider-crank mechanisms for enhanced dynamic parameters
Source: PLoS One. 2025 Sep 8;20(9):e0331341. doi: 10.1371/journal.pone.0331341 (PMC12416651; doi:10.1371/journal.pone.0331341)
Supplement: S1 Data — (ZIP) [file pone.0331341.s001.ZIP › Compare Tmax_Model&NX_(For Fig 14).pdf]

restart;

$\alpha_1 := 0.384;$

$\alpha_2 := 0.8745;$

$k := 15833.54; l_1 := 0.175; l_2 := 0.58; \omega_{OA} := 3.1416; m_1 := 41.5147; m_2 := 7.656875; m_3 := 9.8996;$
$g := 9.81; \mu := 0.3; AG_1 := 0.0019;$

$AG_2 := 0.3165732;$

$J_1 := 0.889678;$

$J_2 := 0.6204482568;$

$x_A := \varphi \to l_1 \cdot \cos(\varphi);$

$y_A := \varphi \to l_1 \cdot \sin(\varphi);$

$$x_B := \varphi \to l_1 \cdot \cos(\varphi) + \left(l_2^2 - \left(l_1 \cdot \sin(\varphi)\right)^2\right)^{\frac{1}{2}};$$

$y_B := \varphi \to 0;$

$x_{G1} := \varphi \to AG_1 \cdot \cos(\varphi);$

$y_{G1} := \varphi \to AG_1 \cdot \sin(\varphi);$

$$x_{G2} := \varphi \to x_A(\varphi) + AG_2 \cdot \sqrt{1 - \frac{l_1^2}{l_2^2} \cdot (\sin(\varphi))^2};$$

$$y_{G2} := \varphi \to \left(1 - \frac{AG_2}{l_2}\right) \cdot y_A(\varphi);$$

$x_O := \varphi \to 0;$

$y_O := \varphi \to 0;$

$$v_B := \varphi \to -\left(1 + \frac{l_1 \cdot \cos(\varphi)}{\left(l_2^2 - l_1^2 \cdot (\sin(\varphi))^2\right)^{\frac{1}{2}}}\right) \cdot \omega_{OA} \cdot l_1 \cdot \sin(\varphi);$$

$x_M := \varphi \to \alpha_1 \cdot l_1 \cdot \cos(\varphi);$

$y_M := \varphi \to \alpha_1 \cdot l_1 \cdot \sin(\varphi);$

$$x_N := \varphi \to l_1 \cdot \cos(\varphi) + \alpha_2 \cdot \sqrt{l_2^2 - l_1^2 \cdot (\sin(\varphi))^2};$$

$y_N := \varphi \to (1 - \alpha_2) \cdot l_1 \cdot \sin(\varphi);$

$l_0 := \varphi \to (1 - \alpha_1) \cdot l_1 + \alpha_2 \cdot l_2;$

$$MN := \varphi \to 10^{-10} + \sqrt{\left(x_M(\varphi) - x_N(\varphi)\right)^2 + \left(y_M(\varphi) - y_N(\varphi)\right)^2};$$

$\Delta l := \varphi \to MN(\varphi) - l_0(\varphi);$

$F_{dh} := \varphi \to k \cdot \Delta l(\varphi);$

$\varepsilon_{OA} := \varphi \to 0;$

$$\varepsilon_{AB} := \varphi \to \frac{l_2^2 - l_1^2}{\left(l_2^2 - l_1^2 \cdot (\sin(\varphi))^2\right)^{\frac{3}{2}}} \cdot \left(\omega_{OA}\right)^2 \cdot l_1 \cdot \sin(\varphi) - \frac{l_1 \cdot \cos(\varphi)}{\left(l_2^2 - l_1^2 \cdot (\sin(\varphi))^2\right)^{\frac{1}{2}}} \cdot \varepsilon_{OA}(\varphi);$$

$$a_B := \varphi \rightarrow -\varepsilon_{OA}(\varphi)\cdot l_1 \cdot \sin(\varphi)\cdot\left(1+\frac{l_1\cdot\cos(\varphi)}{\left(l_2^2 - l_1^2\cdot(\sin(\varphi))^2\right)^{\frac{1}{2}}}\right) + l_1\cdot\omega_{OA}^2 \cdot\left(\frac{l_2^2-l_1^2}{\left(l_2^2 - l_1^2\cdot(\sin(\varphi))^2\right)^{\frac{3}{2}}}\cdot l_1\cdot(\sin(\varphi))^2 - \cos(\varphi) - \frac{l_1\cdot(\cos(\varphi))^2}{\left(l_2^2 - l_1^2\cdot(\sin(\varphi))^2\right)^{\frac{1}{2}}}\right);$$

$$a_{G1x} := \varphi \rightarrow -AG_1\cdot\left(\varepsilon_{OA}(\varphi)\cdot\sin(\varphi) + \omega_{OA}^2\cdot\cos(\varphi)\right);$$

$$a_{G1y} := \varphi \rightarrow AG_1\cdot\left(\varepsilon_{OA}(\varphi)\cdot\cos(\varphi) - \omega_{OA}^2\cdot\sin(\varphi)\right);$$

$$\#a_{G2x} := \varphi \rightarrow -l_1 \sin(\varphi)\left(1+\frac{AG_2\, l_1 \cos(\varphi)}{\sqrt{1-\frac{l_1^2 \sin(\varphi)^2}{l_2^2}}\; l_2^2}\right)\varepsilon_{OA}(\varphi) - l_1\,\omega_{OA}^2\left(\cos(\varphi) + \frac{AG_2\, l_1^3 \sin(\varphi)^2\cos(\varphi)^2}{\left(1-\frac{l_1^2\sin(\varphi)^2}{l_2^2}\right)^{3/2} l_2^4} + \frac{AG_2\, l_1\cos(2\,\varphi)}{\sqrt{1-\frac{l_1^2\sin(\varphi)^2}{l_2^2}}\; l_2^2}\right);$$

$$a_{G2x} := \varphi \rightarrow -l_1\left(\varepsilon_{OA}(\varphi)\right)\sin(\varphi) - l_1\left(\omega_{OA}\right)^2\cos(\varphi) - \frac{AG_2\, l_1^4\sin(\varphi)^2\left(\omega_{OA}\right)^2\cos(\varphi)^2}{\left(1-\frac{l_1^2\sin(\varphi)^2}{l_2^2}\right)^{3/2} l_2^4} - \frac{AG_2\, l_1^2\left(\omega_{OA}\right)^2\cos(\varphi)^2}{\sqrt{1-\frac{l_1^2\sin(\varphi)^2}{l_2^2}}\; l_2^2} - \frac{AG_2\, l_1^2\sin(\varphi)\left(\varepsilon_{OA}(\varphi)\right)\cos(\varphi)}{\sqrt{1-\frac{l_1^2\sin(\varphi)^2}{l_2^2}}\; l_2^2} + \frac{AG_2\, l_1^2\sin(\varphi)^2\left(\omega_{OA}\right)^2}{\sqrt{1-\frac{l_1^2\sin(\varphi)^2}{l_2^2}}\; l_2^2};$$

$$\#a_{G2y} := \varphi \rightarrow \frac{\left(-l_2 + AG_2\right) l_1\left(\omega_{OA}^2\sin(\varphi) - \varepsilon_{OA}(\varphi)\cos(\varphi)\right)}{l_2};$$

$$a_{G2y} := \varphi \rightarrow \left(1-\frac{AG_2}{l_2}\right) l_1\left(\varepsilon_{OA}(\varphi)\right)\cos(\varphi) - \left(1-\frac{AG_2}{l_2}\right) l_1\left(\omega_{OA}\right)^2\sin(\varphi);$$

$$\alpha_1 := 0.3469213324$$

$$\alpha_2 := 0.9276630258$$

$$k := 14033.82828$$

$$l_1 := 0.175$$

$$l_2 := 0.58$$

$$\omega_{OA} := 3.1416$$

$$m_1 := 41.5147$$

$$m_2 := 7.656875$$

$$m_3 := 9.8996$$

$$g := 9.81$$

$$\mu := 0.3$$

$$AG_1 := 0.0019$$

$$AG_2 := 0.3255375$$

$$J_1 := 0.889528$$

$$J_2 := 0.34157$$

$$x_A := \varphi \mapsto l_1 \cdot \cos(\varphi)$$

$$y_A := \varphi \mapsto l_1 \cdot \sin(\varphi)$$

$$x_B := \varphi \mapsto l_1 \cdot \cos(\varphi) + \sqrt{l_2^2 - l_1^2 \cdot \sin(\varphi)^2}$$

$$y_B := \varphi \mapsto 0$$

$$x_{G1} := \varphi \mapsto AG_1 \cdot \cos(\varphi)$$

$$y_{G1} := \varphi \mapsto AG_1 \cdot \sin(\varphi)$$

$$x_{G2} := \varphi \mapsto x_A(\varphi) + AG_2 \cdot \sqrt{1 - \frac{l_1^2 \cdot \sin(\varphi)^2}{l_2^2}}$$

$$y_{G2} := \varphi \mapsto \left(1 - \frac{AG_2}{l_2}\right) \cdot y_A(\varphi)$$

$$x_{\mathrm{O}} := \varphi \mapsto 0$$

$$y_{\mathrm{O}} := \varphi \mapsto 0$$

$$v_B := \varphi \mapsto -\left(1 + \frac{l_1 \cdot \cos(\varphi)}{\sqrt{l_2^2 - l_1^2 \cdot \sin(\varphi)^2}}\right) \cdot \omega_{OA} \cdot l_1 \cdot \sin(\varphi)$$

$$x_M := \varphi \mapsto \alpha_1 \cdot l_1 \cdot \cos(\varphi)$$

$$y_M := \varphi \mapsto \alpha_1 \cdot l_1 \cdot \sin(\varphi)$$

$$x_N := \varphi \mapsto l_1 \cdot \cos(\varphi) + \alpha_2 \cdot \sqrt{l_2^2 - l_1^2 \cdot \sin(\varphi)^2}$$

$$y_N := \varphi \mapsto \left(1 - \alpha_2\right) \cdot l_1 \cdot \sin(\varphi)$$

$$l_0 := \varphi \mapsto \left(1 - \alpha_1\right) \cdot l_1 + \alpha_2 \cdot l_2$$

$$MN := \varphi \mapsto \frac{1}{10000000000} + \sqrt{\left(x_M(\varphi) - x_N(\varphi)\right)^2 + \left(y_M(\varphi) - y_N(\varphi)\right)^2}$$

$$\mathit{\Delta l} := \varphi \mapsto MN(\varphi) - l_0(\varphi)$$

$$F_{dh} := \varphi \mapsto k \cdot \mathit{\Delta l}(\varphi)$$

$$\varepsilon_{OA} := \varphi \mapsto 0$$

$$\varepsilon_{AB} := \varphi \mapsto \frac{\left(-l_1^2 + l_2^2\right) \cdot \omega_{OA}^2 \cdot l_1 \cdot \sin(\varphi)}{\left(l_2^2 - l_1^2 \cdot \sin(\varphi)^2\right)^{3/2}} - \frac{l_1 \cdot \cos(\varphi) \cdot \varepsilon_{OA}(\varphi)}{\sqrt{l_2^2 - l_1^2 \cdot \sin(\varphi)^2}}$$

$$a_B := \varphi \mapsto -\varepsilon_{OA}(\varphi) \cdot l_1 \cdot \sin(\varphi) \cdot \left(1 + \frac{l_1 \cdot \cos(\varphi)}{\sqrt{l_2^2 - l_1^2 \cdot \sin(\varphi)^2}}\right) + l_1 \cdot \omega_{OA}^2 \cdot \left(\frac{\left(-l_1^2 + l_2^2\right) \cdot l_1 \cdot \sin(\varphi)^2}{\left(l_2^2 - l_1^2 \cdot \sin(\varphi)^2\right)^{3/2}} - \cos(\varphi) - \frac{l_1 \cdot \cos(\varphi)^2}{\sqrt{l_2^2 - l_1^2 \cdot \sin(\varphi)^2}}\right)$$

$$a_{G1x} := \varphi \mapsto -AG_1 \cdot \left(\varepsilon_{OA}(\varphi) \cdot \sin(\varphi) + \omega_{OA}^2 \cdot \cos(\varphi)\right)$$

$$a_{G1y} := \varphi \mapsto AG_1 \cdot \left(\varepsilon_{OA}(\varphi) \cdot \cos(\varphi) - \omega_{OA}^2 \cdot \sin(\varphi)\right)$$

$$a_{G2x} := \varphi \mapsto -\varepsilon_{OA}(\varphi) \cdot l_1 \cdot \sin(\varphi) - l_1 \cdot \omega_{OA}^2 \cdot \cos(\varphi) - \frac{AG_2 \cdot l_1^4 \cdot \sin(\varphi)^2 \cdot \omega_{OA}^2 \cdot \cos(\varphi)^2}{\left(1 - \frac{l_1^2 \cdot \sin(\varphi)^2}{l_2^2}\right)^{3/2} \cdot l_2^4} - \frac{AG_2 \cdot l_1^2 \cdot \omega_{OA}^2 \cdot \cos(\varphi)^2}{\sqrt{1 - \frac{l_1^2 \cdot \sin(\varphi)^2}{l_2^2}} \cdot l_2^2} - \frac{AG_2 \cdot l_1^2 \cdot \sin(\varphi) \cdot \varepsilon_{OA}(\varphi) \cdot \cos(\varphi)}{\sqrt{1 - \frac{l_1^2 \cdot \sin(\varphi)^2}{l_2^2}} \cdot l_2^2} + \frac{AG_2 \cdot l_1^2 \cdot \sin(\varphi)^2 \cdot \omega_{OA}^2}{\sqrt{1 - \frac{l_1^2 \cdot \sin(\varphi)^2}{l_2^2}} \cdot l_2^2}$$

$$a_{G2y} := \varphi \mapsto \left(1 - \frac{AG_2}{l_2}\right) \cdot l_1 \cdot \varepsilon_{OA}(\varphi) \cdot \cos(\varphi) - \left(1 - \frac{AG_2}{l_2}\right) \cdot l_1 \cdot \omega_{OA}^2 \cdot \sin(\varphi) \tag{1}$$

$n := 360;$
$XX_{\mathrm{O}} := Matrix(n, 2) :$
$XX_A := Matrix(n, 2) :$
$YY_{\mathrm{O}} := Matrix(n, 2) :$
$YY_A := Matrix(n, 2) :$
$MM := Matrix(n, 2) :$
$XX_B := Matrix(n, 2) :$
$YY_B := Matrix(n, 2) :$
$NN_B := Matrix(n, 2) :$
$FF_{ms} := Matrix(n, 2) :$
$RR_{\mathrm{O}} := Matrix(n, 2) :$
$RR_A := Matrix(n, 2) :$
$RR_B := Matrix(n, 2) :$

$F := Matrix(n, 1) :$
for i **from** 271 **to** 360 **do**
$F[i] := -3233;$
od:
F

$$n := 360$$

$$\begin{bmatrix} 0 \\ 0 \\ 0 \\ 0 \\ 0 \\ 0 \\ 0 \\ 0 \\ 0 \\ 0 \\ \vdots \end{bmatrix} \quad (2)$$

360 × 1 Matrix

for i **from** 1 **by** 1 **to** n **do**

$$\varphi := \frac{(i-1)\cdot\pi}{180};$$

$$Sys\_dynamic := \Big\{$$

$$X_O + X_A + F_{dh}(\varphi)\cdot\frac{x_N(\varphi) - x_M(\varphi)}{MN(\varphi)} = m_1\cdot a_{G1x}(\varphi),$$

$$Y_O + Y_A - m_1\cdot g + F_{dh}(\varphi)\cdot\frac{y_N(\varphi) - y_M(\varphi)}{MN(\varphi)} = m_1\cdot a_{G1y}(\varphi),$$

$$M + \big(x_A(\varphi) - x_{G1}(\varphi)\big)\cdot Y_A - \big(y_A(\varphi) - y_{G1}(\varphi)\big)\cdot X_A + \big(x_O(\varphi) - x_{G1}(\varphi)\big)\cdot Y_O - \big(y_O(\varphi) - y_{G1}(\varphi)\big)\cdot X_O + \big(x_M(\varphi) - x_{G1}(\varphi)\big)\cdot F_{dh}(\varphi)\cdot\frac{y_N(\varphi) - y_M(\varphi)}{MN(\varphi)} - \big(y_M(\varphi) - y_{G1}(\varphi)\big)\cdot F_{dh}(\varphi)\cdot\frac{x_N(\varphi) - x_M(\varphi)}{MN(\varphi)} = J_1\cdot\varepsilon_{OA}(\varphi),$$

$$-X_A + X_B + F_{dh}(\varphi)\cdot\frac{x_M(\varphi) - x_N(\varphi)}{MN(\varphi)} = m_2\cdot a_{G2x}(\varphi),$$

$$-Y_A + Y_B - m_2\cdot g + F_{dh}(\varphi)\cdot\frac{y_M(\varphi) - y_N(\varphi)}{MN(\varphi)} = m_2\cdot a_{G2y}(\varphi),$$

$$\big(x_A(\varphi) - x_{G2}(\varphi)\big)\cdot(-Y_A) - \big(y_A(\varphi) - y_{G2}(\varphi)\big)\cdot(-X_A) + \big(x_B(\varphi) - x_{G2}(\varphi)\big)\cdot Y_B - \big(y_B(\varphi) - y_{G2}(\varphi)\big)\cdot X_B + \big(x_N(\varphi) - x_{G2}(\varphi)\big)\cdot F_{dh}(\varphi)\cdot\frac{y_M(\varphi) - y_N(\varphi)}{MN(\varphi)} - \big(y_N(\varphi) - y_{G2}(\varphi)\big)\cdot F_{dh}(\varphi)$$

$$\cdot\frac{x_M(\varphi) - x_N(\varphi)}{MN(\varphi)} = J_2 \cdot \varepsilon_{AB}(\varphi),$$

$$Y_B + m_3 \cdot g = N_B,$$

$$-\mu \cdot \left|N_B\right| \cdot \frac{v_B(\varphi)}{\left|v_B(\varphi)\right| + 10^{-6}} = F_{ms},$$

$$\left. -X_B + F_{ms} + F(i) = m_3 \cdot a_B(\varphi) \right\}:$$

$fsolve\left(Sys\_dynamic, \left\{X_A, Y_A, X_O, Y_O, X_B, Y_B, M, N_B, F_{ms}\right\}\right) : assign(\%);$

$XX_O(i, 1) := i; XX_A(i, 1) := i; XX_B(i, 1) = i; YY_O(i, 1) := i; YY_A(i, 1) := i; YY_B(i, 1) = i; MM(i, 1) := i; NN_B(i, 1) := i; FF_{ms}(i, 1) := i; RR_O(i, 1) := i; RR_A(i, 1) := i; RR_B(i, 1) := i;$

$XX_O(i, 2) := X_O; XX_A(i, 2) := X_A; XX_B(i, 2) = X_B; YY_O(i, 2) := Y_O; YY_A(i, 2) := Y_A; YY_B(i, 2) = Y_B; MM(i, 2) := M; NN_B(i, 2) := N_B; FF_{ms}(i, 2) := F_{ms};$

$RR_O(i, 2) := \sqrt{(X_O)^2 + (Y_O)^2};$

$RR_A(i, 2) := \sqrt{(X_A)^2 + (Y_A)^2};$

$RR_B(i, 2) := \sqrt{(X_B)^2 + (Y_B)^2};$

$unassign('X_O','X_A','X_B','Y_O','Y_A','Y_B','M','N_B','F_{ms}');$

od:

with(plots);

with(plottools);

[*animate, animate3d, animatecurve, arrow, changecoords, complexplot, complexplot3d, conformal, conformal3d, contourplot, contourplot3d, coordplot, coordplot3d, densityplot, display, dualaxisplot, fieldplot, fieldplot3d, gradplot, gradplot3d, implicitplot, implicitplot3d, inequal, interactive, interactiveparams, intersectplot, listcontplot, listcontplot3d, listdensityplot, listplot, listplot3d, loglogplot, logplot, matrixplot, multiple, odeplot, pareto, plotcompare, pointplot, pointplot3d, polarplot, polygonplot, polygonplot3d, polyhedra_supported, polyhedraplot, rootlocus, semilogplot, setcolors, setoptions, setoptions3d, shadebetween, spacecurve, sparsematrixplot, surfdata, textplot, textplot3d, tubeplot*]

[*annulus, arc, arrow, circle, cone, cuboid, curve, cutin, cutout, cylinder, disk, dodecahedron, ellipse, ellipticArc, exportplot, extrude, getdata, hemisphere, hexahedron, homothety, hyperbola, icosahedron, importplot, line, octahedron, parallelepiped, pieslice, point, polygon, polygonbyname, prism, project, rectangle, reflect, rotate, scale, sector, semitorus, sphere, stellate, tetrahedron, torus, transform, translate, triangulate*] **(3)**

(4)

File1 := *cat*(*currentdir*( ), "\\Moment_NX.xlsx");

File1 := "D:\GGDR\BAO CHI\BAO QUOC TE\Toi uu hoa su dung lo xo TQCT\Moment_NX.xlsx" **(5)**

dataM := *ExcelTools*:-*Import*(*File1*, "Sheet1", "B1:B360");

$$dataM := \begin{bmatrix} 6.41003465816000 \\ 6.70070057664000 \\ 6.99371081682000 \\ 7.29425536952000 \\ 7.60747628494000 \\ 7.93844090984000 \\ 8.29212788040000 \\ 8.67341405392000 \\ 9.08706120924000 \\ 9.53770363994000 \\ \vdots \end{bmatrix} \tag{6}$$

360 × 1 Matrix

```
AddOrder := proc(M :: Matrix, FirstOrder :: integer)
    local n, m, MO, i;
    n := LinearAlgebra:-RowDimension(M);
    m := LinearAlgebra:-ColumnDimension(M);
    MO := Matrix(n, m + 1);
    MO[ ( ) .. ( ), 2 .. m + 1 ] := M[ ( ) .. ( ), ( ) .. ( ) ];
    for i to n do
        MO[i, 1] := i + FirstOrder;
    end do;
    return MO;
end proc;
```

```
AddOrder := proc(M::Matrix, FirstOrder::integer)                    (7)
   local n, m, MO, i;
   n := LinearAlgebra:-RowDimension(M);
   m := LinearAlgebra:-ColumnDimension(M);
   MO := Matrix(n, m + 1);
   MO[ ( ) ..( ), 2 ..m + 1 ] := M[ ( ) ..( ), ( ) ..( ) ];
   for i to n do MO[i, 1] := i + FirstOrder end do;
   return MO
end proc
```

```
DM := AddOrder(dataM, 0)
```

$$DM := \begin{bmatrix} 1 & 6.41003465816000 \\ 2 & 6.70070057664000 \\ 3 & 6.99371081682000 \\ 4 & 7.29425536952000 \\ 5 & 7.60747628494000 \\ 6 & 7.93844090984000 \\ 7 & 8.29212788040000 \\ 8 & 8.67341405392000 \\ 9 & 9.08706120924000 \\ 10 & 9.53770363994000 \\ \vdots & \vdots \end{bmatrix} \quad \textbf{(8)}$$

360 × 2 Matrix

$ppp1 := pointplot(\text{abs}(MM), symbol = diagonalcross, color = red);$
$ppp2 := pointplot(DM, color = blue);$

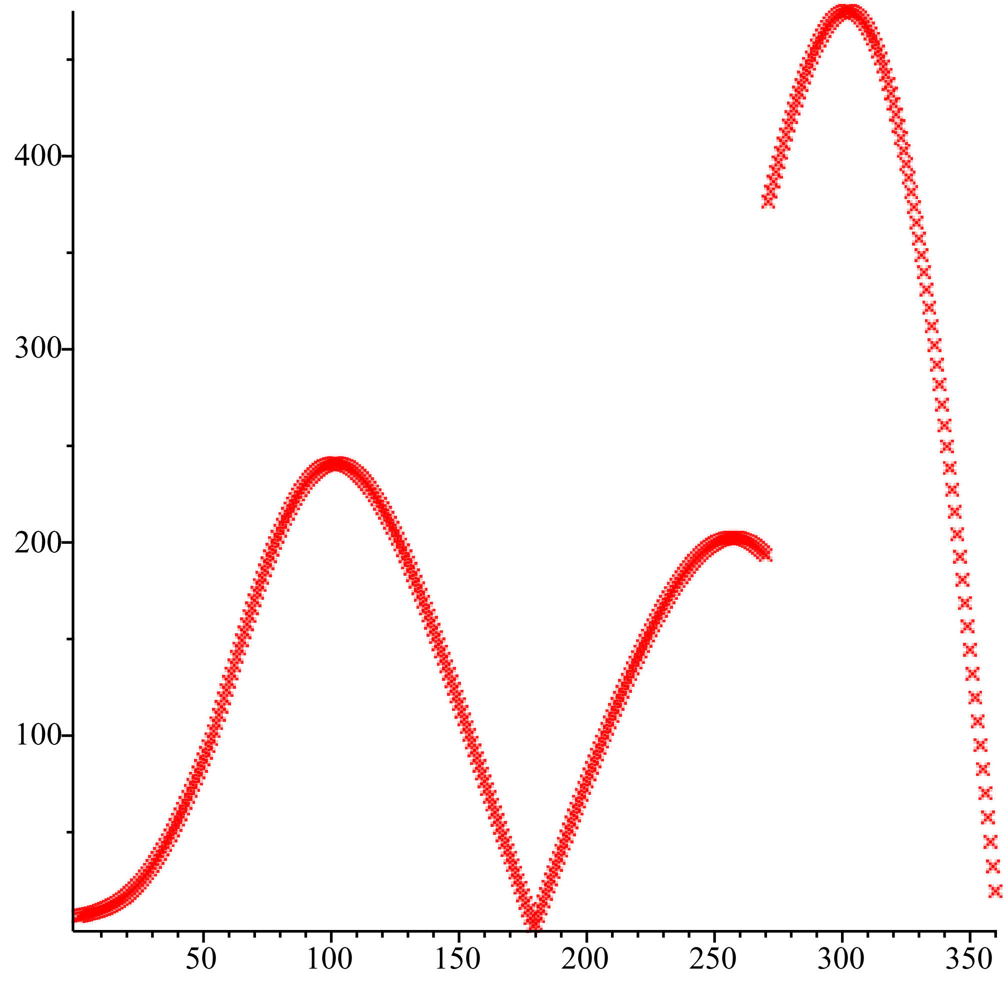

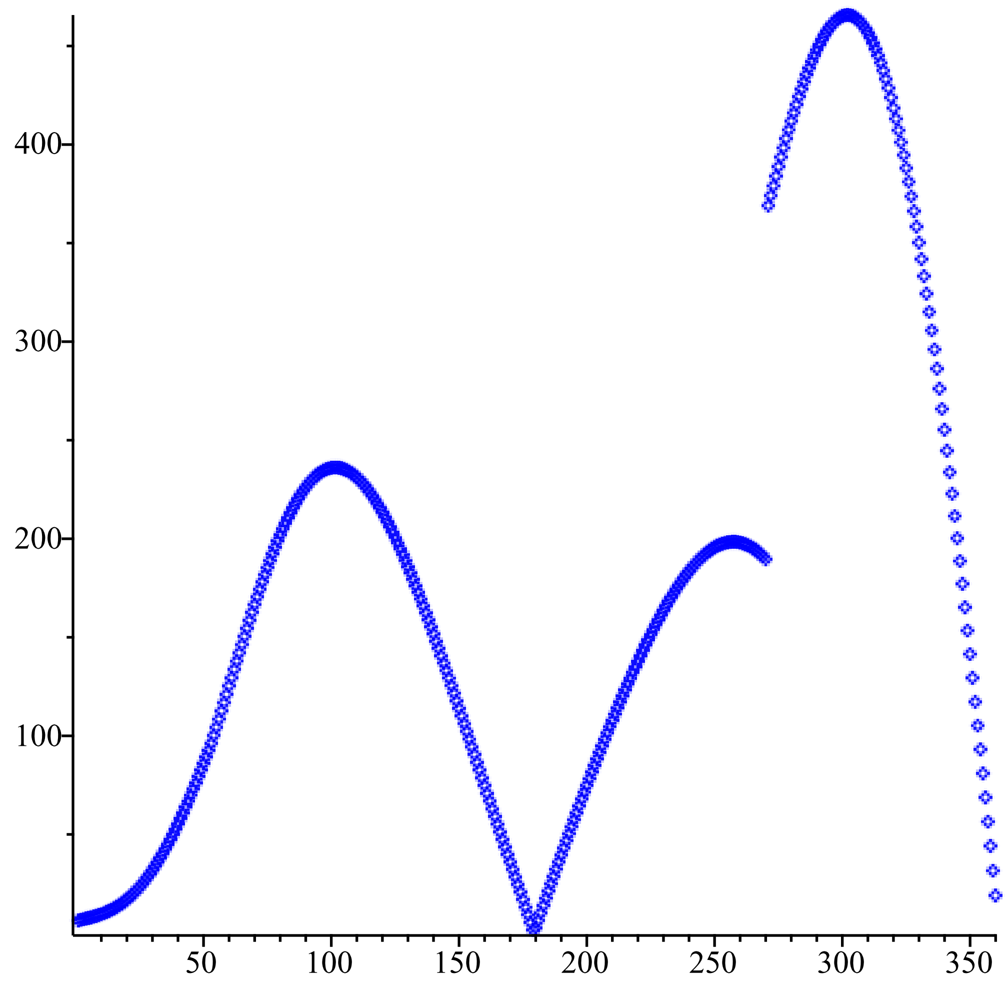

display(*ppp1*, *ppp2*)

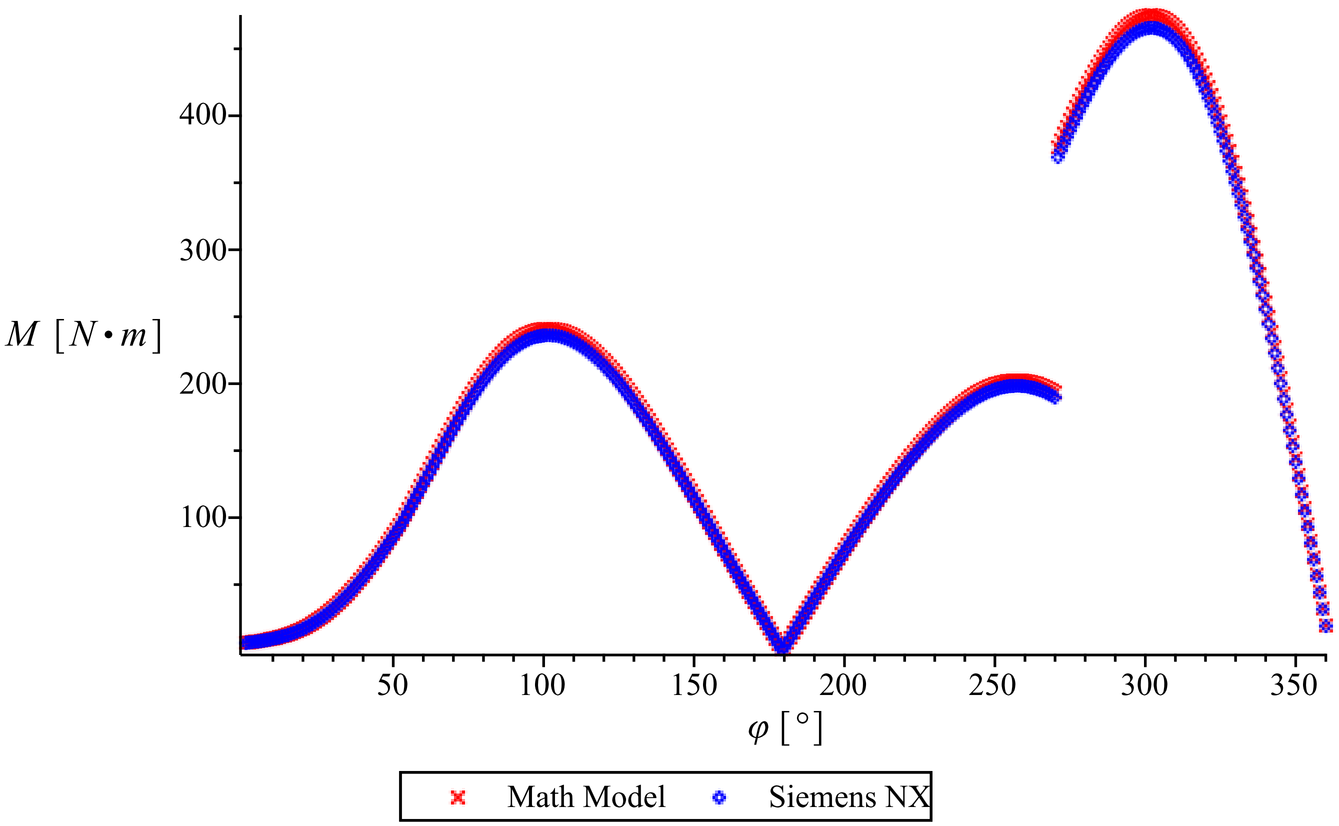

400
300
200
100
M [N•m]
50
100
150
200
250
300
350
φ [°]
Math Model
Siemens NX
