# Supplementary material for: Multi-objective optimizing spring placement and stiffness in slider-crank mechanisms for enhanced dynamic parameters
Source: PLoS One. 2025 Sep 8;20(9):e0331341. doi: 10.1371/journal.pone.0331341 (PMC12416651; doi:10.1371/journal.pone.0331341)
Supplement: S1 Data — (ZIP) [file pone.0331341.s001.ZIP › Multiobjective_Hybrid_CDOS_PSI_Stage1_(For Table 1).pdf]

$restart:$
$with(Optimization):$
$with(DirectSearch):$
$with(simplex):$
$with(linalg):$
$with(LinearAlgebra):$
$with(Student\text{:-}Calculus1):$
$with(plottools):$
$with(plots):$
$with(Statistics):$
$with(StringTools):$
$with(combinat):$
$with(Bits):$

$Dynamic\_RA := \mathbf{proc}(\alpha_1, \alpha_2, k)$

$\mathbf{local}\ \varphi, \varepsilon_{OA}, l_1, l_2, \omega_{OA}, m_1, m_2, m_3, g, \mu, F, x_A, y_A, x_B, y_B, x_{G1}, y_{G1}, x_{G2}, y_{G2}, x_O, y_O, x_M, y_M, x_N, y_N, l_0, MN, \Delta l, F_{dh}, \varepsilon_{AB}, v_B, a_B, a_{G1x}, a_{G1y}, a_{G2x}, a_{G2y}, Sys\_dynamic, X_A, Y_A, X_O, Y_O, X_B, Y_B, M, N_B, F_{ms}, n, XX_O, XX_A, XX_B, YY_O, YY_A, YY_B, MM, NN_B, FF_{ms}, i, RR_O, RR_A, RR_B, J_1, J_2, AG_2, AG_1;$

#if not is({args}, set(numeric)) **then return** ('procname')('args') **end if**;

$l_1 := 0.175; l_2 := 0.58; \omega_{OA} := 3.1416; m_1 := 41.5147; m_2 := 7.656875; m_3 := 9.8996; g := 9.81;$
$\mu := 0.3;\ AG_2 := 0.3165732; AG_1 := 0.0019;$

$J_1 := 0.889678;$

$J_2 := 0.6204482568;$

$x_A := \varphi \to l_1 \cdot \cos(\varphi);$

$y_A := \varphi \to l_1 \cdot \sin(\varphi);$

$x_B := \varphi \to l_1 \cdot \cos(\varphi) + \left(l_2^2 - (l_1 \cdot \sin(\varphi))^2\right)^{\frac{1}{2}};$

$y_B := \varphi \to 0;$

$x_{G1} := \varphi \to AG_1 \cdot \cos(\varphi);$

$y_{G1} := \varphi \to AG_1 \cdot \sin(\varphi);$

$x_{G2} := \varphi \to x_A(\varphi) + AG_2 \cdot \sqrt{1 - \frac{l_1^2}{l_2^2} \cdot (\sin(\varphi))^2};$

$y_{G2} := \varphi \to \left(1 - \frac{AG_2}{l_2}\right) \cdot y_A(\varphi);$

$x_O := \varphi \to 0;$

$y_O := \varphi \to 0;$

$v_B := \varphi \to -\left(1 + \frac{l_1 \cdot \cos(\varphi)}{\left(l_2^2 - l_1^2 \cdot (\sin(\varphi))^2\right)^{\frac{1}{2}}}\right) \cdot \omega_{OA} \cdot l_1 \cdot \sin(\varphi);$

$x_M := \varphi \to \alpha_1 \cdot l_1 \cdot \cos(\varphi);$

$y_M := \varphi \to \alpha_1 \cdot l_1 \cdot \sin(\varphi);$

$$x_N := \varphi \to l_1 \cdot \cos(\varphi) + \alpha_2 \cdot \sqrt{l_2^2 - l_1^2 \cdot (\sin(\varphi))^2};$$

$$y_N := \varphi \to \left(1 - \alpha_2\right) \cdot l_1 \cdot \sin(\varphi);$$

$$l_0 := \varphi \to \left(1 - \alpha_1\right) \cdot l_1 + \alpha_2 \cdot l_2;$$

$$MN := \varphi \to 10^{-10} + \sqrt{\left(x_M(\varphi) - x_N(\varphi)\right)^2 + \left(y_M(\varphi) - y_N(\varphi)\right)^2};$$

$$\Delta l := \varphi \to MN(\varphi) - l_0(\varphi);$$

$$F_{dh} := \varphi \to k \cdot \Delta l(\varphi);$$

$$\varepsilon_{OA} := \varphi \to 0;$$

$$\varepsilon_{AB} := \varphi \to \frac{l_2^2 - l_1^2}{\left(l_2^2 - l_1^2 \cdot (\sin(\varphi))^2\right)^{\frac{3}{2}}} \cdot \left(\omega_{OA}\right)^2 \cdot l_1 \cdot \sin(\varphi) - \frac{l_1 \cdot \cos(\varphi)}{\left(l_2^2 - l_1^2 \cdot (\sin(\varphi))^2\right)^{\frac{1}{2}}} \cdot \varepsilon_{OA}(\varphi);$$

$$a_B := \varphi \to -\varepsilon_{OA}(\varphi) \cdot l_1 \cdot \sin(\varphi) \cdot \left(1 + \frac{l_1 \cdot \cos(\varphi)}{\left(l_2^2 - l_1^2 \cdot (\sin(\varphi))^2\right)^{\frac{1}{2}}}\right) + l_1 \cdot \omega_{OA}^{\ 2}$$
$$\cdot \left(\frac{l_2^2 - l_1^2}{\left(l_2^2 - l_1^2 \cdot (\sin(\varphi))^2\right)^{\frac{3}{2}}} \cdot l_1 \cdot (\sin(\varphi))^2 - \cos(\varphi) - \frac{l_1 \cdot (\cos(\varphi))^2}{\left(l_2^2 - l_1^2 \cdot (\sin(\varphi))^2\right)^{\frac{1}{2}}}\right);$$

$$a_{G1x} := \varphi \to -AG_1 \cdot \left(\varepsilon_{OA}(\varphi) \cdot \sin(\varphi) + \omega_{OA}^{\ 2} \cdot \cos(\varphi)\right);$$

$$a_{G1y} := \varphi \to AG_1 \cdot \left(\varepsilon_{OA}(\varphi) \cdot \cos(\varphi) - \omega_{OA}^{\ 2} \cdot \sin(\varphi)\right);$$

$$\#a_{G2x} := \varphi \to -l_1 \sin(\varphi) \left(1 + \frac{AG_2\, l_1 \cos(\varphi)}{\sqrt{1 - \frac{l_1^2 \sin(\varphi)^2}{l_2^2}}\; l_2^2}\right) \varepsilon_{OA}(\varphi) - l_1\, \omega_{OA}^{\ 2} \left(\cos(\varphi) \right.$$
$$\left. + \frac{AG_2\, l_1^3 \sin(\varphi)^2 \cos(\varphi)^2}{\left(1 - \frac{l_1^2 \sin(\varphi)^2}{l_2^2}\right)^{3/2} l_2^4} + \frac{AG_2\, l_1 \cos(2\,\varphi)}{\sqrt{1 - \frac{l_1^2 \sin(\varphi)^2}{l_2^2}}\; l_2^2}\right);$$

$$a_{G2x} := \varphi \to -l_1 \left(\varepsilon_{OA}(\varphi)\right) \sin(\varphi) - l_1 \left(\omega_{OA}\right)^2 \cos(\varphi) - \frac{AG_2\, l_1^4 \sin(\varphi)^2 \left(\omega_{OA}\right)^2 \cos(\varphi)^2}{\left(1 - \frac{l_1^2 \sin(\varphi)^2}{l_2^2}\right)^{3/2} l_2^4}$$
$$- \frac{AG_2\, l_1^2 \left(\omega_{OA}\right)^2 \cos(\varphi)^2}{\sqrt{1 - \frac{l_1^2 \sin(\varphi)^2}{l_2^2}}\; l_2^2} - \frac{AG_2\, l_1^2 \sin(\varphi) \left(\varepsilon_{OA}(\varphi)\right) \cos(\varphi)}{\sqrt{1 - \frac{l_1^2 \sin(\varphi)^2}{l_2^2}}\; l_2^2} + \frac{AG_2\, l_1^2 \sin(\varphi)^2 \left(\omega_{OA}\right)^2}{\sqrt{1 - \frac{l_1^2 \sin(\varphi)^2}{l_2^2}}\; l_2^2};$$

$$\#a_{G2y} := \varphi \to \frac{\left(-l_2 + AG_2\right) l_1 \left(\omega_{OA}^{\ 2} \sin(\varphi) - \varepsilon_{OA}(\varphi) \cos(\varphi)\right)}{l_2};$$

$$a_{G2y} := \varphi \to \left(1 - \frac{AG_2}{l_2}\right) l_1 \left(\varepsilon_{OA}(\varphi)\right) \cos(\varphi) - \left(1 - \frac{AG_2}{l_2}\right) l_1 \left(\omega_{OA}\right)^2 \sin(\varphi);$$

$n := 360;$
$XX_{O} := Matrix(n, 2):$
$XX_{A} := Matrix(n, 2):$
$YY_{O} := Matrix(n, 2):$
$YY_{A} := Matrix(n, 2):$
$MM := Matrix(n, 2):$
$XX_{B} := Matrix(n, 2):$
$YY_{B} := Matrix(n, 2):$
$NN_{B} := Matrix(n, 2):$
$FF_{ms} := Matrix(n, 2):$
$RR_{O} := Matrix(n, 2):$
$RR_{A} := Matrix(n, 2):$
$RR_{B} := Matrix(n, 2):$
$F := Matrix(n, 1):$
for i **from** 271 **to** 360 **do**
$F[i] := -3233;$
od:
for i **from** 1 **by** 1 **to** n **do**

$$\varphi := \frac{(i-1)\cdot\pi}{180};$$

$$Sys\_dynamic := \Bigg\{$$

$$X_{O} + X_{A} + F_{dh}(\varphi)\cdot\frac{x_{N}(\varphi) - x_{M}(\varphi)}{MN(\varphi)} = m_{1}\cdot a_{G1x}(\varphi),$$

$$Y_{O} + Y_{A} - m_{1}\cdot g + F_{dh}(\varphi)\cdot\frac{y_{N}(\varphi) - y_{M}(\varphi)}{MN(\varphi)} = m_{1}\cdot a_{G1y}(\varphi),$$

$$M + \left(x_{A}(\varphi) - x_{G1}(\varphi)\right)\cdot Y_{A} - \left(y_{A}(\varphi) - y_{G1}(\varphi)\right)\cdot X_{A} + \left(x_{O}(\varphi) - x_{G1}(\varphi)\right)\cdot Y_{O} - \left(y_{O}(\varphi) - y_{G1}(\varphi)\right)\cdot X_{O} + \left(x_{M}(\varphi) - x_{G1}(\varphi)\right)\cdot F_{dh}(\varphi)\cdot\frac{y_{N}(\varphi) - y_{M}(\varphi)}{MN(\varphi)} - \left(y_{M}(\varphi) - y_{G1}(\varphi)\right)\cdot F_{dh}(\varphi)\cdot\frac{x_{N}(\varphi) - x_{M}(\varphi)}{MN(\varphi)} = J_{1}\cdot\varepsilon_{OA}(\varphi),$$

$$-X_{A} + X_{B} + F_{dh}(\varphi)\cdot\frac{x_{M}(\varphi) - x_{N}(\varphi)}{MN(\varphi)} = m_{2}\cdot a_{G2x}(\varphi),$$

$$-Y_{A} + Y_{B} - m_{2}\cdot g + F_{dh}(\varphi)\cdot\frac{y_{M}(\varphi) - y_{N}(\varphi)}{MN(\varphi)} = m_{2}\cdot a_{G2y}(\varphi),$$

$$\left(x_{A}(\varphi) - x_{G2}(\varphi)\right)\cdot(-Y_{A}) - \left(y_{A}(\varphi) - y_{G2}(\varphi)\right)\cdot(-X_{A}) + \left(x_{B}(\varphi) - x_{G2}(\varphi)\right)\cdot Y_{B} - \left(y_{B}(\varphi) - y_{G2}(\varphi)\right)\cdot X_{B} + \left(x_{N}(\varphi) - x_{G2}(\varphi)\right)\cdot F_{dh}(\varphi)\cdot\frac{y_{M}(\varphi) - y_{N}(\varphi)}{MN(\varphi)} - \left(y_{N}(\varphi) - y_{G2}(\varphi)\right)\cdot F_{dh}(\varphi)\cdot\frac{x_{M}(\varphi) - x_{N}(\varphi)}{MN(\varphi)} = J_{2}\cdot\varepsilon_{AB}(\varphi),$$

$Y_B + m_3 \cdot g = N_B,$

$-\mu \cdot |N_B| \cdot \dfrac{v_B(\varphi)}{|v_B(\varphi)| + 10^{-6}} = F_{ms},$

$-X_B + F_{ms} + F(i) = m_3 \cdot a_B(\varphi) \}:$

$fsolve(Sys\_dynamic, \{X_A, Y_A, X_O, Y_O, X_B, Y_B, M, N_B, F_{ms}\}) : assign(\%);$

$XX_O(i,1) := i; XX_A(i,1) := i; XX_B(i,1) = i; YY_O(i,1) := i; YY_A(i,1) := i; YY_B(i,1) = i; MM(i,1) := i; NN_B(i,1) := i; FF_{ms}(i,1) := i; RR_O(i,1) := i; RR_A(i,1) := i; RR_B(i,1) := i;$

$XX_O(i,2) := X_O; XX_A(i,2) := X_A; XX_B(i,2) = X_B; YY_O(i,2) := Y_O; YY_A(i,2) := Y_A; YY_B(i,2) = Y_B; MM(i,2) := M; NN_B(i,2) := N_B; FF_{ms}(i,2) := F_{ms};$

$RR_O(i,2) := \sqrt{(X_O)^2 + (Y_O)^2};$

$RR_A(i,2) := \sqrt{(X_A)^2 + (Y_A)^2};$

$RR_B(i,2) := \sqrt{(X_B)^2 + (Y_B)^2};$

$unassign('X_O','X_A','X_B','Y_O','Y_A','Y_B','M','N_B','F_{ms}');$

od:

return $\max(|RR_A|);$

end proc:

$DirectSearch\text{:-}Search\left(Dynamic\_RA, \{\alpha_1 = 0..1, \alpha_2 = 0..1, k = 0..20000\}, checkexit = 10, tolerances = 10\text{^}(-14), evaluationlimit = 30000\right);$

$$\left[3231.70788494310, \begin{bmatrix} 0.540686104964524 \\ 0.999999649801343 \\ 19998.4334410834 \end{bmatrix}, 1873\right] \quad \textbf{(1)}$$

$Dynamic\_NB :=$ **proc** (α_1, α_2, k)

local $\varphi, \varepsilon_{OA}, l_1, l_2, \omega_{OA}, m_1, m_2, m_3, g, \mu, F, x_A, y_A, x_B, y_B, x_{G1}, y_{G1}, x_{G2}, y_{G2}, x_O, y_O, x_M, y_M, x_N, y_N, l_0, MN, \Delta l, F_{dh}, \varepsilon_{AB}, v_B, a_B, a_{G1x}, a_{G1y}, a_{G2x}, a_{G2y}, Sys\_dynamic, X_A, Y_A, X_O, Y_O, X_B, Y_B, M, N_B, F_{ms}, n, XX_O, XX_A, XX_B, YY_O, YY_A, YY_B, MM, NN_B, FF_{ms}, i, RR_O, RR_A, RR_B, J_1, J_2, AG_2, AG_1;$

#if not is({args}, set(numeric)) **then return** ('procname')('args') **end if**;

$l_1 := 0.175; l_2 := 0.58; \omega_{OA} := 3.1416; m_1 := 41.5147; m_2 := 7.656875; m_3 := 9.8996; g := 9.81;$
$\mu := 0.3; AG_2 := 0.3165732; AG_1 := 0.0019;$

$J_1 := 0.889678;$

$J_2 := 0.6204482568;$

$x_A := \varphi \rightarrow l_1 \cdot \cos(\varphi);$

$y_A := \varphi \rightarrow l_1 \cdot \sin(\varphi);$

$x_B := \varphi \rightarrow l_1 \cdot \cos(\varphi) + \left(l_2^2 - (l_1 \cdot \sin(\varphi))^2\right)^{\frac{1}{2}};$

$y_B := \varphi \to 0;$

$x_{G1} := \varphi \to AG_1 \cdot \cos(\varphi);$

$y_{G1} := \varphi \to AG_1 \cdot \sin(\varphi);$

$$x_{G2} := \varphi \to x_A(\varphi) + AG_2 \cdot \sqrt{1 - \frac{l_1^2}{l_2^2} \cdot (\sin(\varphi))^2};$$

$$y_{G2} := \varphi \to \left(1 - \frac{AG_2}{l_2}\right) \cdot y_A(\varphi);$$

$x_O := \varphi \to 0;$

$y_O := \varphi \to 0;$

$$v_B := \varphi \to -\left(1 + \frac{l_1 \cdot \cos(\varphi)}{\left(l_2^2 - l_1^2 \cdot (\sin(\varphi))^2\right)^{\frac{1}{2}}}\right) \cdot \omega_{OA} \cdot l_1 \cdot \sin(\varphi);$$

$x_M := \varphi \to \alpha_1 \cdot l_1 \cdot \cos(\varphi);$

$y_M := \varphi \to \alpha_1 \cdot l_1 \cdot \sin(\varphi);$

$x_N := \varphi \to l_1 \cdot \cos(\varphi) + \alpha_2 \cdot \sqrt{l_2^2 - l_1^2 \cdot (\sin(\varphi))^2};$

$y_N := \varphi \to (1 - \alpha_2) \cdot l_1 \cdot \sin(\varphi);$

$l_0 := \varphi \to (1 - \alpha_1) \cdot l_1 + \alpha_2 \cdot l_2;$

$MN := \varphi \to 10^{-10} + \sqrt{(x_M(\varphi) - x_N(\varphi))^2 + (y_M(\varphi) - y_N(\varphi))^2};$

$\Delta l := \varphi \to MN(\varphi) - l_0(\varphi);$

$F_{dh} := \varphi \to k \cdot \Delta l(\varphi);$

$\varepsilon_{OA} := \varphi \to 0;$

$$\varepsilon_{AB} := \varphi \to \frac{l_2^2 - l_1^2}{\left(l_2^2 - l_1^2 \cdot (\sin(\varphi))^2\right)^{\frac{3}{2}}} \cdot (\omega_{OA})^2 \cdot l_1 \cdot \sin(\varphi) - \frac{l_1 \cdot \cos(\varphi)}{\left(l_2^2 - l_1^2 \cdot (\sin(\varphi))^2\right)^{\frac{1}{2}}} \cdot \varepsilon_{OA}(\varphi);$$

$$a_B := \varphi \to -\varepsilon_{OA}(\varphi) \cdot l_1 \cdot \sin(\varphi) \cdot \left(1 + \frac{l_1 \cdot \cos(\varphi)}{\left(l_2^2 - l_1^2 \cdot (\sin(\varphi))^2\right)^{\frac{1}{2}}}\right) + l_1 \cdot \omega_{OA}^2$$
$$\cdot \left(\frac{l_2^2 - l_1^2}{\left(l_2^2 - l_1^2 \cdot (\sin(\varphi))^2\right)^{\frac{3}{2}}} \cdot l_1 \cdot (\sin(\varphi))^2 - \cos(\varphi) - \frac{l_1 \cdot (\cos(\varphi))^2}{\left(l_2^2 - l_1^2 \cdot (\sin(\varphi))^2\right)^{\frac{1}{2}}}\right);$$

$a_{G1x} := \varphi \to -AG_1 \cdot \left(\varepsilon_{OA}(\varphi) \cdot \sin(\varphi) + \omega_{OA}^2 \cdot \cos(\varphi)\right);$

$a_{G1y} := \varphi \to AG_1 \cdot \left(\varepsilon_{OA}(\varphi) \cdot \cos(\varphi) - \omega_{OA}^2 \cdot \sin(\varphi)\right);$

$$\#a_{G2x} := \varphi \to -l_1 \sin(\varphi) \left(1 + \frac{AG_2\, l_1 \cos(\varphi)}{\sqrt{1 - \frac{l_1^2 \sin(\varphi)^2}{l_2^2}}\; l_2^2}\right) \varepsilon_{OA}(\varphi) - l_1\, \omega_{OA}^2 \left(\cos(\varphi)\right.$$

$$+\frac{AG_2\, l_1^3 \sin(\varphi)^2 \cos(\varphi)^2}{\left(1-\frac{l_1^2 \sin(\varphi)^2}{l_2^2}\right)^{3/2} l_2^4}+\frac{AG_2\, l_1 \cos(2\varphi)}{\sqrt{1-\frac{l_1^2 \sin(\varphi)^2}{l_2^2}}\; l_2^2}\Bigg);$$

$$a_{G2x} := \varphi \to -l_1 \left(\varepsilon_{OA}(\varphi)\right) \sin(\varphi) - l_1 \left(\omega_{OA}\right)^2 \cos(\varphi) - \frac{AG_2\, l_1^4 \sin(\varphi)^2 \left(\omega_{OA}\right)^2 \cos(\varphi)^2}{\left(1-\frac{l_1^2 \sin(\varphi)^2}{l_2^2}\right)^{3/2} l_2^4}$$

$$-\frac{AG_2\, l_1^2 \left(\omega_{OA}\right)^2 \cos(\varphi)^2}{\sqrt{1-\frac{l_1^2 \sin(\varphi)^2}{l_2^2}}\; l_2^2}-\frac{AG_2\, l_1^2 \sin(\varphi) \left(\varepsilon_{OA}(\varphi)\right) \cos(\varphi)}{\sqrt{1-\frac{l_1^2 \sin(\varphi)^2}{l_2^2}}\; l_2^2}+\frac{AG_2\, l_1^2 \sin(\varphi)^2 \left(\omega_{OA}\right)^2}{\sqrt{1-\frac{l_1^2 \sin(\varphi)^2}{l_2^2}}\; l_2^2};$$

$$\#a_{G2y} := \varphi \to \frac{\left(-l_2 + AG_2\right) l_1 \left(\omega_{OA}^{\;2} \sin(\varphi) - \varepsilon_{OA}(\varphi)\cos(\varphi)\right)}{l_2};$$

$$a_{G2y} := \varphi \to \left(1-\frac{AG_2}{l_2}\right) l_1 \left(\varepsilon_{OA}(\varphi)\right) \cos(\varphi) - \left(1-\frac{AG_2}{l_2}\right) l_1 \left(\omega_{OA}\right)^2 \sin(\varphi);$$

$n := 360;$
$XX_O := Matrix(n, 2)$:
$XX_A := Matrix(n, 2)$:
$YY_O := Matrix(n, 2)$:
$YY_A := Matrix(n, 2)$:
$MM := Matrix(n, 2)$:
$XX_B := Matrix(n, 2)$:
$YY_B := Matrix(n, 2)$:
$NN_B := Matrix(n, 2)$:
$FF_{ms} := Matrix(n, 2)$:
$RR_O := Matrix(n, 2)$:
$RR_A := Matrix(n, 2)$:
$RR_B := Matrix(n, 2)$:
$F := Matrix(n, 1)$:
for i **from** 271 **to** 360 **do**
$F[i] := -3233;$
od:
for i **from** 1 **by** 1 **to** n **do**

$$\varphi := \frac{(i-1)\cdot\pi}{180};$$

$Sys\_dynamic := \Big\{$

$$X_O + X_A + F_{dh}(\varphi)\cdot\frac{x_N(\varphi) - x_M(\varphi)}{MN(\varphi)} = m_1\cdot a_{G1x}(\varphi),$$

$$Y_O + Y_A - m_1\cdot g + F_{dh}(\varphi)\cdot\frac{y_N(\varphi) - y_M(\varphi)}{MN(\varphi)} = m_1\cdot a_{G1y}(\varphi),$$

$$M + \left(x_A(\varphi) - x_{G1}(\varphi)\right)\cdot Y_A - \left(y_A(\varphi) - y_{G1}(\varphi)\right)\cdot X_A + \left(x_O(\varphi) - x_{G1}(\varphi)\right)\cdot Y_O - \left(y_O(\varphi) - y_{G1}(\varphi)\right)$$
$$\cdot X_O + \left(x_M(\varphi) - x_{G1}(\varphi)\right)\cdot F_{dh}(\varphi)\cdot\frac{y_N(\varphi) - y_M(\varphi)}{MN(\varphi)} - \left(y_M(\varphi) - y_{G1}(\varphi)\right)\cdot F_{dh}(\varphi)$$
$$\cdot\frac{x_N(\varphi) - x_M(\varphi)}{MN(\varphi)} = J_1\cdot\varepsilon_{OA}(\varphi),$$

$$-X_A + X_B + F_{dh}(\varphi)\cdot\frac{x_M(\varphi) - x_N(\varphi)}{MN(\varphi)} = m_2\cdot a_{G2x}(\varphi),$$

$$-Y_A + Y_B - m_2\cdot g + F_{dh}(\varphi)\cdot\frac{y_M(\varphi) - y_N(\varphi)}{MN(\varphi)} = m_2\cdot a_{G2y}(\varphi),$$

$$\left(x_A(\varphi) - x_{G2}(\varphi)\right)\cdot\left(-Y_A\right) - \left(y_A(\varphi) - y_{G2}(\varphi)\right)\cdot\left(-X_A\right) + \left(x_B(\varphi) - x_{G2}(\varphi)\right)\cdot Y_B - \left(y_B(\varphi)\right.$$
$$\left. - y_{G2}(\varphi)\right)\cdot X_B + \left(x_N(\varphi) - x_{G2}(\varphi)\right)\cdot F_{dh}(\varphi)\cdot\frac{y_M(\varphi) - y_N(\varphi)}{MN(\varphi)} - \left(y_N(\varphi) - y_{G2}(\varphi)\right)\cdot F_{dh}(\varphi)$$
$$\cdot\frac{x_M(\varphi) - x_N(\varphi)}{MN(\varphi)} = J_2\cdot\varepsilon_{AB}(\varphi),$$

$$Y_B + m_3\cdot g = N_B,$$

$$-\mu\cdot|N_B|\cdot\frac{v_B(\varphi)}{|v_B(\varphi)| + 10^{-6}} = F_{ms},$$

$$\left.-X_B + F_{ms} + F(i) = m_3\cdot a_B(\varphi)\right\}:$$

$fsolve\left(Sys\_dynamic, \{X_A, Y_A, X_O, Y_O, X_B, Y_B, M, N_B, F_{ms}\}\right) : assign(\%);$

$XX_O(i, 1) := i; XX_A(i, 1) := i; XX_B(i, 1) = i; YY_O(i, 1) := i; YY_A(i, 1) := i; YY_B(i, 1) = i; MM(i, 1) := i; NN_B(i, 1) := i; FF_{ms}(i, 1) := i; RR_O(i, 1) := i; RR_A(i, 1) := i; RR_B(i, 1) := i;$

$XX_O(i, 2) := X_O; XX_A(i, 2) := X_A; XX_B(i, 2) = X_B; YY_O(i, 2) := Y_O; YY_A(i, 2) := Y_A; YY_B(i, 2) = Y_B; MM(i, 2) := M; NN_B(i, 2) := N_B; FF_{ms}(i, 2) := F_{ms};$

$RR_O(i, 2) := \sqrt{(X_O)^2 + (Y_O)^2};$

$RR_A(i, 2) := \sqrt{(X_A)^2 + (Y_A)^2};$

$RR_B(i, 2) := \sqrt{(X_B)^2 + (Y_B)^2};$

$unassign('X_O','X_A','X_B','Y_O','Y_A','Y_B','M','N_B','F_{ms}');$

od:

return $\max(|NN_B|);$

end proc:

$DirectSearch\text{:-}Search\left(Dynamic\_NB, \{\alpha_1 = 0..1, \alpha_2 = 0..1, k = 0..20000\}, checkexit = 10, tolerances = 10^{(-14)}, evaluationlimit = 30000\right);$

$$\left[570.537594914606, \begin{bmatrix} 0.429089123492961 \\ 0.999999999999943 \\ 19999.9999999939 \end{bmatrix}, 1881\right] \quad \textbf{(2)}$$

$Dynamic\_E :=$ **proc**(α_1, α_2, k)

local $\varphi, \varepsilon_{OA}, l_1, l_2, \omega_{OA}, m_1, m_2, m_3, g, \mu, F, x_A, y_A, x_B, y_B, x_{G1}, y_{G1}, x_{G2}, y_{G2}, x_O, y_O, x_M, y_M, x_N, y_N, l_0, MN, \Delta l, F_{dh}, \varepsilon_{AB}, v_B, a_B, a_{G1x}, a_{G1y}, a_{G2x}, a_{G2y}, Sys\_dynamic, X_A, Y_A, X_O, Y_O, X_B, Y_B, M, N_B, F_{ms}, n, XX_O, XX_A, XX_B, YY_O, YY_A, YY_B, MM, NN_B, FF_{ms}, i, RR_O, RR_A, RR_B, J_1, J_2, AG_2, AG_1$;

#if not is({args}, set(numeric)) **then return** *('procname')('args')* **end if**;

$l_1 := 0.175; l_2 := 0.58; \omega_{OA} := 3.1416; m_1 := 41.5147; m_2 := 7.656875; m_3 := 9.8996; g := 9.81;$
$\mu := 0.3;\ AG_2 := 0.3165732; AG_1 := 0.0019;$

$J_1 := 0.889678;$

$J_2 := 0.6204482568;$

$x_A := \varphi \to l_1 \cdot \cos(\varphi);$

$y_A := \varphi \to l_1 \cdot \sin(\varphi);$

$$x_B := \varphi \to l_1 \cdot \cos(\varphi) + \left(l_2^2 - (l_1 \cdot \sin(\varphi))^2\right)^{\frac{1}{2}};$$

$y_B := \varphi \to 0;$

$x_{G1} := \varphi \to AG_1 \cdot \cos(\varphi);$

$y_{G1} := \varphi \to AG_1 \cdot \sin(\varphi);$

$$x_{G2} := \varphi \to x_A(\varphi) + AG_2 \cdot \sqrt{1 - \frac{l_1^2}{l_2^2} \cdot (\sin(\varphi))^2};$$

$$y_{G2} := \varphi \to \left(1 - \frac{AG_2}{l_2}\right) \cdot y_A(\varphi);$$

$x_O := \varphi \to 0;$

$y_O := \varphi \to 0;$

$$v_B := \varphi \to -\left(1 + \frac{l_1 \cdot \cos(\varphi)}{\left(l_2^2 - l_1^2 \cdot (\sin(\varphi))^2\right)^{\frac{1}{2}}}\right) \cdot \omega_{OA} \cdot l_1 \cdot \sin(\varphi);$$

$x_M := \varphi \to \alpha_1 \cdot l_1 \cdot \cos(\varphi);$

$y_M := \varphi \to \alpha_1 \cdot l_1 \cdot \sin(\varphi);$

$$x_N := \varphi \to l_1 \cdot \cos(\varphi) + \alpha_2 \cdot \sqrt{l_2^2 - l_1^2 \cdot (\sin(\varphi))^2};$$

$y_N := \varphi \to (1 - \alpha_2) \cdot l_1 \cdot \sin(\varphi);$

$l_0 := \varphi \to (1 - \alpha_1) \cdot l_1 + \alpha_2 \cdot l_2;$

$$MN := \varphi \to 10^{-10} + \sqrt{(x_M(\varphi) - x_N(\varphi))^2 + (y_M(\varphi) - y_N(\varphi))^2};$$

$\Delta l := \varphi \to MN(\varphi) - l_0(\varphi);$

$F_{dh} := \varphi \to k \cdot \Delta l(\varphi);$

$$\varepsilon_{OA} := \varphi \to 0;$$

$$\varepsilon_{AB} := \varphi \to \frac{l_2^2 - l_1^2}{\left(l_2^2 - l_1^2 \cdot (\sin(\varphi))^2\right)^{\frac{3}{2}}} \cdot \left(\omega_{OA}\right)^2 \cdot l_1 \cdot \sin(\varphi) - \frac{l_1 \cdot \cos(\varphi)}{\left(l_2^2 - l_1^2 \cdot (\sin(\varphi))^2\right)^{\frac{1}{2}}} \cdot \varepsilon_{OA}(\varphi);$$

$$a_B := \varphi \to -\varepsilon_{OA}(\varphi) \cdot l_1 \cdot \sin(\varphi) \cdot \left(1 + \frac{l_1 \cdot \cos(\varphi)}{\left(l_2^2 - l_1^2 \cdot (\sin(\varphi))^2\right)^{\frac{1}{2}}}\right) + l_1 \cdot \omega_{OA}^{\;2} \cdot \left(\frac{l_2^2 - l_1^2}{\left(l_2^2 - l_1^2 \cdot (\sin(\varphi))^2\right)^{\frac{3}{2}}} \cdot l_1 \cdot (\sin(\varphi))^2 - \cos(\varphi) - \frac{l_1 \cdot (\cos(\varphi))^2}{\left(l_2^2 - l_1^2 \cdot (\sin(\varphi))^2\right)^{\frac{1}{2}}}\right);$$

$$a_{G1x} := \varphi \to -AG_1 \cdot \left(\varepsilon_{OA}(\varphi) \cdot \sin(\varphi) + \omega_{OA}^{\;2} \cdot \cos(\varphi)\right);$$

$$a_{G1y} := \varphi \to AG_1 \cdot \left(\varepsilon_{OA}(\varphi) \cdot \cos(\varphi) - \omega_{OA}^{\;2} \cdot \sin(\varphi)\right);$$

$$\#a_{G2x} := \varphi \to -l_1 \sin(\varphi) \left(1 + \frac{AG_2\, l_1 \cos(\varphi)}{\sqrt{1 - \frac{l_1^2 \sin(\varphi)^2}{l_2^2}}\; l_2^2}\right) \varepsilon_{OA}(\varphi) - l_1\, \omega_{OA}^{\;2} \left(\cos(\varphi) + \frac{AG_2\, l_1^3 \sin(\varphi)^2 \cos(\varphi)^2}{\left(1 - \frac{l_1^2 \sin(\varphi)^2}{l_2^2}\right)^{3/2} l_2^4} + \frac{AG_2\, l_1 \cos(2\,\varphi)}{\sqrt{1 - \frac{l_1^2 \sin(\varphi)^2}{l_2^2}}\; l_2^2}\right);$$

$$a_{G2x} := \varphi \to -l_1 \left(\varepsilon_{OA}(\varphi)\right) \sin(\varphi) - l_1 \left(\omega_{OA}\right)^2 \cos(\varphi) - \frac{AG_2\, l_1^4 \sin(\varphi)^2 \left(\omega_{OA}\right)^2 \cos(\varphi)^2}{\left(1 - \frac{l_1^2 \sin(\varphi)^2}{l_2^2}\right)^{3/2} l_2^4} - \frac{AG_2\, l_1^2 \left(\omega_{OA}\right)^2 \cos(\varphi)^2}{\sqrt{1 - \frac{l_1^2 \sin(\varphi)^2}{l_2^2}}\; l_2^2} - \frac{AG_2\, l_1^2 \sin(\varphi) \left(\varepsilon_{OA}(\varphi)\right) \cos(\varphi)}{\sqrt{1 - \frac{l_1^2 \sin(\varphi)^2}{l_2^2}}\; l_2^2} + \frac{AG_2\, l_1^2 \sin(\varphi)^2 \left(\omega_{OA}\right)^2}{\sqrt{1 - \frac{l_1^2 \sin(\varphi)^2}{l_2^2}}\; l_2^2};$$

$$\#a_{G2y} := \varphi \to \frac{\left(-l_2 + AG_2\right) l_1 \left(\omega_{OA}^{\;2} \sin(\varphi) - \varepsilon_{OA}(\varphi) \cos(\varphi)\right)}{l_2};$$

$$a_{G2y} := \varphi \to \left(1 - \frac{AG_2}{l_2}\right) l_1 \left(\varepsilon_{OA}(\varphi)\right) \cos(\varphi) - \left(1 - \frac{AG_2}{l_2}\right) l_1 \left(\omega_{OA}\right)^2 \sin(\varphi);$$

$n := 360;$

$XX_O := Matrix(n, 2)$:

$XX_A := Matrix(n, 2)$:

$YY_O := Matrix(n, 2)$:

$YY_A := Matrix(n, 2)$:

$MM := Matrix(n, 2)$:

$XX_B := Matrix(n, 2)$:

$YY_B := Matrix(n, 2) :$
$NN_B := Matrix(n, 2) :$
$FF_{ms} := Matrix(n, 2) :$
$RR_O := Matrix(n, 2) :$
$RR_A := Matrix(n, 2) :$
$RR_B := Matrix(n, 2) :$
$F := Matrix(n, 1) :$
for *i* **from** 271 **to** 360 **do**
$F[i] := -3233;$
od:
for *i* **from** 1 **by** 1 **to** *n* **do**

$$\varphi := \frac{(i-1)\cdot\pi}{180};$$

$$Sys\_dynamic := \Bigg\{$$

$$X_O + X_A + F_{dh}(\varphi)\cdot\frac{x_N(\varphi) - x_M(\varphi)}{MN(\varphi)} = m_1\cdot a_{G1x}(\varphi),$$

$$Y_O + Y_A - m_1\cdot g + F_{dh}(\varphi)\cdot\frac{y_N(\varphi) - y_M(\varphi)}{MN(\varphi)} = m_1\cdot a_{G1y}(\varphi),$$

$$M + \left(x_A(\varphi) - x_{G1}(\varphi)\right)\cdot Y_A - \left(y_A(\varphi) - y_{G1}(\varphi)\right)\cdot X_A + \left(x_O(\varphi) - x_{G1}(\varphi)\right)\cdot Y_O - \left(y_O(\varphi) - y_{G1}(\varphi)\right)\cdot X_O + \left(x_M(\varphi) - x_{G1}(\varphi)\right)\cdot F_{dh}(\varphi)\cdot\frac{y_N(\varphi) - y_M(\varphi)}{MN(\varphi)} - \left(y_M(\varphi) - y_{G1}(\varphi)\right)\cdot F_{dh}(\varphi)\cdot\frac{x_N(\varphi) - x_M(\varphi)}{MN(\varphi)} = J_1\cdot\varepsilon_{OA}(\varphi),$$

$$-X_A + X_B + F_{dh}(\varphi)\cdot\frac{x_M(\varphi) - x_N(\varphi)}{MN(\varphi)} = m_2\cdot a_{G2x}(\varphi),$$

$$-Y_A + Y_B - m_2\cdot g + F_{dh}(\varphi)\cdot\frac{y_M(\varphi) - y_N(\varphi)}{MN(\varphi)} = m_2\cdot a_{G2y}(\varphi),$$

$$\left(x_A(\varphi) - x_{G2}(\varphi)\right)\cdot\left(-Y_A\right) - \left(y_A(\varphi) - y_{G2}(\varphi)\right)\cdot\left(-X_A\right) + \left(x_B(\varphi) - x_{G2}(\varphi)\right)\cdot Y_B - \left(y_B(\varphi) - y_{G2}(\varphi)\right)\cdot X_B + \left(x_N(\varphi) - x_{G2}(\varphi)\right)\cdot F_{dh}(\varphi)\cdot\frac{y_M(\varphi) - y_N(\varphi)}{MN(\varphi)} - \left(y_N(\varphi) - y_{G2}(\varphi)\right)\cdot F_{dh}(\varphi)\cdot\frac{x_M(\varphi) - x_N(\varphi)}{MN(\varphi)} = J_2\cdot\varepsilon_{AB}(\varphi),$$

$$Y_B + m_3\cdot g = N_B,$$

$$-\mu\cdot|N_B|\cdot\frac{v_B(\varphi)}{|v_B(\varphi)| + 10^{-6}} = F_{ms},$$

$-X_B + F_{ms} + F(i) = m_3 \cdot a_B(\varphi)$ }:

$fsolve(Sys\_dynamic, \{X_A, Y_A, X_O, Y_O, X_B, Y_B, M, N_B, F_{ms}\}) : assign(\%);$

$XX_O(i,1) := i; XX_A(i,1) := i; XX_B(i,1) = i; YY_O(i,1) := i; YY_A(i,1) := i; YY_B(i,1) = i; MM(i,1) := i; NN_B(i,1) := i; FF_{ms}(i,1) := i; RR_O(i,1) := i; RR_A(i,1) := i; RR_B(i,1) := i;$

$XX_O(i,2) := X_O; XX_A(i,2) := X_A; XX_B(i,2) = X_B; YY_O(i,2) := Y_O; YY_A(i,2) := Y_A; YY_B(i,2) = Y_B; MM(i,2) := M; NN_B(i,2) := N_B; FF_{ms}(i,2) := F_{ms};$

$RR_O(i,2) := \sqrt{(X_O)^2 + (Y_O)^2};$

$RR_A(i,2) := \sqrt{(X_A)^2 + (Y_A)^2};$

$RR_B(i,2) := \sqrt{(X_B)^2 + (Y_B)^2};$

$unassign('X_O','X_A','X_B','Y_O','Y_A','Y_B','M','N_B','F_{ms}');$

od:

return $\left(\frac{\text{abs}(MM[1,2]) + \text{abs}(MM[360,2])}{2} + add(\text{abs}(MM[i,2]), i = 2..359)\right) \cdot \frac{\pi}{180};$

end proc:

$DirectSearch{:-}Search(Dynamic\_E, \{\alpha_1 = 0..1, \alpha_2 = 0..1, k = 100..20000\}, checkexit = 10, tolerances = 10\text{^}(-14), evaluationlimit = 30000);$

Warning, initial point [alpha__1 = .9, alpha__2 = .9, k = .9] does not satisfy the inequality constraints; trying to find a feasible initial point

Warning, the new feasible initial point is [alpha__1 = .49705375248537, alpha__2 = .49705375248537, k = 100.00000000015]

$$\left[732.414445920194, \begin{bmatrix} 0.917411777014075 \\ 0.999999998624930 \\ 12005.2237913409 \end{bmatrix}, 2294\right] \qquad \textbf{(3)}$$

$Dynamic\_M := \textbf{proc}(\alpha_1, \alpha_2, k)$

local $\varphi, \varepsilon_{OA}, l_1, l_2, \omega_{OA}, m_1, m_2, m_3, g, \mu, F, x_A, y_A, x_B, y_B, x_{G1}, y_{G1}, x_{G2}, y_{G2}, x_O, y_O, x_M, y_M, x_N, y_N, l_0, MN, \Delta l, F_{dh}, \varepsilon_{AB}, v_B, a_B, a_{G1x}, a_{G1y}, a_{G2x}, a_{G2y}, Sys\_dynamic, X_A, Y_A, X_O, Y_O, X_B, Y_B, M, N_B, F_{ms}, n, XX_O, XX_A, XX_B, YY_O, YY_A, YY_B, MM, NN_B, FF_{ms}, i, RR_O, RR_A, RR_B, J_1, J_2, AG_2, AG_1;$

#if not is({args}, set(numeric)) **then return** ('*procname*')('*args*') **end if**;

$l_1 := 0.175; l_2 := 0.58; \omega_{OA} := 3.1416; m_1 := 41.5147; m_2 := 7.656875; m_3 := 9.8996; g := 9.81; \mu := 0.3; AG_2 := 0.3165732; AG_1 := 0.0019;$

$J_1 := 0.889678;$

$J_2 := 0.6204482568;$

$x_A := \varphi \rightarrow l_1 \cdot \cos(\varphi);$

$y_A := \varphi \rightarrow l_1 \cdot \sin(\varphi);$

$$x_B := \varphi \to l_1 \cdot \cos(\varphi) + \left(l_2^{\,2} - \left(l_1 \cdot \sin(\varphi)\right)^2\right)^{\frac{1}{2}};$$

$$y_B := \varphi \to 0;$$

$$x_{G1} := \varphi \to AG_1 \cdot \cos(\varphi);$$

$$y_{G1} := \varphi \to AG_1 \cdot \sin(\varphi);$$

$$x_{G2} := \varphi \to x_A(\varphi) + AG_2 \cdot \sqrt{1 - \frac{l_1^{\,2}}{l_2^{\,2}} \cdot (\sin(\varphi))^2};$$

$$y_{G2} := \varphi \to \left(1 - \frac{AG_2}{l_2}\right) \cdot y_A(\varphi);$$

$$x_{\mathrm{O}} := \varphi \to 0;$$

$$y_{\mathrm{O}} := \varphi \to 0;$$

$$v_B := \varphi \to -\left(1 + \frac{l_1 \cdot \cos(\varphi)}{\left(l_2^{\,2} - l_1^{\,2} \cdot (\sin(\varphi))^2\right)^{\frac{1}{2}}}\right) \cdot \omega_{OA} \cdot l_1 \cdot \sin(\varphi);$$

$$x_M := \varphi \to \alpha_1 \cdot l_1 \cdot \cos(\varphi);$$

$$y_M := \varphi \to \alpha_1 \cdot l_1 \cdot \sin(\varphi);$$

$$x_N := \varphi \to l_1 \cdot \cos(\varphi) + \alpha_2 \cdot \sqrt{l_2^{\,2} - l_1^{\,2} \cdot (\sin(\varphi))^2};$$

$$y_N := \varphi \to \left(1 - \alpha_2\right) \cdot l_1 \cdot \sin(\varphi);$$

$$l_0 := \varphi \to \left(1 - \alpha_1\right) \cdot l_1 + \alpha_2 \cdot l_2;$$

$$MN := \varphi \to 10^{-10} + \sqrt{\left(x_M(\varphi) - x_N(\varphi)\right)^2 + \left(y_M(\varphi) - y_N(\varphi)\right)^2};$$

$$\Delta l := \varphi \to MN(\varphi) - l_0(\varphi);$$

$$F_{dh} := \varphi \to k \cdot \Delta l(\varphi);$$

$$\varepsilon_{OA} := \varphi \to 0;$$

$$\varepsilon_{AB} := \varphi \to \frac{l_2^{\,2} - l_1^{\,2}}{\left(l_2^{\,2} - l_1^{\,2} \cdot (\sin(\varphi))^2\right)^{\frac{3}{2}}} \cdot \left(\omega_{OA}\right)^2 \cdot l_1 \cdot \sin(\varphi) - \frac{l_1 \cdot \cos(\varphi)}{\left(l_2^{\,2} - l_1^{\,2} \cdot (\sin(\varphi))^2\right)^{\frac{1}{2}}} \cdot \varepsilon_{OA}(\varphi);$$

$$a_B := \varphi \to -\varepsilon_{OA}(\varphi) \cdot l_1 \cdot \sin(\varphi) \cdot \left(1 + \frac{l_1 \cdot \cos(\varphi)}{\left(l_2^{\,2} - l_1^{\,2} \cdot (\sin(\varphi))^2\right)^{\frac{1}{2}}}\right) + l_1 \cdot \omega_{OA}^{\,2}$$

$$\cdot \left(\frac{l_2^{\,2} - l_1^{\,2}}{\left(l_2^{\,2} - l_1^{\,2} \cdot (\sin(\varphi))^2\right)^{\frac{3}{2}}} \cdot l_1 \cdot (\sin(\varphi))^2 - \cos(\varphi) - \frac{l_1 \cdot (\cos(\varphi))^2}{\left(l_2^{\,2} - l_1^{\,2} \cdot (\sin(\varphi))^2\right)^{\frac{1}{2}}}\right);$$

$$a_{G1x} := \varphi \to -AG_1 \cdot \left(\varepsilon_{OA}(\varphi) \cdot \sin(\varphi) + \omega_{OA}^{\,2} \cdot \cos(\varphi)\right);$$

$$a_{G1y} := \varphi \to AG_1 \cdot \left(\varepsilon_{OA}(\varphi) \cdot \cos(\varphi) - \omega_{OA}^{\,2} \cdot \sin(\varphi)\right);$$

$$\#a_{G2x} := \varphi \rightarrow -l_1 \sin(\varphi) \left(1 + \frac{AG_2\, l_1 \cos(\varphi)}{\sqrt{1 - \frac{l_1^2 \sin(\varphi)^2}{l_2^2}}\; l_2}\right) \varepsilon_{OA}(\varphi) - l_1\, \omega_{OA}^{\;2} \left(\cos(\varphi) + \frac{AG_2\, l_1^3 \sin(\varphi)^2 \cos(\varphi)^2}{\left(1 - \frac{l_1^2 \sin(\varphi)^2}{l_2^2}\right)^{3/2} l_2^4} + \frac{AG_2\, l_1 \cos(2\,\varphi)}{\sqrt{1 - \frac{l_1^2 \sin(\varphi)^2}{l_2^2}}\; l_2^2}\right);$$

$$a_{G2x} := \varphi \rightarrow -l_1 \left(\varepsilon_{OA}(\varphi)\right) \sin(\varphi) - l_1 \left(\omega_{OA}\right)^2 \cos(\varphi) - \frac{AG_2\, l_1^4 \sin(\varphi)^2 \left(\omega_{OA}\right)^2 \cos(\varphi)^2}{\left(1 - \frac{l_1^2 \sin(\varphi)^2}{l_2^2}\right)^{3/2} l_2^4} - \frac{AG_2\, l_1^2 \left(\omega_{OA}\right)^2 \cos(\varphi)^2}{\sqrt{1 - \frac{l_1^2 \sin(\varphi)^2}{l_2^2}}\; l_2^2} - \frac{AG_2\, l_1^2 \sin(\varphi) \left(\varepsilon_{OA}(\varphi)\right) \cos(\varphi)}{\sqrt{1 - \frac{l_1^2 \sin(\varphi)^2}{l_2^2}}\; l_2^2} + \frac{AG_2\, l_1^2 \sin(\varphi)^2 \left(\omega_{OA}\right)^2}{\sqrt{1 - \frac{l_1^2 \sin(\varphi)^2}{l_2^2}}\; l_2^2};$$

$$\#a_{G2y} := \varphi \rightarrow \frac{\left(-l_2 + AG_2\right) l_1 \left(\omega_{OA}^{\;2} \sin(\varphi) - \varepsilon_{OA}(\varphi) \cos(\varphi)\right)}{l_2};$$

$$a_{G2y} := \varphi \rightarrow \left(1 - \frac{AG_2}{l_2}\right) l_1 \left(\varepsilon_{OA}(\varphi)\right) \cos(\varphi) - \left(1 - \frac{AG_2}{l_2}\right) l_1 \left(\omega_{OA}\right)^2 \sin(\varphi);$$

$n := 360;$
$XX_{\mathrm{O}} := Matrix(n, 2):$
$XX_A := Matrix(n, 2):$
$YY_{\mathrm{O}} := Matrix(n, 2):$
$YY_A := Matrix(n, 2):$
$MM := Matrix(n, 2):$
$XX_B := Matrix(n, 2):$
$YY_B := Matrix(n, 2):$
$NN_B := Matrix(n, 2):$
$FF_{ms} := Matrix(n, 2):$
$RR_{\mathrm{O}} := Matrix(n, 2):$
$RR_A := Matrix(n, 2):$
$RR_B := Matrix(n, 2):$
$F := Matrix(n, 1):$
for *i* **from** 271 **to** 360 **do**
$F[i] := -3233;$
od:
for *i* **from** 1 **by** 1 **to** *n* **do**

$$\varphi := \frac{(i-1)\cdot\pi}{180};$$

$$Sys\_dynamic := \{$$

$$X_O + X_A + F_{dh}(\varphi)\cdot\frac{x_N(\varphi) - x_M(\varphi)}{MN(\varphi)} = m_1\cdot a_{G1x}(\varphi),$$

$$Y_O + Y_A - m_1\cdot g + F_{dh}(\varphi)\cdot\frac{y_N(\varphi) - y_M(\varphi)}{MN(\varphi)} = m_1\cdot a_{G1y}(\varphi),$$

$$M + \left(x_A(\varphi) - x_{G1}(\varphi)\right)\cdot Y_A - \left(y_A(\varphi) - y_{G1}(\varphi)\right)\cdot X_A + \left(x_O(\varphi) - x_{G1}(\varphi)\right)\cdot Y_O - \left(y_O(\varphi) - y_{G1}(\varphi)\right)\cdot X_O + \left(x_M(\varphi) - x_{G1}(\varphi)\right)\cdot F_{dh}(\varphi)\cdot\frac{y_N(\varphi) - y_M(\varphi)}{MN(\varphi)} - \left(y_M(\varphi) - y_{G1}(\varphi)\right)\cdot F_{dh}(\varphi)\cdot\frac{x_N(\varphi) - x_M(\varphi)}{MN(\varphi)} = J_1\cdot\varepsilon_{OA}(\varphi),$$

$$-X_A + X_B + F_{dh}(\varphi)\cdot\frac{x_M(\varphi) - x_N(\varphi)}{MN(\varphi)} = m_2\cdot a_{G2x}(\varphi),$$

$$-Y_A + Y_B - m_2\cdot g + F_{dh}(\varphi)\cdot\frac{y_M(\varphi) - y_N(\varphi)}{MN(\varphi)} = m_2\cdot a_{G2y}(\varphi),$$

$$\left(x_A(\varphi) - x_{G2}(\varphi)\right)\cdot\left(-Y_A\right) - \left(y_A(\varphi) - y_{G2}(\varphi)\right)\cdot\left(-X_A\right) + \left(x_B(\varphi) - x_{G2}(\varphi)\right)\cdot Y_B - \left(y_B(\varphi) - y_{G2}(\varphi)\right)\cdot X_B + \left(x_N(\varphi) - x_{G2}(\varphi)\right)\cdot F_{dh}(\varphi)\cdot\frac{y_M(\varphi) - y_N(\varphi)}{MN(\varphi)} - \left(y_N(\varphi) - y_{G2}(\varphi)\right)\cdot F_{dh}(\varphi)\cdot\frac{x_M(\varphi) - x_N(\varphi)}{MN(\varphi)} = J_2\cdot\varepsilon_{AB}(\varphi),$$

$$Y_B + m_3\cdot g = N_B,$$

$$-\mu\cdot|N_B|\cdot\frac{v_B(\varphi)}{|v_B(\varphi)| + 10^{-6}} = F_{ms},$$

$$-X_B + F_{ms} + F(i) = m_3\cdot a_B(\varphi)\ \}:$$

$fsolve\left(Sys\_dynamic, \{X_A, Y_A, X_O, Y_O, X_B, Y_B, M, N_B, F_{ms}\}\right) : assign(\%);$

$XX_O(i,1) := i; XX_A(i,1) := i; XX_B(i,1) = i; YY_O(i,1) := i; YY_A(i,1) := i; YY_B(i,1) = i; MM(i,1) := i; NN_B(i,1) := i; FF_{ms}(i,1) := i; RR_O(i,1) := i; RR_A(i,1) := i; RR_B(i,1) := i;$

$XX_O(i,2) := X_O; XX_A(i,2) := X_A; XX_B(i,2) = X_B; YY_O(i,2) := Y_O; YY_A(i,2) := Y_A; YY_B(i,2) = Y_B; MM(i,2) := M; NN_B(i,2) := N_B; FF_{ms}(i,2) := F_{ms};$

$RR_O(i,2) := \sqrt{(X_O)^2 + (Y_O)^2};$

$RR_A(i,2) := \sqrt{(X_A)^2 + (Y_A)^2};$

$RR_B(i,2) := \sqrt{(X_B)^2 + (Y_B)^2};$

$unassign('X_O','X_A','X_B','Y_O','Y_A','Y_B','M','N_B','F_{ms}');$

od:

return max(|*MM*|);
end proc:

$DirectSearch\text{:-}Search\left(Dynamic\_M, \{\alpha_1 = 0..1, \alpha_2 = 0..1, k = 100..20000\}, checkexit = 10, tolerances = 10^{(-14)}, evaluationlimit = 30000\right);$

Warning, initial point [alpha__1 = .9, alpha__2 = .9, k = .9] does not satisfy the inequality constraints; trying to find a feasible initial point

Warning, the new feasible initial point is [alpha__1 = .49705375248537, alpha__2 = .49705375248537, k = 100.00000000015]

$$\left[405.775860974807, \begin{bmatrix} 0.277949837615229 \\ 0.999999999999999 \\ 18997.7175886331 \end{bmatrix}, 2437\right] \quad \textbf{(4)}$$
