# Supplementary material for: Multi-objective optimizing spring placement and stiffness in slider-crank mechanisms for enhanced dynamic parameters
Source: PLoS One. 2025 Sep 8;20(9):e0331341. doi: 10.1371/journal.pone.0331341 (PMC12416651; doi:10.1371/journal.pone.0331341)
Supplement: S1 Data — (ZIP) [file pone.0331341.s001.ZIP › Multiobjective_Hybrid_CDOS_PSI_Stage2_(For Table 2-6,Fig 12-13).pdf]

### *VI DU*

`restart:`

**#Number of parameters, criteria and functional dependencies: (each task has its own)**

$NParameters := 3 :$ # *Number of Parameter*

$NCriterias := 2 :$ # *Number of criteria*

$NFunctional := 2 :$ # *Number of functional dependencies*

**Expressions for functional constraints (procedures): (each task has its own)**

#*f1=RA*

$f1 :=$**proc**$(x1, x2, x3)$

**local** $\alpha_1, \alpha_2, k, \varphi, \varepsilon_{OA}, l_1, l_2, \omega_{OA}, m_1, m_2, m_3, g, \mu, F, x_A, y_A, x_B, y_B, x_{G1}, y_{G1}, x_{G2}, y_{G2}, x_O, y_O, x_M, y_M, x_N, y_N, l_0, MN, \Delta l, F_{dh}, \varepsilon_{AB}, v_B, a_B, a_{G1x}, a_{G1y}, a_{G2x}, a_{G2y}, Sys\_dynamic, X_A, Y_A, X_O, Y_O, X_B, Y_B, M, N_B, F_{ms}, n, XX_O, XX_A, XX_B, YY_O, YY_A, YY_B, MM, NN_B, FF_{ms}, i, RR_O, RR_A, RR_B, J_1, J_2, AG_2, AG_1;$

#*if not is*( {*args*}, *set*(*numeric*) ) **then return** ('*procname*')('*args*') **end if**;

$\alpha_1 := x1; \alpha_2 := x2; k := x3;$

$l_1 := 0.175; l_2 := 0.58; \omega_{OA} := 3.1416; m_1 := 41.5147; m_2 := 7.656875; m_3 := 9.8996; g := 9.81; \mu := 0.3; AG_2 := 0.3165732; AG_1 := 0.0019;$

$J_1 := 0.889678;$

$J_2 := 0.6204482568;$

$x_A := \varphi \rightarrow l_1 \cdot \cos(\varphi);$

$y_A := \varphi \rightarrow l_1 \cdot \sin(\varphi);$

$$x_B := \varphi \rightarrow l_1 \cdot \cos(\varphi) + \left(l_2^2 - \left(l_1 \cdot \sin(\varphi)\right)^2\right)^{\frac{1}{2}};$$

$y_B := \varphi \rightarrow 0;$

$x_{G1} := \varphi \rightarrow AG_1 \cdot \cos(\varphi);$

$y_{G1} := \varphi \rightarrow AG_1 \cdot \sin(\varphi);$

$$x_{G2} := \varphi \rightarrow x_A(\varphi) + AG_2 \cdot \sqrt{1 - \frac{l_1^2}{l_2^2} \cdot \left(\sin(\varphi)\right)^2};$$

$$y_{G2} := \varphi \rightarrow \left(1 - \frac{AG_2}{l_2}\right) \cdot y_A(\varphi);$$

$x_O := \varphi \rightarrow 0;$

$y_O := \varphi \rightarrow 0;$

$$v_B := \varphi \rightarrow -\left(1 + \frac{l_1 \cdot \cos(\varphi)}{\left(l_2^2 - l_1^2 \cdot \left(\sin(\varphi)\right)^2\right)^{\frac{1}{2}}}\right) \cdot \omega_{OA} \cdot l_1 \cdot \sin(\varphi);$$

$x_M := \varphi \rightarrow \alpha_1 \cdot l_1 \cdot \cos(\varphi);$

$$y_M := \varphi \rightarrow \alpha_1 \cdot l_1 \cdot \sin(\varphi);$$

$$x_N := \varphi \rightarrow l_1 \cdot \cos(\varphi) + \alpha_2 \cdot \sqrt{l_2^2 - l_1^2 \cdot (\sin(\varphi))^2};$$

$$y_N := \varphi \rightarrow (1 - \alpha_2) \cdot l_1 \cdot \sin(\varphi);$$

$$l_0 := \varphi \rightarrow (1 - \alpha_1) \cdot l_1 + \alpha_2 \cdot l_2;$$

$$MN := \varphi \rightarrow 10^{-10} + \sqrt{(x_M(\varphi) - x_N(\varphi))^2 + (y_M(\varphi) - y_N(\varphi))^2};$$

$$\Delta l := \varphi \rightarrow MN(\varphi) - l_0(\varphi);$$

$$F_{dh} := \varphi \rightarrow k \cdot \Delta l(\varphi);$$

$$\varepsilon_{OA} := \varphi \rightarrow 0;$$

$$\varepsilon_{AB} := \varphi \rightarrow \frac{l_2^2 - l_1^2}{\left(l_2^2 - l_1^2 \cdot (\sin(\varphi))^2\right)^{\frac{3}{2}}} \cdot (\omega_{OA})^2 \cdot l_1 \cdot \sin(\varphi) - \frac{l_1 \cdot \cos(\varphi)}{\left(l_2^2 - l_1^2 \cdot (\sin(\varphi))^2\right)^{\frac{1}{2}}} \cdot \varepsilon_{OA}(\varphi);$$

$$a_B := \varphi \rightarrow -\varepsilon_{OA}(\varphi) \cdot l_1 \cdot \sin(\varphi) \cdot \left(1 + \frac{l_1 \cdot \cos(\varphi)}{\left(l_2^2 - l_1^2 \cdot (\sin(\varphi))^2\right)^{\frac{1}{2}}}\right) + l_1 \cdot \omega_{OA}^2 \cdot \left(\frac{l_2^2 - l_1^2}{\left(l_2^2 - l_1^2 \cdot (\sin(\varphi))^2\right)^{\frac{3}{2}}} \cdot l_1 \cdot (\sin(\varphi))^2 - \cos(\varphi) - \frac{l_1 \cdot (\cos(\varphi))^2}{\left(l_2^2 - l_1^2 \cdot (\sin(\varphi))^2\right)^{\frac{1}{2}}}\right);$$

$$a_{G1x} := \varphi \rightarrow -AG_1 \cdot \left(\varepsilon_{OA}(\varphi) \cdot \sin(\varphi) + \omega_{OA}^2 \cdot \cos(\varphi)\right);$$

$$a_{G1y} := \varphi \rightarrow AG_1 \cdot \left(\varepsilon_{OA}(\varphi) \cdot \cos(\varphi) - \omega_{OA}^2 \cdot \sin(\varphi)\right);$$

$$\#a_{G2x} := \varphi \rightarrow -l_1 \sin(\varphi) \left(1 + \frac{AG_2\, l_1 \cos(\varphi)}{\sqrt{1 - \frac{l_1^2 \sin(\varphi)^2}{l_2^2}}\; l_2}\right) \varepsilon_{OA}(\varphi) - l_1\, \omega_{OA}^2 \left(\cos(\varphi) + \frac{AG_2\, l_1^3 \sin(\varphi)^2 \cos(\varphi)^2}{\left(1 - \frac{l_1^2 \sin(\varphi)^2}{l_2^2}\right)^{3/2} l_2^4} + \frac{AG_2\, l_1 \cos(2\,\varphi)}{\sqrt{1 - \frac{l_1^2 \sin(\varphi)^2}{l_2^2}}\; l_2^2}\right);$$

$$a_{G2x} := \varphi \rightarrow -l_1 \left(\varepsilon_{OA}(\varphi)\right) \sin(\varphi) - l_1 \left(\omega_{OA}\right)^2 \cos(\varphi) - \frac{AG_2\, l_1^4 \sin(\varphi)^2 \left(\omega_{OA}\right)^2 \cos(\varphi)^2}{\left(1 - \frac{l_1^2 \sin(\varphi)^2}{l_2^2}\right)^{3/2} l_2^4} - \frac{AG_2\, l_1^2 \left(\omega_{OA}\right)^2 \cos(\varphi)^2}{\sqrt{1 - \frac{l_1^2 \sin(\varphi)^2}{l_2^2}}\; l_2^2} - \frac{AG_2\, l_1^2 \sin(\varphi) \left(\varepsilon_{OA}(\varphi)\right) \cos(\varphi)}{\sqrt{1 - \frac{l_1^2 \sin(\varphi)^2}{l_2^2}}\; l_2^2}$$

$$+\frac{AG_2\, l_1^{\,2}\sin(\varphi)^2\left(\omega_{OA}\right)^2}{\sqrt{1-\frac{l_1^{\,2}\sin(\varphi)^2}{l_2^{\,2}}}\; l_2^{\,2}};$$

$$\#a_{G2y} := \varphi \rightarrow \frac{\left(-l_2+AG_2\right) l_1 \left(\omega_{OA}^{\;2}\sin(\varphi)-\varepsilon_{OA}(\varphi)\cos(\varphi)\right)}{l_2};$$

$$a_{G2y} := \varphi \rightarrow \left(1-\frac{AG_2}{l_2}\right) l_1 \left(\varepsilon_{OA}(\varphi)\right)\cos(\varphi)-\left(1-\frac{AG_2}{l_2}\right) l_1 \left(\omega_{OA}\right)^2\sin(\varphi);$$

$n := 360;$
$XX_{\mathrm{O}} := Matrix(n, 2):$
$XX_A := Matrix(n, 2):$
$YY_{\mathrm{O}} := Matrix(n, 2):$
$YY_A := Matrix(n, 2):$
$MM := Matrix(n, 2):$
$XX_B := Matrix(n, 2):$
$YY_B := Matrix(n, 2):$
$NN_B := Matrix(n, 2):$
$FF_{ms} := Matrix(n, 2):$
$RR_{\mathrm{O}} := Matrix(n, 2):$
$RR_A := Matrix(n, 2):$
$RR_B := Matrix(n, 2):$
$F := Matrix(n, 1):$
**for** *i* **from** 271 **to** 360 **do**
$F[i] := -3233;$
**od**:
**for** *i* **from** 1 **by** 1 **to** *n* **do**

$$\varphi := \frac{(i-1)\cdot\pi}{180};$$

*Sys_dynamic* :=

$$X_{\mathrm{O}}+X_A+F_{dh}(\varphi)\cdot\frac{x_N(\varphi)-x_M(\varphi)}{MN(\varphi)}=m_1\cdot a_{G1x}(\varphi),$$

$$Y_{\mathrm{O}}+Y_A-m_1\cdot g+F_{dh}(\varphi)\cdot\frac{y_N(\varphi)-y_M(\varphi)}{MN(\varphi)}=m_1\cdot a_{G1y}(\varphi),$$

$$M+\left(x_A(\varphi)-x_{G1}(\varphi)\right)\cdot Y_A-\left(y_A(\varphi)-y_{G1}(\varphi)\right)\cdot X_A+\left(x_{\mathrm{O}}(\varphi)-x_{G1}(\varphi)\right)\cdot Y_{\mathrm{O}}-\left(y_{\mathrm{O}}(\varphi)-y_{G1}(\varphi)\right)\cdot X_{\mathrm{O}}+\left(x_M(\varphi)-x_{G1}(\varphi)\right)\cdot F_{dh}(\varphi)\cdot\frac{y_N(\varphi)-y_M(\varphi)}{MN(\varphi)}-\left(y_M(\varphi)-y_{G1}(\varphi)\right)\cdot F_{dh}(\varphi)\cdot\frac{x_N(\varphi)-x_M(\varphi)}{MN(\varphi)}=J_1\cdot\varepsilon_{OA}(\varphi),$$

$$-X_A+X_B+F_{dh}(\varphi)\cdot\frac{x_M(\varphi)-x_N(\varphi)}{MN(\varphi)}=m_2\cdot a_{G2x}(\varphi),$$

$$-Y_A+Y_B-m_2\cdot g+F_{dh}(\varphi)\cdot\frac{y_M(\varphi)-y_N(\varphi)}{MN(\varphi)}=m_2\cdot a_{G2y}(\varphi),$$

$$(x_A(\varphi)-x_{G2}(\varphi))\cdot(-Y_A)-(y_A(\varphi)-y_{G2}(\varphi))\cdot(-X_A)+(x_B(\varphi)-x_{G2}(\varphi))\cdot Y_B-(y_B(\varphi)-y_{G2}(\varphi))\cdot X_B+(x_N(\varphi)-x_{G2}(\varphi))\cdot F_{dh}(\varphi)\cdot\frac{y_M(\varphi)-y_N(\varphi)}{MN(\varphi)}-(y_N(\varphi)-y_{G2}(\varphi))\cdot F_{dh}(\varphi)\cdot\frac{x_M(\varphi)-x_N(\varphi)}{MN(\varphi)}=J_2\cdot\varepsilon_{AB}(\varphi),$$

$$Y_B+m_3\cdot g=N_B,$$

$$-\mu\cdot|N_B|\cdot\frac{v_B(\varphi)}{|v_B(\varphi)|+10^{-6}}=F_{ms},$$

$$\left.-X_B+F_{ms}+F(i)=m_3\cdot a_B(\varphi)\right\}:$$

$fsolve(Sys\_dynamic, \{X_A, Y_A, X_O, Y_O, X_B, Y_B, M, N_B, F_{ms}\}) : assign(\%);$

$XX_O(i,1) := i; XX_A(i,1) := i; XX_B(i,1) = i; YY_O(i,1) := i; YY_A(i,1) := i; YY_B(i,1) = i; MM(i,1) := i; NN_B(i,1) := i; FF_{ms}(i,1) := i; RR_O(i,1) := i; RR_A(i,1) := i; RR_B(i,1) := i;$

$XX_O(i,2) := X_O; XX_A(i,2) := X_A; XX_B(i,2) = X_B; YY_O(i,2) := Y_O; YY_A(i,2) := Y_A; YY_B(i,2) = Y_B; MM(i,2) := M; NN_B(i,2) := N_B; FF_{ms}(i,2) := F_{ms};$

$RR_O(i,2) := \sqrt{(X_O)^2+(Y_O)^2};$

$RR_A(i,2) := \sqrt{(X_A)^2+(Y_A)^2};$

$RR_B(i,2) := \sqrt{(X_B)^2+(Y_B)^2};$

$unassign('X_O','X_A','X_B','Y_O','Y_A','Y_B','M','N_B','F_{ms}');$

**od**:

**return** $\max(|RR_A|);$

**end proc**:

*#f2=NB*

$f2 :=$ **proc**$(x1, x2, x3)$

**local** $\alpha_1, \alpha_2, k, \varphi, \varepsilon_{OA}, l_1, l_2, \omega_{OA}, m_1, m_2, m_3, g, \mu, F, x_A, y_A, x_B, y_B, x_{G1}, y_{G1}, x_{G2}, y_{G2}, x_O, y_O, x_M, y_M, x_N, y_N, l_0, MN, \Delta l, F_{dh}, \varepsilon_{AB}, v_B, a_B, a_{G1x}, a_{G1y}, a_{G2x}, a_{G2y}, Sys\_dynamic, X_A, Y_A, X_O, Y_O, X_B, Y_B, M, N_B, F_{ms}, n, XX_O, XX_A, XX_B, YY_O, YY_A, YY_B, MM, NN_B, FF_{ms}, i, RR_O, RR_A, RR_B, J_1, J_2, AG_2, AG_1;$

*#if not is({args}, set(numeric))* **then return** *('procname')('args')* **end if**;

$\alpha_1 := x1; \alpha_2 := x2; k := x3;$

$l_1 := 0.175; l_2 := 0.58; \omega_{OA} := 3.1416; m_1 := 41.5147; m_2 := 7.656875; m_3 := 9.8996; g := 9.81; \mu := 0.3; AG_2 := 0.3165732; AG_1 := 0.0019;$

$J_1 := 0.889678;$

$J_2 := 0.6204482568;$

$$x_A := \varphi \to l_1 \cdot \cos(\varphi);$$
$$y_A := \varphi \to l_1 \cdot \sin(\varphi);$$
$$x_B := \varphi \to l_1 \cdot \cos(\varphi) + \left(l_2^2 - \left(l_1 \cdot \sin(\varphi)\right)^2\right)^{\frac{1}{2}};$$
$$y_B := \varphi \to 0;$$
$$x_{G1} := \varphi \to AG_1 \cdot \cos(\varphi);$$
$$y_{G1} := \varphi \to AG_1 \cdot \sin(\varphi);$$
$$x_{G2} := \varphi \to x_A(\varphi) + AG_2 \cdot \sqrt{1 - \frac{l_1^2}{l_2^2} \cdot (\sin(\varphi))^2};$$
$$y_{G2} := \varphi \to \left(1 - \frac{AG_2}{l_2}\right) \cdot y_A(\varphi);$$
$$x_O := \varphi \to 0;$$
$$y_O := \varphi \to 0;$$
$$v_B := \varphi \to -\left(1 + \frac{l_1 \cdot \cos(\varphi)}{\left(l_2^2 - l_1^2 \cdot (\sin(\varphi))^2\right)^{\frac{1}{2}}}\right) \cdot \omega_{OA} \cdot l_1 \cdot \sin(\varphi);$$
$$x_M := \varphi \to \alpha_1 \cdot l_1 \cdot \cos(\varphi);$$
$$y_M := \varphi \to \alpha_1 \cdot l_1 \cdot \sin(\varphi);$$
$$x_N := \varphi \to l_1 \cdot \cos(\varphi) + \alpha_2 \cdot \sqrt{l_2^2 - l_1^2 \cdot (\sin(\varphi))^2};$$
$$y_N := \varphi \to \left(1 - \alpha_2\right) \cdot l_1 \cdot \sin(\varphi);$$
$$l_0 := \varphi \to \left(1 - \alpha_1\right) \cdot l_1 + \alpha_2 \cdot l_2;$$
$$MN := \varphi \to 10^{-10} + \sqrt{\left(x_M(\varphi) - x_N(\varphi)\right)^2 + \left(y_M(\varphi) - y_N(\varphi)\right)^2};$$
$$\Delta l := \varphi \to MN(\varphi) - l_0(\varphi);$$
$$F_{dh} := \varphi \to k \cdot \Delta l(\varphi);$$
$$\varepsilon_{OA} := \varphi \to 0;$$
$$\varepsilon_{AB} := \varphi \to \frac{l_2^2 - l_1^2}{\left(l_2^2 - l_1^2 \cdot (\sin(\varphi))^2\right)^{\frac{3}{2}}} \cdot \left(\omega_{OA}\right)^2 \cdot l_1 \cdot \sin(\varphi) - \frac{l_1 \cdot \cos(\varphi)}{\left(l_2^2 - l_1^2 \cdot (\sin(\varphi))^2\right)^{\frac{1}{2}}} \cdot \varepsilon_{OA}(\varphi);$$
$$a_B := \varphi \to -\varepsilon_{OA}(\varphi) \cdot l_1 \cdot \sin(\varphi) \cdot \left(1 + \frac{l_1 \cdot \cos(\varphi)}{\left(l_2^2 - l_1^2 \cdot (\sin(\varphi))^2\right)^{\frac{1}{2}}}\right) + l_1 \cdot \omega_{OA}^2$$
$$\cdot \left(\frac{l_2^2 - l_1^2}{\left(l_2^2 - l_1^2 \cdot (\sin(\varphi))^2\right)^{\frac{3}{2}}} \cdot l_1 \cdot (\sin(\varphi))^2 - \cos(\varphi) - \frac{l_1 \cdot (\cos(\varphi))^2}{\left(l_2^2 - l_1^2 \cdot (\sin(\varphi))^2\right)^{\frac{1}{2}}}\right);$$
$$a_{G1x} := \varphi \to -AG_1 \cdot \left(\varepsilon_{OA}(\varphi) \cdot \sin(\varphi) + \omega_{OA}^2 \cdot \cos(\varphi)\right);$$
$$a_{G1y} := \varphi \to AG_1 \cdot \left(\varepsilon_{OA}(\varphi) \cdot \cos(\varphi) - \omega_{OA}^2 \cdot \sin(\varphi)\right);$$

$$\#a_{G2x} := \varphi \rightarrow -l_1 \sin(\varphi) \left(1 + \frac{AG_2\, l_1 \cos(\varphi)}{\sqrt{1 - \frac{l_1^2 \sin(\varphi)^2}{l_2^2}}\; l_2}\right) \varepsilon_{OA}(\varphi) - l_1\, \omega_{OA}^{\;2} \left(\cos(\varphi) + \frac{AG_2\, l_1^3 \sin(\varphi)^2 \cos(\varphi)^2}{\left(1 - \frac{l_1^2 \sin(\varphi)^2}{l_2^2}\right)^{3/2} l_2^4} + \frac{AG_2\, l_1 \cos(2\,\varphi)}{\sqrt{1 - \frac{l_1^2 \sin(\varphi)^2}{l_2^2}}\; l_2}\right);$$

$$a_{G2x} := \varphi \rightarrow -l_1 \left(\varepsilon_{OA}(\varphi)\right) \sin(\varphi) - l_1 \left(\omega_{OA}\right)^2 \cos(\varphi) - \frac{AG_2\, l_1^4 \sin(\varphi)^2 \left(\omega_{OA}\right)^2 \cos(\varphi)^2}{\left(1 - \frac{l_1^2 \sin(\varphi)^2}{l_2^2}\right)^{3/2} l_2^4} - \frac{AG_2\, l_1^2 \left(\omega_{OA}\right)^2 \cos(\varphi)^2}{\sqrt{1 - \frac{l_1^2 \sin(\varphi)^2}{l_2^2}}\; l_2} - \frac{AG_2\, l_1^2 \sin(\varphi) \left(\varepsilon_{OA}(\varphi)\right) \cos(\varphi)}{\sqrt{1 - \frac{l_1^2 \sin(\varphi)^2}{l_2^2}}\; l_2} + \frac{AG_2\, l_1^2 \sin(\varphi)^2 \left(\omega_{OA}\right)^2}{\sqrt{1 - \frac{l_1^2 \sin(\varphi)^2}{l_2^2}}\; l_2};$$

$$\#a_{G2y} := \varphi \rightarrow \frac{\left(-l_2 + AG_2\right) l_1 \left(\omega_{OA}^{\;2} \sin(\varphi) - \varepsilon_{OA}(\varphi) \cos(\varphi)\right)}{l_2};$$

$$a_{G2y} := \varphi \rightarrow \left(1 - \frac{AG_2}{l_2}\right) l_1 \left(\varepsilon_{OA}(\varphi)\right) \cos(\varphi) - \left(1 - \frac{AG_2}{l_2}\right) l_1 \left(\omega_{OA}\right)^2 \sin(\varphi);$$

$n := 360;$
$XX_{\mathrm{O}} := Matrix(n, 2):$
$XX_A := Matrix(n, 2):$
$YY_{\mathrm{O}} := Matrix(n, 2):$
$YY_A := Matrix(n, 2):$
$MM := Matrix(n, 2):$
$XX_B := Matrix(n, 2):$
$YY_B := Matrix(n, 2):$
$NN_B := Matrix(n, 2):$
$FF_{ms} := Matrix(n, 2):$
$RR_{\mathrm{O}} := Matrix(n, 2):$
$RR_A := Matrix(n, 2):$
$RR_B := Matrix(n, 2):$
$F := Matrix(n, 1):$
**for** *i* **from** 271 **to** 360 **do**
$F[i] := -3233;$
**od**:

**for** $i$ **from** 1 **by** 1 **to** $n$ **do**

$$\varphi := \frac{(i-1)\cdot\pi}{180};$$

$$Sys\_dynamic := \Big\{$$

$$X_O + X_A + F_{dh}(\varphi)\cdot\frac{x_N(\varphi) - x_M(\varphi)}{MN(\varphi)} = m_1\cdot a_{G1x}(\varphi),$$

$$Y_O + Y_A - m_1\cdot g + F_{dh}(\varphi)\cdot\frac{y_N(\varphi) - y_M(\varphi)}{MN(\varphi)} = m_1\cdot a_{G1y}(\varphi),$$

$$M + \left(x_A(\varphi) - x_{G1}(\varphi)\right)\cdot Y_A - \left(y_A(\varphi) - y_{G1}(\varphi)\right)\cdot X_A + \left(x_O(\varphi) - x_{G1}(\varphi)\right)\cdot Y_O - \left(y_O(\varphi) - y_{G1}(\varphi)\right)\cdot X_O + \left(x_M(\varphi) - x_{G1}(\varphi)\right)\cdot F_{dh}(\varphi)\cdot\frac{y_N(\varphi) - y_M(\varphi)}{MN(\varphi)} - \left(y_M(\varphi) - y_{G1}(\varphi)\right)\cdot F_{dh}(\varphi)\cdot\frac{x_N(\varphi) - x_M(\varphi)}{MN(\varphi)} = J_1\cdot\varepsilon_{OA}(\varphi),$$

$$-X_A + X_B + F_{dh}(\varphi)\cdot\frac{x_M(\varphi) - x_N(\varphi)}{MN(\varphi)} = m_2\cdot a_{G2x}(\varphi),$$

$$-Y_A + Y_B - m_2\cdot g + F_{dh}(\varphi)\cdot\frac{y_M(\varphi) - y_N(\varphi)}{MN(\varphi)} = m_2\cdot a_{G2y}(\varphi),$$

$$\left(x_A(\varphi) - x_{G2}(\varphi)\right)\cdot\left(-Y_A\right) - \left(y_A(\varphi) - y_{G2}(\varphi)\right)\cdot\left(-X_A\right) + \left(x_B(\varphi) - x_{G2}(\varphi)\right)\cdot Y_B - \left(y_B(\varphi) - y_{G2}(\varphi)\right)\cdot X_B + \left(x_N(\varphi) - x_{G2}(\varphi)\right)\cdot F_{dh}(\varphi)\cdot\frac{y_M(\varphi) - y_N(\varphi)}{MN(\varphi)} - \left(y_N(\varphi) - y_{G2}(\varphi)\right)\cdot F_{dh}(\varphi)\cdot\frac{x_M(\varphi) - x_N(\varphi)}{MN(\varphi)} = J_2\cdot\varepsilon_{AB}(\varphi),$$

$$Y_B + m_3\cdot g = N_B,$$

$$-\mu\cdot|N_B|\cdot\frac{v_B(\varphi)}{|v_B(\varphi)| + 10^{-6}} = F_{ms},$$

$$-X_B + F_{ms} + F(i) = m_3\cdot a_B(\varphi)\Big\}:$$

$fsolve(Sys\_dynamic, \{X_A, Y_A, X_O, Y_O, X_B, Y_B, M, N_B, F_{ms}\}) : assign(\%);$

$XX_O(i, 1) := i; XX_A(i, 1) := i; XX_B(i, 1) = i; YY_O(i, 1) := i; YY_A(i, 1) := i; YY_B(i, 1) = i; MM(i, 1) := i; NN_B(i, 1) := i; FF_{ms}(i, 1) := i; RR_O(i, 1) := i; RR_A(i, 1) := i; RR_B(i, 1) := i;$

$XX_O(i, 2) := X_O; XX_A(i, 2) := X_A; XX_B(i, 2) = X_B; YY_O(i, 2) := Y_O; YY_A(i, 2) := Y_A; YY_B(i, 2) = Y_B; MM(i, 2) := M; NN_B(i, 2) := N_B; FF_{ms}(i, 2) := F_{ms};$

$$RR_O(i, 2) := \sqrt{(X_O)^2 + (Y_O)^2};$$

$$RR_A(i, 2) := \sqrt{(X_A)^2 + (Y_A)^2};$$

$RR_B(i,2) := \sqrt{(X_B)^2 + (Y_B)^2};$
$unassign('X_O','X_A','X_B','Y_O','Y_A','Y_B','M','N_B','F_{ms}');$
**od**:
**return** $\max(|NN_B|);$
**end proc**:

## Criterion functions (procedures): (each task has its own)

*#Φ1=Energy*

$\Phi 1 :=$ **proc**$(x1, x2, x3)$

**local** $\alpha_1, \alpha_2, k, \varphi, \varepsilon_{OA}, l_1, l_2, \omega_{OA}, m_1, m_2, m_3, g, \mu, F, x_A, y_A, x_B, y_B, x_{G1}, y_{G1}, x_{G2}, y_{G2}, x_O, y_O, x_M, y_M, x_N, y_N, l_0, MN, \Delta l, F_{dh}, \varepsilon_{AB}, v_B, a_B, a_{G1x}, a_{G1y}, a_{G2x}, a_{G2y}, Sys\_dynamic, X_A, Y_A, X_O, Y_O, X_B, Y_B, M, N_B, F_{ms}, n, XX_O, XX_A, XX_B, YY_O, YY_A, YY_B, MM, NN_B, FF_{ms}, i, RR_O, RR_A, RR_B, J_1, J_2, AG_2, AG_1;$

*#if not is({args}, set(numeric))* **then return** *('procname')('args')* **end if**;

$\alpha_1 := x1; \alpha_2 := x2; k := x3;$

$l_1 := 0.175; l_2 := 0.58; \omega_{OA} := 3.1416; m_1 := 41.5147; m_2 := 7.656875; m_3 := 9.8996; g := 9.81;$
$\mu := 0.3;\ AG_2 := 0.3165732; AG_1 := 0.0019;$

$J_1 := 0.889678;$

$J_2 := 0.6204482568;$

$x_A := \varphi \rightarrow l_1 \cdot \cos(\varphi);$

$y_A := \varphi \rightarrow l_1 \cdot \sin(\varphi);$

$x_B := \varphi \rightarrow l_1 \cdot \cos(\varphi) + \left(l_2^2 - (l_1 \cdot \sin(\varphi))^2\right)^{\frac{1}{2}};$

$y_B := \varphi \rightarrow 0;$

$x_{G1} := \varphi \rightarrow AG_1 \cdot \cos(\varphi);$

$y_{G1} := \varphi \rightarrow AG_1 \cdot \sin(\varphi);$

$x_{G2} := \varphi \rightarrow x_A(\varphi) + AG_2 \cdot \sqrt{1 - \frac{l_1^2}{l_2^2} \cdot (\sin(\varphi))^2};$

$y_{G2} := \varphi \rightarrow \left(1 - \frac{AG_2}{l_2}\right) \cdot y_A(\varphi);$

$x_O := \varphi \rightarrow 0;$

$y_O := \varphi \rightarrow 0;$

$v_B := \varphi \rightarrow -\left(1 + \frac{l_1 \cdot \cos(\varphi)}{\left(l_2^2 - l_1^2 \cdot (\sin(\varphi))^2\right)^{\frac{1}{2}}}\right) \cdot \omega_{OA} \cdot l_1 \cdot \sin(\varphi);$

$x_M := \varphi \rightarrow \alpha_1 \cdot l_1 \cdot \cos(\varphi);$

$y_M := \varphi \rightarrow \alpha_1 \cdot l_1 \cdot \sin(\varphi);$

$x_N := \varphi \rightarrow l_1 \cdot \cos(\varphi) + \alpha_2 \cdot \sqrt{l_2^2 - l_1^2 \cdot (\sin(\varphi))^2};$

$y_N := \varphi \rightarrow (1 - \alpha_2) \cdot l_1 \cdot \sin(\varphi);$

$$l_0 := \varphi \to \left(1-\alpha_1\right)\cdot l_1 + \alpha_2 \cdot l_2;$$

$$MN := \varphi \to 10^{-10} + \sqrt{\left(x_M(\varphi) - x_N(\varphi)\right)^2 + \left(y_M(\varphi) - y_N(\varphi)\right)^2};$$

$$\Delta l := \varphi \to MN(\varphi) - l_0(\varphi);$$

$$F_{dh} := \varphi \to k \cdot \Delta l(\varphi);$$

$$\varepsilon_{OA} := \varphi \to 0;$$

$$\varepsilon_{AB} := \varphi \to \frac{l_2^2 - l_1^2}{\left(l_2^2 - l_1^2 \cdot (\sin(\varphi))^2\right)^{\frac{3}{2}}} \cdot \left(\omega_{OA}\right)^2 \cdot l_1 \cdot \sin(\varphi) - \frac{l_1 \cdot \cos(\varphi)}{\left(l_2^2 - l_1^2 \cdot (\sin(\varphi))^2\right)^{\frac{1}{2}}} \cdot \varepsilon_{OA}(\varphi);$$

$$a_B := \varphi \to -\varepsilon_{OA}(\varphi) \cdot l_1 \cdot \sin(\varphi) \cdot \left(1 + \frac{l_1 \cdot \cos(\varphi)}{\left(l_2^2 - l_1^2 \cdot (\sin(\varphi))^2\right)^{\frac{1}{2}}}\right) + l_1 \cdot \omega_{OA}^{\ 2} \cdot \left(\frac{l_2^2 - l_1^2}{\left(l_2^2 - l_1^2 \cdot (\sin(\varphi))^2\right)^{\frac{3}{2}}} \cdot l_1 \cdot (\sin(\varphi))^2 - \cos(\varphi) - \frac{l_1 \cdot (\cos(\varphi))^2}{\left(l_2^2 - l_1^2 \cdot (\sin(\varphi))^2\right)^{\frac{1}{2}}}\right);$$

$$a_{G1x} := \varphi \to -AG_1 \cdot \left(\varepsilon_{OA}(\varphi) \cdot \sin(\varphi) + \omega_{OA}^{\ 2} \cdot \cos(\varphi)\right);$$

$$a_{G1y} := \varphi \to AG_1 \cdot \left(\varepsilon_{OA}(\varphi) \cdot \cos(\varphi) - \omega_{OA}^{\ 2} \cdot \sin(\varphi)\right);$$

$$\#a_{G2x} := \varphi \to -l_1 \sin(\varphi) \left(1 + \frac{AG_2\, l_1 \cos(\varphi)}{\sqrt{1 - \frac{l_1^2 \sin(\varphi)^2}{l_2^2}}\ l_2}\right) \varepsilon_{OA}(\varphi) - l_1\, \omega_{OA}^{\ 2} \left(\cos(\varphi) + \frac{AG_2\, l_1^3 \sin(\varphi)^2 \cos(\varphi)^2}{\left(1 - \frac{l_1^2 \sin(\varphi)^2}{l_2^2}\right)^{3/2} l_2^4} + \frac{AG_2\, l_1 \cos(2\varphi)}{\sqrt{1 - \frac{l_1^2 \sin(\varphi)^2}{l_2^2}}\ l_2^2}\right);$$

$$a_{G2x} := \varphi \to -l_1 \left(\varepsilon_{OA}(\varphi)\right) \sin(\varphi) - l_1 \left(\omega_{OA}\right)^2 \cos(\varphi) - \frac{AG_2\, l_1^4 \sin(\varphi)^2 \left(\omega_{OA}\right)^2 \cos(\varphi)^2}{\left(1 - \frac{l_1^2 \sin(\varphi)^2}{l_2^2}\right)^{3/2} l_2^4} - \frac{AG_2\, l_1^2 \left(\omega_{OA}\right)^2 \cos(\varphi)^2}{\sqrt{1 - \frac{l_1^2 \sin(\varphi)^2}{l_2^2}}\ l_2^2} - \frac{AG_2\, l_1^2 \sin(\varphi) \left(\varepsilon_{OA}(\varphi)\right) \cos(\varphi)}{\sqrt{1 - \frac{l_1^2 \sin(\varphi)^2}{l_2^2}}\ l_2^2} + \frac{AG_2\, l_1^2 \sin(\varphi)^2 \left(\omega_{OA}\right)^2}{\sqrt{1 - \frac{l_1^2 \sin(\varphi)^2}{l_2^2}}\ l_2^2};$$

$$\#a_{G2y} := \varphi \to \frac{\left(-l_2 + AG_2\right) l_1 \left(\omega_{OA}^{\ 2} \sin(\varphi) - \varepsilon_{OA}(\varphi) \cos(\varphi)\right)}{l_2};$$

$a_{G2y} := \varphi \rightarrow \left(1 - \frac{AG_2}{l_2}\right) l_1 \left(\varepsilon_{OA}(\varphi)\right) \cos(\varphi) - \left(1 - \frac{AG_2}{l_2}\right) l_1 \left(\omega_{OA}\right)^2 \sin(\varphi);$

$n := 360;$

$XX_O := Matrix(n, 2) :$

$XX_A := Matrix(n, 2) :$

$YY_O := Matrix(n, 2) :$

$YY_A := Matrix(n, 2) :$

$MM := Matrix(n, 2) :$

$XX_B := Matrix(n, 2) :$

$YY_B := Matrix(n, 2) :$

$NN_B := Matrix(n, 2) :$

$FF_{ms} := Matrix(n, 2) :$

$RR_O := Matrix(n, 2) :$

$RR_A := Matrix(n, 2) :$

$RR_B := Matrix(n, 2) :$

$F := Matrix(n, 1) :$

**for** *i* **from** 271 **to** 360 **do**

$F[i] := -3233;$

**od**:

**for** *i* **from** 1 **by** 1 **to** *n* **do**

$\varphi := \frac{(i-1)\cdot\pi}{180};$

$Sys\_dynamic := \{$

$X_O + X_A + F_{dh}(\varphi)\cdot\frac{x_N(\varphi) - x_M(\varphi)}{MN(\varphi)} = m_1\cdot a_{G1x}(\varphi),$

$Y_O + Y_A - m_1\cdot g + F_{dh}(\varphi)\cdot\frac{y_N(\varphi) - y_M(\varphi)}{MN(\varphi)} = m_1\cdot a_{G1y}(\varphi),$

$M + \left(x_A(\varphi) - x_{G1}(\varphi)\right)\cdot Y_A - \left(y_A(\varphi) - y_{G1}(\varphi)\right)\cdot X_A + \left(x_O(\varphi) - x_{G1}(\varphi)\right)\cdot Y_O - \left(y_O(\varphi) - y_{G1}(\varphi)\right)\cdot X_O + \left(x_M(\varphi) - x_{G1}(\varphi)\right)\cdot F_{dh}(\varphi)\cdot\frac{y_N(\varphi) - y_M(\varphi)}{MN(\varphi)} - \left(y_M(\varphi) - y_{G1}(\varphi)\right)\cdot F_{dh}(\varphi)\cdot\frac{x_N(\varphi) - x_M(\varphi)}{MN(\varphi)} = J_1\cdot\varepsilon_{OA}(\varphi),$

$-X_A + X_B + F_{dh}(\varphi)\cdot\frac{x_M(\varphi) - x_N(\varphi)}{MN(\varphi)} = m_2\cdot a_{G2x}(\varphi),$

$-Y_A + Y_B - m_2\cdot g + F_{dh}(\varphi)\cdot\frac{y_M(\varphi) - y_N(\varphi)}{MN(\varphi)} = m_2\cdot a_{G2y}(\varphi),$

$\left(x_A(\varphi) - x_{G2}(\varphi)\right)\cdot\left(-Y_A\right) - \left(y_A(\varphi) - y_{G2}(\varphi)\right)\cdot\left(-X_A\right) + \left(x_B(\varphi) - x_{G2}(\varphi)\right)\cdot Y_B - \left(y_B(\varphi)\right.$

$$-y_{G2}(\varphi))\cdot X_B+(x_N(\varphi)-x_{G2}(\varphi))\cdot F_{dh}(\varphi)\cdot\frac{y_M(\varphi)-y_N(\varphi)}{MN(\varphi)}-(y_N(\varphi)-y_{G2}(\varphi))\cdot F_{dh}(\varphi)$$
$$\cdot\frac{x_M(\varphi)-x_N(\varphi)}{MN(\varphi)}=J_2\cdot\varepsilon_{AB}(\varphi),$$
$$Y_B+m_3\cdot g=N_B,$$
$$-\mu\cdot|N_B|\cdot\frac{v_B(\varphi)}{|v_B(\varphi)|+10^{-6}}=F_{ms},$$
$$-X_B+F_{ms}+F(i)=m_3\cdot a_B(\varphi)\Big\}:$$

$fsolve(Sys\_dynamic, \{X_A, Y_A, X_O, Y_O, X_B, Y_B, M, N_B, F_{ms}\}) : assign(\%);$

$XX_O(i,1) := i; XX_A(i,1) := i; XX_B(i,1) = i; YY_O(i,1) := i; YY_A(i,1) := i; YY_B(i,1) = i; MM(i, 1) := i; NN_B(i,1) := i; FF_{ms}(i,1) := i; RR_O(i,1) := i; RR_A(i,1) := i; RR_B(i,1) := i;$

$XX_O(i,2) := X_O; XX_A(i,2) := X_A; XX_B(i,2) = X_B; YY_O(i,2) := Y_O; YY_A(i,2) := Y_A; YY_B(i,2) = Y_B; MM(i,2) := M; NN_B(i,2) := N_B; FF_{ms}(i,2) := F_{ms};$

$RR_O(i,2) := \sqrt{(X_O)^2+(Y_O)^2};$

$RR_A(i,2) := \sqrt{(X_A)^2+(Y_A)^2};$

$RR_B(i,2) := \sqrt{(X_B)^2+(Y_B)^2};$

$unassign('X_O','X_A','X_B','Y_O','Y_A','Y_B','M','N_B','F_{ms}');$

**od**:

**return** $\left(\frac{\mathrm{abs}(MM[1,2])+\mathrm{abs}(MM[360,2])}{2}+add(\mathrm{abs}(MM[i,2]), i=2..359)\right)\cdot\frac{\pi}{180};$

**end proc**:

*#Φ2=Moment*

*Φ2* :=**proc**(*x1*, *x2*, *x3*)

**local** $\alpha_1, \alpha_2, k, \varphi, \varepsilon_{OA}, l_1, l_2, \omega_{OA}, m_1, m_2, m_3, g, \mu, F, x_A, y_A, x_B, y_B, x_{G1}, y_{G1}, x_{G2}, y_{G2}, x_O, y_O, x_M, y_M, x_N, y_N, l_0, MN, \Delta l, F_{dh}, \varepsilon_{AB}, v_B, a_B, a_{G1x}, a_{G1y}, a_{G2x}, a_{G2y}, Sys\_dynamic, X_A, Y_A, X_O, Y_O, X_B, Y_B, M, N_B, F_{ms}, n, XX_O, XX_A, XX_B, YY_O, YY_A, YY_B, MM, NN_B, FF_{ms}, i, RR_O, RR_A, RR_B, J_1, J_2, AG_2, AG_1;$

*#if not is*( {*args*}, *set*(*numeric*)) **then return** ('*procname*')('*args*') **end if**;

$\alpha_1 := x1; \alpha_2 := x2; k := x3;$

$l_1 := 0.175; l_2 := 0.58; \omega_{OA} := 3.1416; m_1 := 41.5147; m_2 := 7.656875; m_3 := 9.8996; g := 9.81; \mu := 0.3;\ AG_2 := 0.3165732; AG_1 := 0.0019;$

$J_1 := 0.889678;$

$J_2 := 0.6204482568;$

$x_A := \varphi \rightarrow l_1\cdot\cos(\varphi);$

$y_A := \varphi \rightarrow l_1\cdot\sin(\varphi);$

$$x_B := \varphi \to l_1 \cdot \cos(\varphi) + \left(l_2^{\,2} - \left(l_1 \cdot \sin(\varphi)\right)^2\right)^{\frac{1}{2}};$$

$$y_B := \varphi \to 0;$$

$$x_{G1} := \varphi \to AG_1 \cdot \cos(\varphi);$$

$$y_{G1} := \varphi \to AG_1 \cdot \sin(\varphi);$$

$$x_{G2} := \varphi \to x_A(\varphi) + AG_2 \cdot \sqrt{1 - \frac{l_1^{\,2}}{l_2^{\,2}} \cdot \left(\sin(\varphi)\right)^2};$$

$$y_{G2} := \varphi \to \left(1 - \frac{AG_2}{l_2}\right) \cdot y_A(\varphi);$$

$$x_{\mathrm{O}} := \varphi \to 0;$$

$$y_{\mathrm{O}} := \varphi \to 0;$$

$$v_B := \varphi \to -\left(1 + \frac{l_1 \cdot \cos(\varphi)}{\left(l_2^{\,2} - l_1^{\,2} \cdot \left(\sin(\varphi)\right)^2\right)^{\frac{1}{2}}}\right) \cdot \omega_{OA} \cdot l_1 \cdot \sin(\varphi);$$

$$x_M := \varphi \to \alpha_1 \cdot l_1 \cdot \cos(\varphi);$$

$$y_M := \varphi \to \alpha_1 \cdot l_1 \cdot \sin(\varphi);$$

$$x_N := \varphi \to l_1 \cdot \cos(\varphi) + \alpha_2 \cdot \sqrt{l_2^{\,2} - l_1^{\,2} \cdot \left(\sin(\varphi)\right)^2};$$

$$y_N := \varphi \to \left(1 - \alpha_2\right) \cdot l_1 \cdot \sin(\varphi);$$

$$l_0 := \varphi \to \left(1 - \alpha_1\right) \cdot l_1 + \alpha_2 \cdot l_2;$$

$$MN := \varphi \to 10^{-10} + \sqrt{\left(x_M(\varphi) - x_N(\varphi)\right)^2 + \left(y_M(\varphi) - y_N(\varphi)\right)^2};$$

$$\Delta l := \varphi \to MN(\varphi) - l_0(\varphi);$$

$$F_{dh} := \varphi \to k \cdot \Delta l(\varphi);$$

$$\varepsilon_{OA} := \varphi \to 0;$$

$$\varepsilon_{AB} := \varphi \to \frac{l_2^{\,2} - l_1^{\,2}}{\left(l_2^{\,2} - l_1^{\,2} \cdot \left(\sin(\varphi)\right)^2\right)^{\frac{3}{2}}} \cdot \left(\omega_{OA}\right)^2 \cdot l_1 \cdot \sin(\varphi) - \frac{l_1 \cdot \cos(\varphi)}{\left(l_2^{\,2} - l_1^{\,2} \cdot \left(\sin(\varphi)\right)^2\right)^{\frac{1}{2}}} \cdot \varepsilon_{OA}(\varphi);$$

$$a_B := \varphi \to -\varepsilon_{OA}(\varphi) \cdot l_1 \cdot \sin(\varphi) \cdot \left(1 + \frac{l_1 \cdot \cos(\varphi)}{\left(l_2^{\,2} - l_1^{\,2} \cdot \left(\sin(\varphi)\right)^2\right)^{\frac{1}{2}}}\right) + l_1 \cdot \omega_{OA}^{\;\;2} \cdot \left(\frac{l_2^{\,2} - l_1^{\,2}}{\left(l_2^{\,2} - l_1^{\,2} \cdot \left(\sin(\varphi)\right)^2\right)^{\frac{3}{2}}} \cdot l_1 \cdot \left(\sin(\varphi)\right)^2 - \cos(\varphi) - \frac{l_1 \cdot \left(\cos(\varphi)\right)^2}{\left(l_2^{\,2} - l_1^{\,2} \cdot \left(\sin(\varphi)\right)^2\right)^{\frac{1}{2}}}\right);$$

$$a_{G1x} := \varphi \to -AG_1 \cdot \left(\varepsilon_{OA}(\varphi) \cdot \sin(\varphi) + \omega_{OA}^{\;\;2} \cdot \cos(\varphi)\right);$$

$$a_{G1y} := \varphi \to AG_1 \cdot \left(\varepsilon_{OA}(\varphi) \cdot \cos(\varphi) - \omega_{OA}^{\;\;2} \cdot \sin(\varphi)\right);$$

$$\#a_{G2x} := \varphi \rightarrow -l_1 \sin(\varphi) \left(1 + \frac{AG_2 \, l_1 \cos(\varphi)}{\sqrt{1 - \frac{l_1^2 \sin(\varphi)^2}{l_2^2}} \; l_2^2}\right) \varepsilon_{OA}(\varphi) - l_1 \, \omega_{OA}^{\;2} \left(\cos(\varphi) + \frac{AG_2 \, l_1^3 \sin(\varphi)^2 \cos(\varphi)^2}{\left(1 - \frac{l_1^2 \sin(\varphi)^2}{l_2^2}\right)^{3/2} l_2^4} + \frac{AG_2 \, l_1 \cos(2\,\varphi)}{\sqrt{1 - \frac{l_1^2 \sin(\varphi)^2}{l_2^2}} \; l_2^2}\right);$$

$$a_{G2x} := \varphi \rightarrow -l_1 \left(\varepsilon_{OA}(\varphi)\right) \sin(\varphi) - l_1 \left(\omega_{OA}\right)^2 \cos(\varphi) - \frac{AG_2 \, l_1^4 \sin(\varphi)^2 \left(\omega_{OA}\right)^2 \cos(\varphi)^2}{\left(1 - \frac{l_1^2 \sin(\varphi)^2}{l_2^2}\right)^{3/2} l_2^4} - \frac{AG_2 \, l_1^2 \left(\omega_{OA}\right)^2 \cos(\varphi)^2}{\sqrt{1 - \frac{l_1^2 \sin(\varphi)^2}{l_2^2}} \; l_2^2} - \frac{AG_2 \, l_1^2 \sin(\varphi) \left(\varepsilon_{OA}(\varphi)\right) \cos(\varphi)}{\sqrt{1 - \frac{l_1^2 \sin(\varphi)^2}{l_2^2}} \; l_2^2} + \frac{AG_2 \, l_1^2 \sin(\varphi)^2 \left(\omega_{OA}\right)^2}{\sqrt{1 - \frac{l_1^2 \sin(\varphi)^2}{l_2^2}} \; l_2^2};$$

$$\#a_{G2y} := \varphi \rightarrow \frac{\left(-l_2 + AG_2\right) l_1 \left(\omega_{OA}^{\;2} \sin(\varphi) - \varepsilon_{OA}(\varphi) \cos(\varphi)\right)}{l_2};$$

$$a_{G2y} := \varphi \rightarrow \left(1 - \frac{AG_2}{l_2}\right) l_1 \left(\varepsilon_{OA}(\varphi)\right) \cos(\varphi) - \left(1 - \frac{AG_2}{l_2}\right) l_1 \left(\omega_{OA}\right)^2 \sin(\varphi);$$

$n := 360;$
$XX_{\mathrm{O}} := Matrix(n, 2):$
$XX_A := Matrix(n, 2):$
$YY_{\mathrm{O}} := Matrix(n, 2):$
$YY_A := Matrix(n, 2):$
$MM := Matrix(n, 2):$
$XX_B := Matrix(n, 2):$
$YY_B := Matrix(n, 2):$
$NN_B := Matrix(n, 2):$
$FF_{ms} := Matrix(n, 2):$
$RR_{\mathrm{O}} := Matrix(n, 2):$
$RR_A := Matrix(n, 2):$
$RR_B := Matrix(n, 2):$
$F := Matrix(n, 1):$
**for** *i* **from** 271 **to** 360 **do**
$F[i] := -3233;$
**od**:

**for** $i$ **from** 1 **by** 1 **to** $n$ **do**

$\varphi := \frac{(i-1)\cdot\pi}{180}$;

$$Sys\_dynamic := \Big\{$$

$$X_O + X_A + F_{dh}(\varphi)\cdot\frac{x_N(\varphi) - x_M(\varphi)}{MN(\varphi)} = m_1\cdot a_{G1x}(\varphi),$$

$$Y_O + Y_A - m_1\cdot g + F_{dh}(\varphi)\cdot\frac{y_N(\varphi) - y_M(\varphi)}{MN(\varphi)} = m_1\cdot a_{G1y}(\varphi),$$

$$M + \left(x_A(\varphi) - x_{G1}(\varphi)\right)\cdot Y_A - \left(y_A(\varphi) - y_{G1}(\varphi)\right)\cdot X_A + \left(x_O(\varphi) - x_{G1}(\varphi)\right)\cdot Y_O - \left(y_O(\varphi) - y_{G1}(\varphi)\right)\cdot X_O + \left(x_M(\varphi) - x_{G1}(\varphi)\right)\cdot F_{dh}(\varphi)\cdot\frac{y_N(\varphi) - y_M(\varphi)}{MN(\varphi)} - \left(y_M(\varphi) - y_{G1}(\varphi)\right)\cdot F_{dh}(\varphi)\cdot\frac{x_N(\varphi) - x_M(\varphi)}{MN(\varphi)} = J_1\cdot\varepsilon_{OA}(\varphi),$$

$$-X_A + X_B + F_{dh}(\varphi)\cdot\frac{x_M(\varphi) - x_N(\varphi)}{MN(\varphi)} = m_2\cdot a_{G2x}(\varphi),$$

$$-Y_A + Y_B - m_2\cdot g + F_{dh}(\varphi)\cdot\frac{y_M(\varphi) - y_N(\varphi)}{MN(\varphi)} = m_2\cdot a_{G2y}(\varphi),$$

$$\left(x_A(\varphi) - x_{G2}(\varphi)\right)\cdot\left(-Y_A\right) - \left(y_A(\varphi) - y_{G2}(\varphi)\right)\cdot\left(-X_A\right) + \left(x_B(\varphi) - x_{G2}(\varphi)\right)\cdot Y_B - \left(y_B(\varphi) - y_{G2}(\varphi)\right)\cdot X_B + \left(x_N(\varphi) - x_{G2}(\varphi)\right)\cdot F_{dh}(\varphi)\cdot\frac{y_M(\varphi) - y_N(\varphi)}{MN(\varphi)} - \left(y_N(\varphi) - y_{G2}(\varphi)\right)\cdot F_{dh}(\varphi)\cdot\frac{x_M(\varphi) - x_N(\varphi)}{MN(\varphi)} = J_2\cdot\varepsilon_{AB}(\varphi),$$

$$Y_B + m_3\cdot g = N_B,$$

$$-\mu\cdot|N_B|\cdot\frac{v_B(\varphi)}{|v_B(\varphi)| + 10^{-6}} = F_{ms},$$

$$-X_B + F_{ms} + F(i) = m_3\cdot a_B(\varphi)\Big\}:$$

$fsolve\left(Sys\_dynamic, \{X_A, Y_A, X_O, Y_O, X_B, Y_B, M, N_B, F_{ms}\}\right) : assign(\%);$

$XX_O(i, 1) := i; XX_A(i, 1) := i; XX_B(i, 1) = i; YY_O(i, 1) := i; YY_A(i, 1) := i; YY_B(i, 1) = i; MM(i, 1) := i; NN_B(i, 1) := i; FF_{ms}(i, 1) := i; RR_O(i, 1) := i; RR_A(i, 1) := i; RR_B(i, 1) := i;$

$XX_O(i, 2) := X_O; XX_A(i, 2) := X_A; XX_B(i, 2) = X_B; YY_O(i, 2) := Y_O; YY_A(i, 2) := Y_A; YY_B(i, 2) = Y_B; MM(i, 2) := M; NN_B(i, 2) := N_B; FF_{ms}(i, 2) := F_{ms};$

$RR_O(i, 2) := \sqrt{(X_O)^2 + (Y_O)^2};$

$RR_A(i, 2) := \sqrt{(X_A)^2 + (Y_A)^2};$

$RR_B(i, 2) := \sqrt{(X_B)^2 + (Y_B)^2};$
$unassign('X_O','X_A','X_B','Y_O','Y_A','Y_B','M','N_B','F_{ms}');$
**od**:
**return** max(|*MM*|);
**end proc**:

## Setting the range of parameter variations: (each task has its own)

$Lo\_1 := \langle 0, 0, 0\rangle; Up\_1 := \langle 1, 1, 20000\rangle;$
$Param\_1 := [[0, 1], [0, 1], [0, 20000]];$

$$Lo\_1 := \begin{bmatrix} 0 \\ 0 \\ 0 \end{bmatrix}$$

$$Up\_1 := \begin{bmatrix} 1 \\ 1 \\ 20000 \end{bmatrix}$$

$$Param\_1 := [[0, 1], [0, 1], [0, 20000]] \qquad \textbf{(1.1)}$$

$n := 2^{10};$
$Gen\_Par\_Uniform\_1 := UniforGeneration(Lo\_1, Up\_1, n);$
$Gen\_Par\_LPTau\_1 := LPTAU(Param\_1, 1, n);$

$$n := 1024$$

$Gen\_Par\_Uniform\_1 :=$

| | | |
|---|---|---|
| 0.395718860500000 | 0.193139816400000 | 448.483409200000 |
| 0.193139816400000 | 0.800187484500000 | 16852.4536900000 |
| 0.0224241704600000 | 0.842622684400000 | 7728.16614800000 |
| 0.800187484500000 | 0.996417214200000 | 14612.3258600000 |
| 0.427552056900000 | 0.694607189300000 | 18898.2670000000 |
| 0.842622684400000 | 0.730616292900000 | 9094.88794000000 |
| 0.412286285800000 | 0.396412723000000 | 12314.6413800000 |
| 0.996417214200000 | 0.210936428900000 | 7851.09185600000 |
| 0.386408307400000 | 0.454744397000000 | 4481.70089200000 |
| 0.694607189300000 | 0.329844591800000 | 11194.1064500000 |
| ⋮ | ⋮ | ⋮ |

1024 × 3 Matrix

$$Gen\_Par\_LPTau\_1 := \begin{bmatrix} 0.5000000000 & 0.5000000000 & 10000. \\ 0.2500000000 & 0.7500000000 & 5000. \\ 0.7500000000 & 0.2500000000 & 15000. \\ 0.1250000000 & 0.6250000000 & 17500. \\ 0.6250000000 & 0.1250000000 & 7500. \\ 0.3750000000 & 0.3750000000 & 12500. \\ 0.8750000000 & 0.8750000000 & 2500. \\ 0.06250000000 & 0.9375000000 & 13750. \\ 0.5625000000 & 0.4375000000 & 3750. \\ 0.3125000000 & 0.1875000000 & 18750. \\ \vdots & \vdots & \vdots \end{bmatrix} \quad (1.2)$$

1024 × 3 Matrix

*plots*:-*pointplot*( {*seq*( [*Gen_Par_Uniform_1*[*k*, 2], *Gen_Par_Uniform_1*[*k*, 3]], *k* = 1 .. *n*) });
*plots*:-*pointplot*( {*seq*( [*Gen_Par_LPTau_1*[*k*, 2], *Gen_Par_LPTau_1*[*k*, 3]], *k* = 1 .. *n*) });

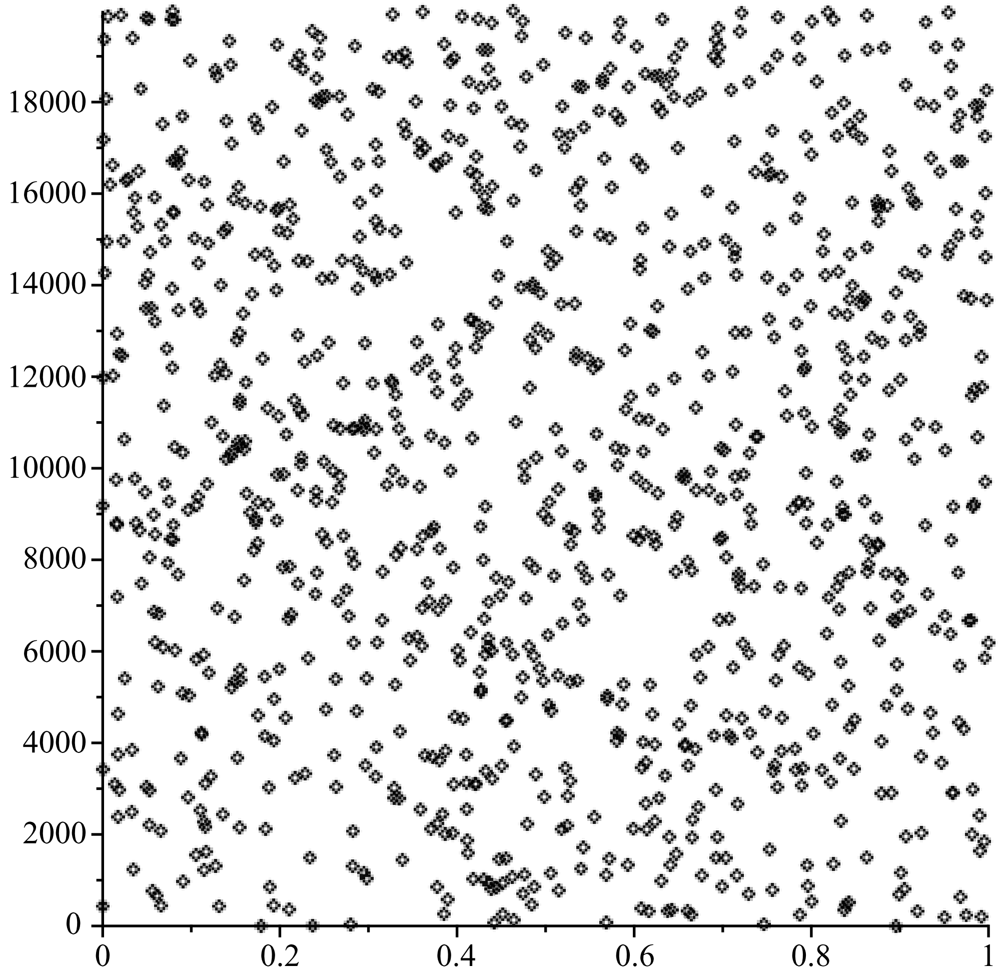

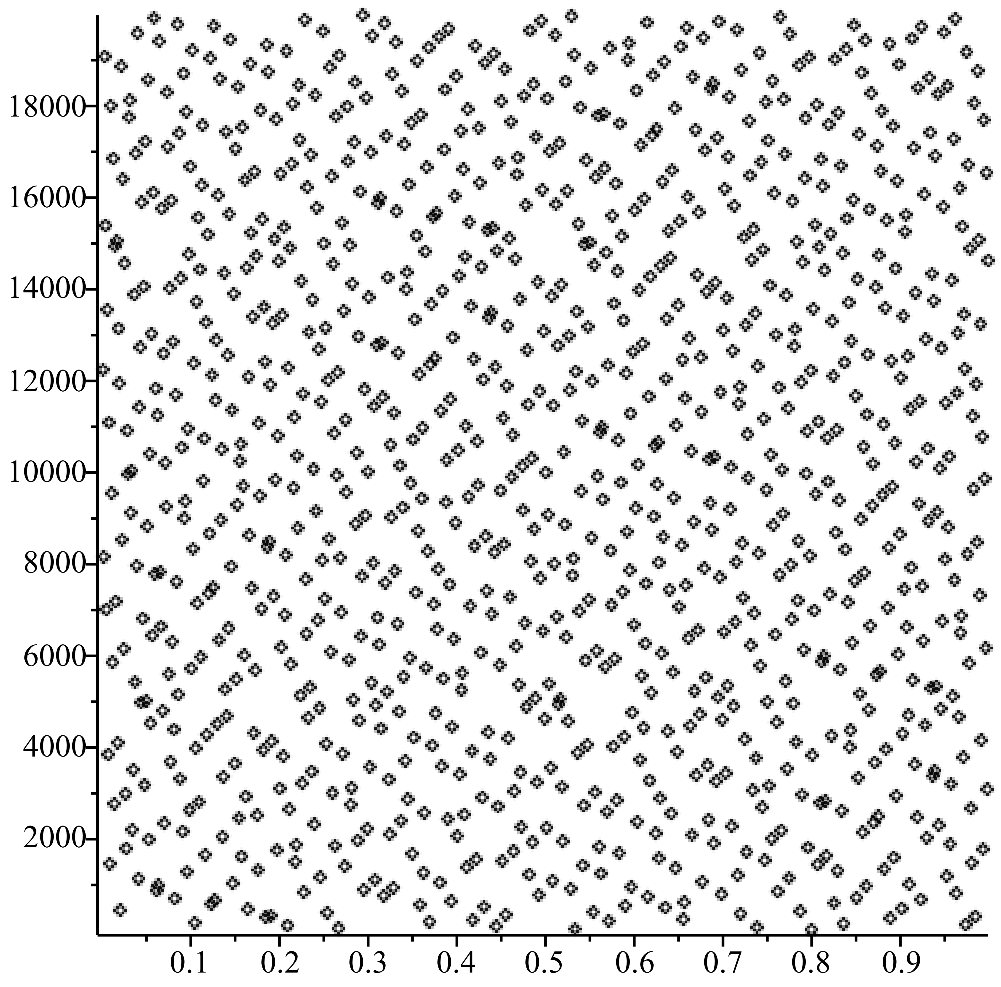


*Gen_Par_1* := *AddOrder*(⟨*Gen_Par_Uniform_1*, *Gen_Par_LPTau_1*⟩, 0)

*Gen_Par_1* := **(1.3)**

| | | | |
|---|---|---|---|
| 1 | 0.395718860500000 | 0.193139816400000 | 448.483409200000 |
| 2 | 0.193139816400000 | 0.800187484500000 | 16852.4536900000 |
| 3 | 0.0224241704600000 | 0.842622684400000 | 7728.16614800000 |
| 4 | 0.800187484500000 | 0.996417214200000 | 14612.3258600000 |
| 5 | 0.427552056900000 | 0.694607189300000 | 18898.2670000000 |
| 6 | 0.842622684400000 | 0.730616292900000 | 9094.88794000000 |
| 7 | 0.412286285800000 | 0.396412723000000 | 12314.6413800000 |
| 8 | 0.996417214200000 | 0.210936428900000 | 7851.09185600000 |
| 9 | 0.386408307400000 | 0.454744397000000 | 4481.70089200000 |
| 10 | 0.694607189300000 | 0.329844591800000 | 11194.1064500000 |
| ⋮ | ⋮ | ⋮ | ⋮ |

2048 × 4 Matrix

```
plots:-pointplot({seq([Gen_Par_1[k, 2], Gen_Par_1[k, 3]], k = 1 ..2· n)});
plots:-pointplot({seq([Gen_Par_1[k, 3], Gen_Par_1[k, 4]], k = 1 ..2· n)});
plots:-pointplot({seq([Gen_Par_1[k, 2], Gen_Par_1[k, 4]], k = 1 ..2· n)});
```

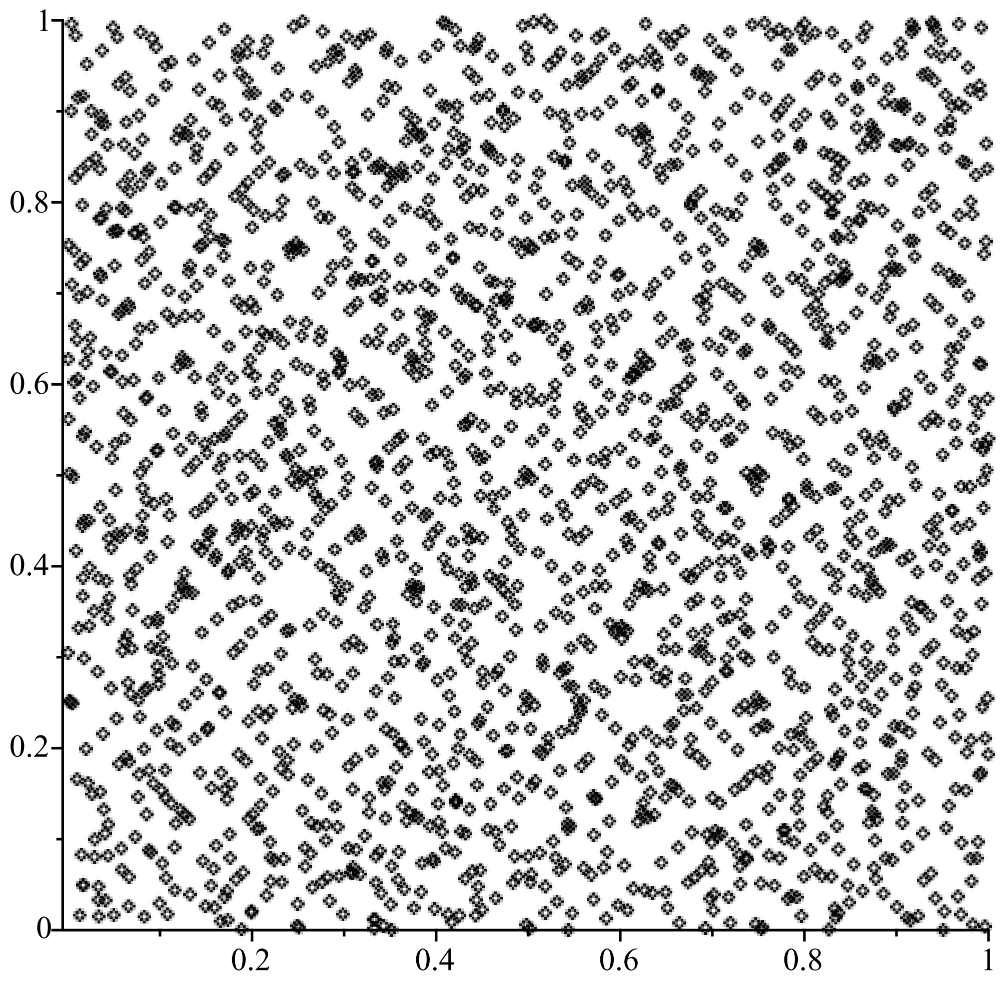

1
0.8
0.6
0.4
0.2
0
0.2
0.4
0.6
0.8
1

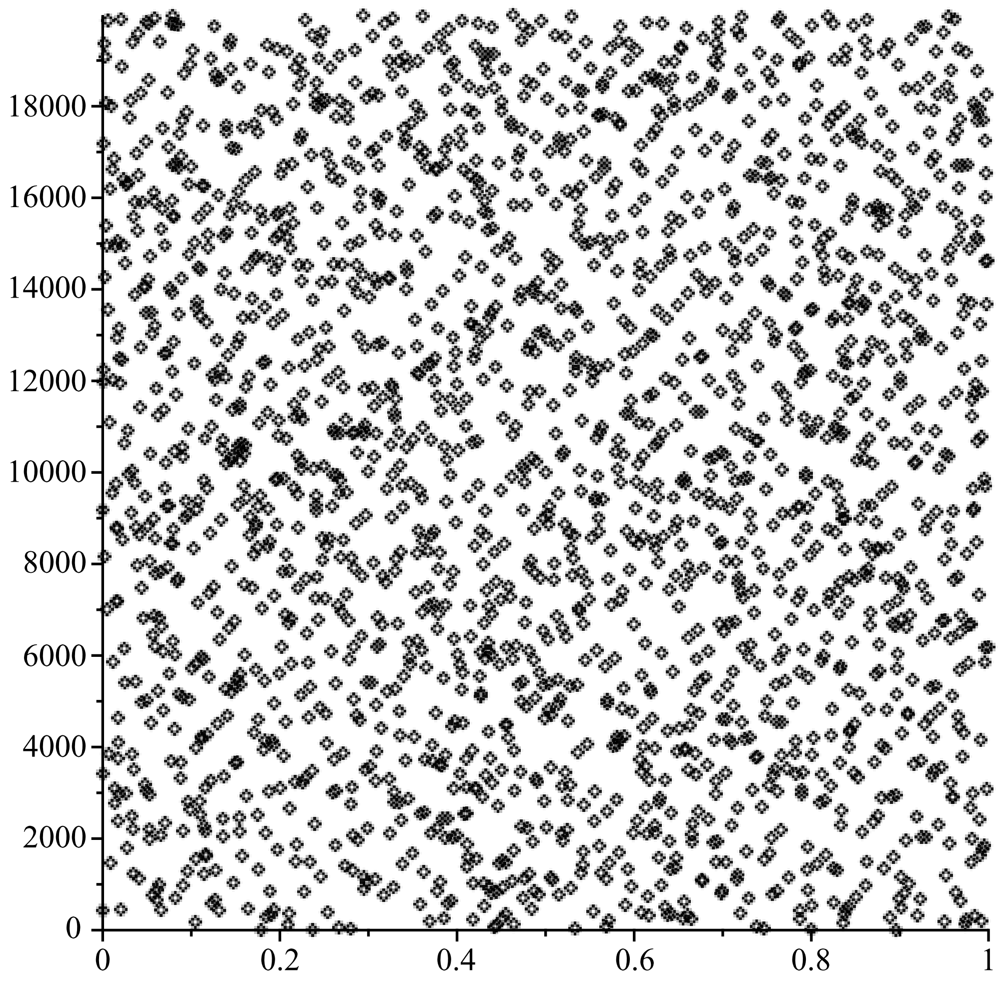

18000
16000
14000
12000
10000
8000
6000
4000
2000
0
0
0.2
0.4
0.6
0.8
1

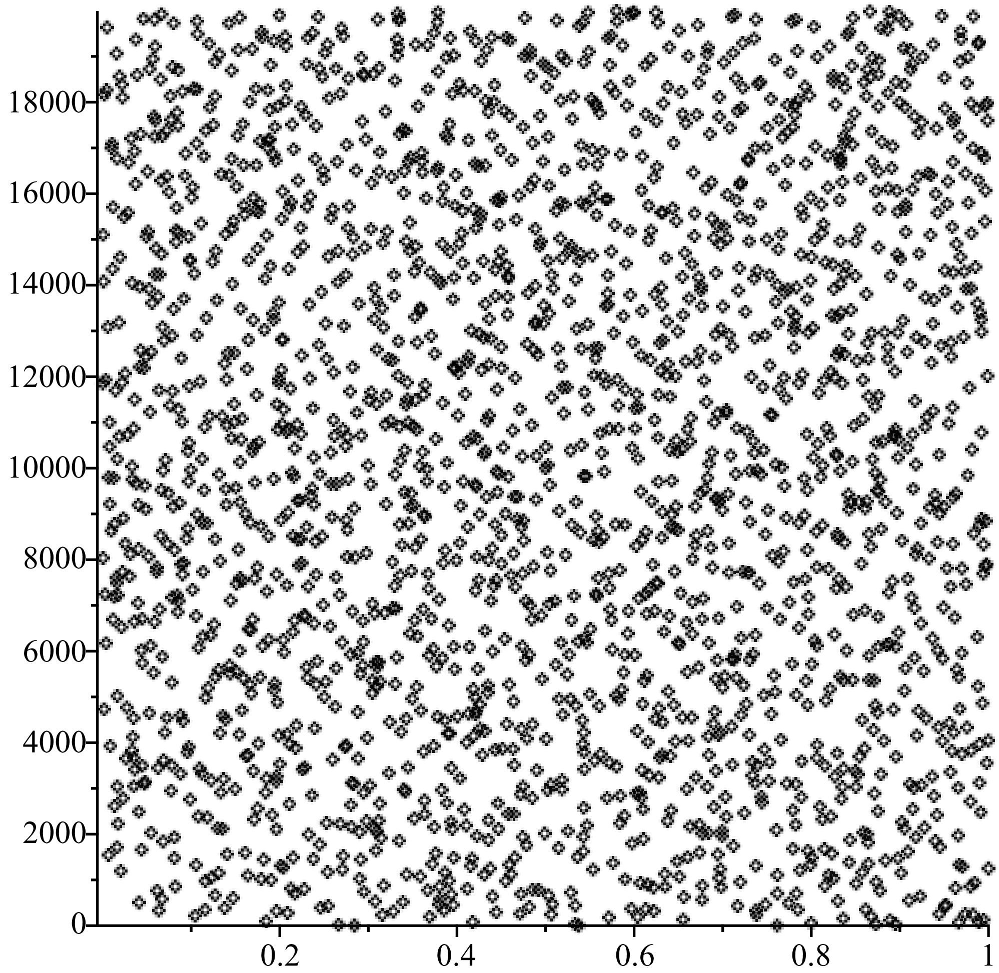


*plots*:-*pointplot3d*( *Gen_Par_1*( .., 2 ..4 ), *color* = "orange", *symbol* = *solidcircle* )

**#Full table (parameters, functional limitations and criteria)**
$TFULL := FULLTABLE(Gen\_Par\_1, 0);$

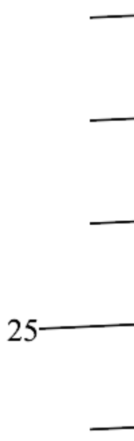

*TFULL* :=

| $i$ | $A_i$ | $\alpha 1$ | $\alpha 2$ | $\alpha 3$ | $f1$ | [illegible] |
|---|---|---|---|---|---|---|
| 1 | *A1* | 0.395718860500000 | 0.193139816400000 | 448.483409200000 | 3691.304991 | 973.4 |
| 2 | *A2* | 0.193139816400000 | 0.800187484500000 | 16852.4536900000 | 4771.529157 | 903.4 |
| 3 | *A3* | 0.0224241704600000 | 0.842622684400000 | 7728.16614800000 | 3231.819571 | 655.5 |
| 4 | *A4* | 0.800187484500000 | 0.996417214200000 | 14612.3258600000 | 3254.210041 | 936.4 |
| 5 | *A5* | 0.427552056900000 | 0.694607189300000 | 18898.2670000000 | 3801.753069 | 604.4 |
| 6 | *A6* | 0.842622684400000 | 0.730616292900000 | 9094.88794000000 | 3425.469531 | 963.4 |
| 7 | *A7* | 0.412286285800000 | 0.396412723000000 | 12314.6413800000 | 3231.846287 | 737.1 |
| 8 | *A8* | 0.996417214200000 | 0.210936428900000 | 7851.09185600000 | 3713.498124 | 980.4 |
| 9 | *A9* | 0.386408307400000 | 0.454744397000000 | 4481.70089200000 | 3314.989941 | 871.9 |
| ⋮ | ⋮ | ⋮ | ⋮ | ⋮ | ⋮ | |

$TS\_FULL := \langle TFULL(..,1) | TFULL(..,3..NParameters+2)\rangle;$

*TS_FULL* :=

| $i$ | $\alpha 1$ | $\alpha 2$ | $\alpha 3$ |
|---|---|---|---|
| 1 | 0.395718860500000 | 0.193139816400000 | 448.483409200000 |
| 2 | 0.193139816400000 | 0.800187484500000 | 16852.4536900000 |
| 3 | 0.0224241704600000 | 0.842622684400000 | 7728.16614800000 |
| 4 | 0.800187484500000 | 0.996417214200000 | 14612.3258600000 |
| 5 | 0.427552056900000 | 0.694607189300000 | 18898.2670000000 |
| 6 | 0.842622684400000 | 0.730616292900000 | 9094.88794000000 |
| 7 | 0.412286285800000 | 0.396412723000000 | 12314.6413800000 |
| 8 | 0.996417214200000 | 0.210936428900000 | 7851.09185600000 |
| 9 | 0.386408307400000 | 0.454744397000000 | 4481.70089200000 |
| ⋮ | ⋮ | ⋮ | ⋮ |

**(1.5)**

2049 × 4 Matrix

$TC\_FULL := \langle TFULL(..,2) | TFULL(..,NParameters+NFunctional+3) | TFULL(..,2) | TFULL(..,NParameters+NFunctional+4)\rangle;$

$$TC\_FULL := \begin{bmatrix} A_i & \Phi 1 & A_i & \Phi 2 \\ A1 & 735.668219303607 & A1 & 641.2615116 \\ A2 & 1586.93781108162 & A2 & 449.0553845 \\ A3 & 1307.59768973943 & A3 & 467.8953264 \\ A4 & 756.493935581703 & A4 & 622.6915336 \\ A5 & 1185.53963516141 & A5 & 476.1737966 \\ A6 & 735.985570826091 & A6 & 635.8111890 \\ A7 & 1063.75323204138 & A7 & 539.7509235 \\ A8 & 733.343785616706 & A8 & 644.0897238 \\ A9 & 846.481900412585 & A9 & 597.4363948 \\ \vdots & \vdots & \vdots & \vdots \end{bmatrix} \quad (1.6)$$

2049 × 4 Matrix

$RB\_FULL := \langle TFULL(.., 1) | TFULL(.., NParameters + 3 .. NParameters + NFunctional + 2) \rangle;$

$$RB\_FULL := \begin{bmatrix} i & f1 & f2 \\ 1 & 3691.304991 & 973.4706938 \\ 2 & 4771.529157 & 903.4456005 \\ 3 & 3231.819571 & 655.5010550 \\ 4 & 3254.210041 & 936.4264314 \\ 5 & 3801.753069 & 604.4878722 \\ 6 & 3425.469531 & 963.4534082 \\ 7 & 3231.846287 & 737.1896446 \\ 8 & 3713.498124 & 980.4574597 \\ 9 & 3314.989941 & 871.9687089 \\ \vdots & \vdots & \vdots \end{bmatrix} \quad (1.7)$$

2049 × 3 Matrix

$File1 := cat(currentdir(\,),$ "\\Excel\\TFULL.xlsx"$);$

$File1 :=$ **(1.8)**

"D:\GGDR\BAO CHI\BAO QUOC TE\Toi uu hoa su dung lo xo TQCT\Excel\TFULL.xlsx"

$XuatCach1 := Export(File1, TFULL);$

$XuatCach1 := (\,)$ **(1.9)**

**#Table of parameter vectors satisfying functional constraints: (each task has its own conditions)**

$F\_1 := Functional(TFULL, 1, 1);$

$F\_1 :=$ (1.10)

| i | α1 | α2 | α3 |
|---|---|---|---|
| 3 | 0.0224241704600000 | 0.842622684400000 | 7728.16614800000 |
| 7 | 0.412286285800000 | 0.396412723000000 | 12314.6413800000 |
| 28 | 0.0786061183300000 | 0.819571010900000 | 7174.94657400000 |
| 32 | 0.430137169300000 | 0.868571906600000 | 12852.2910600000 |
| 36 | 0.497201520400000 | 0.970011783000000 | 16704.1993400000 |
| 38 | 0.363627416900000 | 0.648830381600000 | 8936.47981000000 |
| 43 | 0.0361152253400000 | 0.894868300600000 | 6651.93083000000 |
| 45 | 0.450144918100000 | 0.431192847400000 | 15934.7384900000 |
| 51 | 0.346921332400000 | 0.927663025800000 | 14744.7918300000 |
| ⋮ | ⋮ | ⋮ | ⋮ |

256 × 4 Matrix

## #Functional failure table (with soft constraints → calculate criterion vectors): (each task has its own conditions)

*Functional*( *TFULL*, 23, 0 );

(1.11)

| $A_i$ | f1 | f2 | Φ1 | Φ2 |
|---|---|---|---|---|
| A1 | 3691.304991 | 973.4706938 | 735.668219303607 | 641.2615116 |
| A2 | 4771.529157 | 903.4456005 | 1586.93781108162 | 449.0553845 |
| A4 | 3254.210041 | 936.4264314 | 756.493935581703 | 622.6915336 |
| A5 | 3801.753069 | 604.4878722 | 1185.53963516141 | 476.1737966 |
| A6 | 3425.469531 | 963.4534082 | 735.985570826091 | 635.8111890 |
| A8 | 3713.498124 | 980.4574597 | 733.343785616706 | 644.0897238 |
| A9 | 3314.989941 | 871.9687089 | 846.481900412585 | 597.4363948 |
| A10 | 3257.130613 | 907.1060278 | 793.055464760536 | 611.3751033 |
| A11 | 3231.907431 | 913.3872422 | 776.057992655443 | 611.8691967 |
| ⋮ | ⋮ | ⋮ | ⋮ | ⋮ |

1794 × 5 Matrix

## #Create a table of initial test table based on the table of parameter vectors satisfying the functional constraints

*T1* := *Functional*( *TFULL*, 3, 1 );

$T1 :=$ (1.12)

| $A_i$ | Φ1 | $A_i$ | Φ2 |
|---|---|---|---|
| A3 | 1307.59768973943 | A3 | 467.8953264 |
| A7 | 1063.75323204138 | A7 | 539.7509235 |
| A28 | 1197.99941217317 | A28 | 490.3335407 |
| A32 | 1018.29539900978 | A32 | 517.1625333 |
| A36 | 1015.38455806179 | A36 | 511.5810656 |
| A38 | 989.370506233345 | A38 | 540.6445512 |
| A43 | 1202.08534609090 | A43 | 486.7017810 |
| A45 | 1101.32369198616 | A45 | 521.6433138 |
| A51 | 1184.55850327106 | A51 | 469.0855619 |
| ⋮ | ⋮ | ⋮ | ⋮ |

256 × 4 Matrix

## Sort the test table

*T1_sort* := *Test_Table_Sort*(*T1*);

$T1\_sort :=$ (1.13)

| $A_i$ | Φ1 | $A_i$ | Φ2 |
|---|---|---|---|
| A394 | 938.062428120827 | A1464 | 446.6870918 |
| A1641 | 944.0735444 | A1124 | 450.3182065 |
| A543 | 944.911350461758 | A1644 | 454.2354154 |
| A604 | 950.548450518865 | A931 | 454.9924670 |
| A1601 | 951.4842522 | A939 | 456.6120249 |
| A714 | 953.025676982415 | A1328 | 457.2295944 |
| A134 | 954.892466311227 | A652 | 457.3085081 |
| A101 | 955.631959913524 | A1106 | 461.7052699 |
| A1886 | 958.3807083 | A471 | 461.8492924 |
| ⋮ | ⋮ | ⋮ | ⋮ |

256 × 4 Matrix

## #Setting criteria constraints as a list

*ΦConstraints_1* := [1300, 530];

*ΦConstraints_1* := [1300, 530] (1.14)

## #Possible solutions

*feasiblesolve*(*ΦConstraints_1*, *T1*, *T1_sort*);

{*A1000*, *A1006*, *A1046*, *A1050*, *A1070*, *A1076*, *A1098*, *A1106*, *A1112*, *A1121*, *A1142*, *A1152*, (1.15)
*A1170*, *A1184*, *A1188*, *A1198*, *A1202*, *A1212*, *A1218*, *A1230*, *A1254*, *A1260*, *A1288*,
*A1300*, *A1334*, *A1338*, *A1345*, *A1350*, *A1366*, *A1372*, *A1394*, *A1406*, *A1412*, *A1422*,
*A1438*, *A1440*, *A1450*, *A1458*, *A1484*, *A1498*, *A1506*, *A1512*, *A1518*, *A1554*, *A1560*,

*A1566, A1596, A1602, A1616, A1626, A1634, A1644, A1662, A167, A1672, A1702, A1708, A1713, A1716, A173, A1738, A1750, A1764, A1793, A1802, A1814, A1820, A1842, A1848, A1854, A1868, A1870, A1876, A1890, A1900, A1904, A1914, A1926, A1937, A1954, A1960, A1978, A1988, A2016, A2022, A2026, A205, A219, A228, A264, A28, A283, A288, A310, A317, A32, A330, A342, A348, A354, A36, A383, A389, A395, A429, A43, A430, A45, A460, A461, A479, A509, A51, A514, A54, A551, A556, A559, A584, A585, A605, A61, A610, A612, A614, A619, A62, A623, A67, A691, A693, A699, A740, A745, A75, A771, A798, A80, A802, A808, A825, A832, A836, A849, A854, A87, A901, A907, A921, A927, A935, A940, A950, A973, A977, A993, A996}*

**#Table of feasible solutions**

*FEA_1 := FeasibleTable(ΦConstraints_1, T1);*

*FEA_1 :=*

| $A_i$ | *Φ1* | $A_i$ | *Φ2* |
|---|---|---|---|
| *A28* | 1197.99941217317 | *A28* | 490.3335407 |
| *A32* | 1018.29539900978 | *A32* | 517.1625333 |
| *A36* | 1015.38455806179 | *A36* | 511.5810656 |
| *A43* | 1202.08534609090 | *A43* | 486.7017810 |
| *A45* | 1101.32369198616 | *A45* | 521.6433138 |
| *A51* | 1184.55850327106 | *A51* | 469.0855619 |
| *A54* | 991.660950534555 | *A54* | 526.4757779 |
| *A61* | 1104.17765749996 | *A61* | 489.8151321 |
| *A62* | 1248.28259436695 | *A62* | 493.5247102 |
| ⋮ | ⋮ | ⋮ | ⋮ |

158 × 4 Matrix **(1.16)**

**#Table of Pareto-optimal solutions (criteria value)**

*PARETO_CR_1 := ParetoCriteria(ΦConstraints_1, T1);*

$$PARETO\_CR\_1 := \begin{bmatrix} A_i & \Phi 1 & A_i & \Phi 2 \\ A36 & 1015.38455806179 & A36 & 511.5810656 \\ A51 & 1184.55850327106 & A51 & 469.0855619 \\ A61 & 1104.17765749996 & A61 & 489.8151321 \\ A264 & 1070.96796950522 & A264 & 497.2035756 \\ A310 & 1120.89583821039 & A310 & 480.5663555 \\ A389 & 1050.74359473733 & A389 & 499.3378325 \\ A612 & 1008.20685363358 & A612 & 512.9468317 \\ A691 & 1110.85045777842 & A691 & 489.4396947 \\ A693 & 1093.17161124037 & A693 & 491.4834356 \\ \vdots & \vdots & \vdots & \vdots \end{bmatrix} \quad (1.17)$$

20 × 4 Matrix

## Table of Pareto-optimal solutions (parameter values)

$PARETO\_PAR\_1 := ParetoParameters(\Phi Constraints\_1, Gen\_Par\_1, T1);$

$$PARETO\_PAR\_1 := \quad (1.18)$$

$$\begin{bmatrix} Ai & \alpha 1 & \alpha 2 & \alpha 3 \\ 36 & 0.497201520400000 & 0.970011783000000 & 16704.1993400000 \\ 51 & 0.346921332400000 & 0.927663025800000 & 14744.7918300000 \\ 61 & 0.419107017600000 & 0.876097567600000 & 15664.6786600000 \\ 264 & 0.444411111900000 & 0.914502653400000 & 15858.3780200000 \\ 310 & 0.410668762400000 & 0.996707210900000 & 16013.2004300000 \\ 389 & 0.500292286800000 & 0.957439139900000 & 18783.8336000000 \\ 612 & 0.541902908200000 & 0.929139641700000 & 19744.4023000000 \\ 691 & 0.320494988800000 & 0.981526454600000 & 11585.1889200000 \\ 693 & 0.459645047800000 & 0.854668027400000 & 17695.1634400000 \\ \vdots & \vdots & \vdots & \vdots \end{bmatrix}$$

20 × 4 Matrix

## Criteria vs. Parameters II Graphs

$crvsvarII(1, 1, F\_1, T1, Gen\_Par\_1, FEA\_1, PARETO\_CR\_1);$

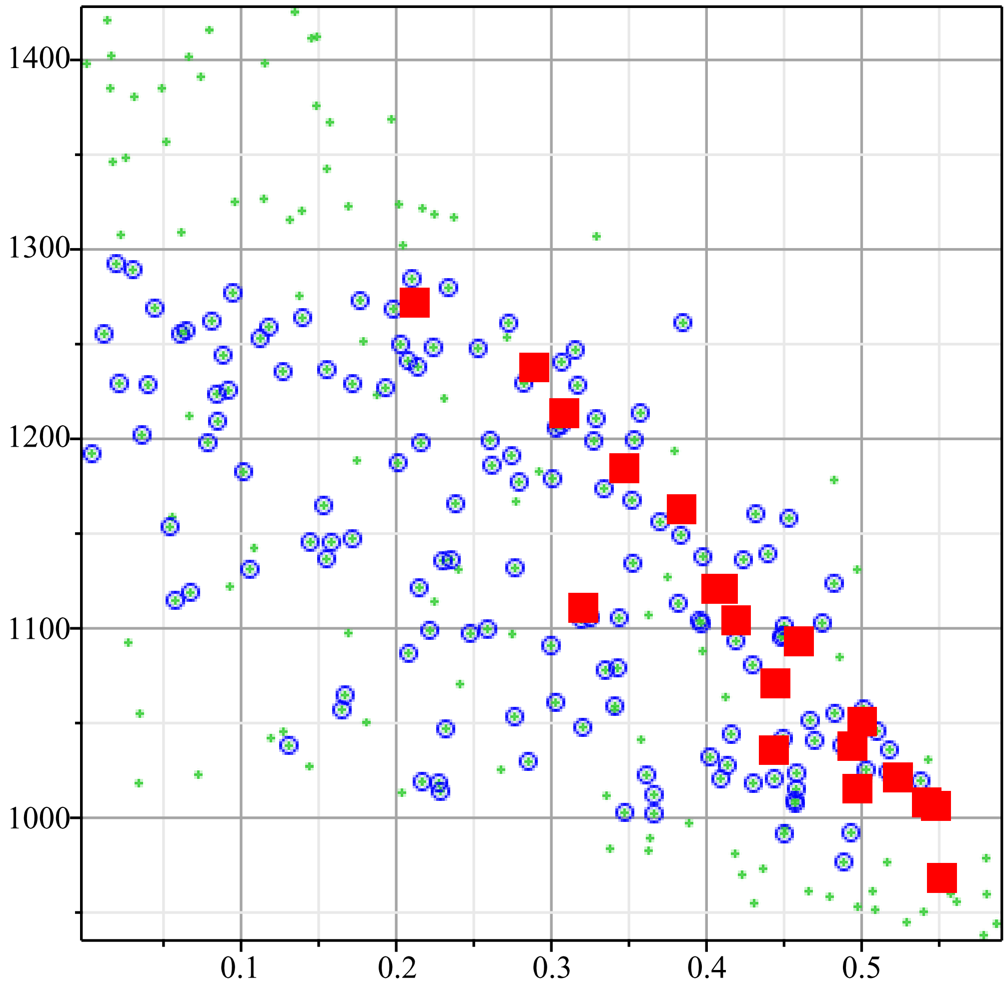


*crvsvarII*(1, 2, *F_1*, *T1*, *Gen_Par_1*, *FEA_1*, *PARETO_CR_1*);

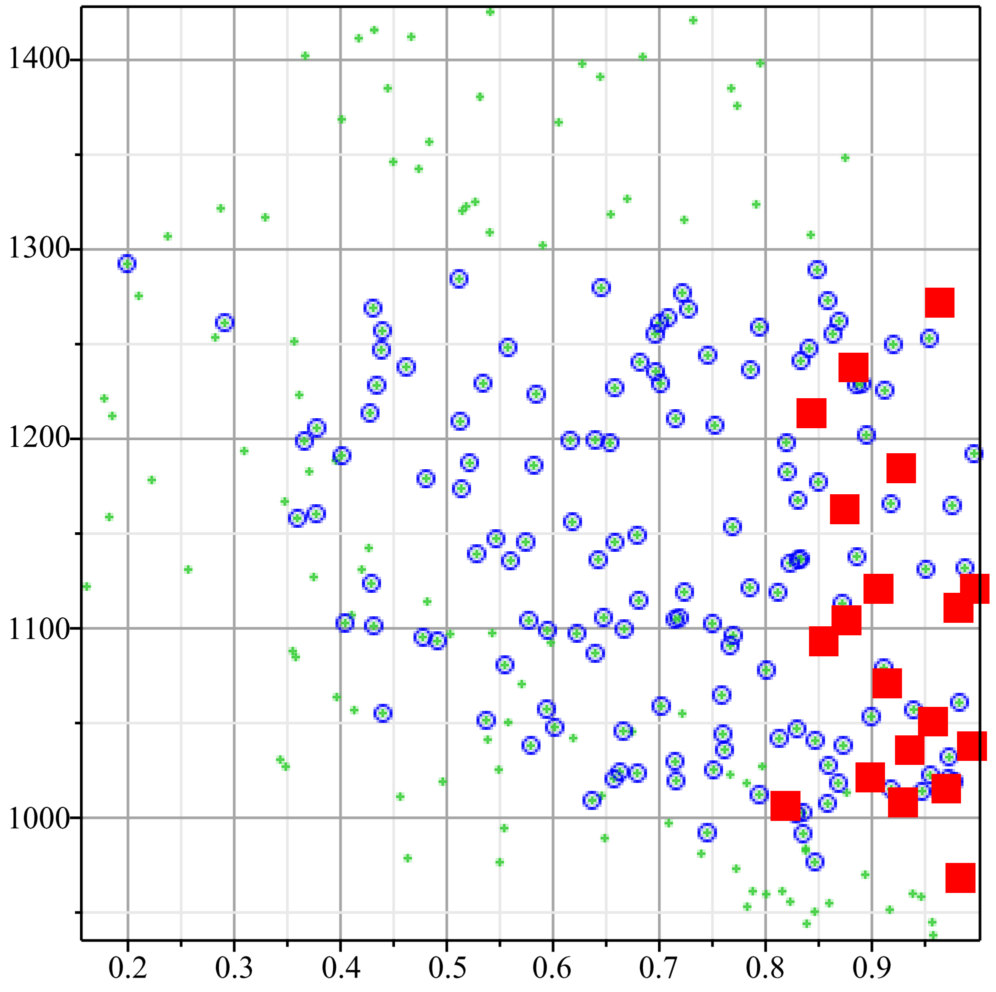


*crvsvarII*(1, 3, *F_1*, *T1*, *Gen_Par_1*, *FEA_1*, *PARETO_CR_1*);

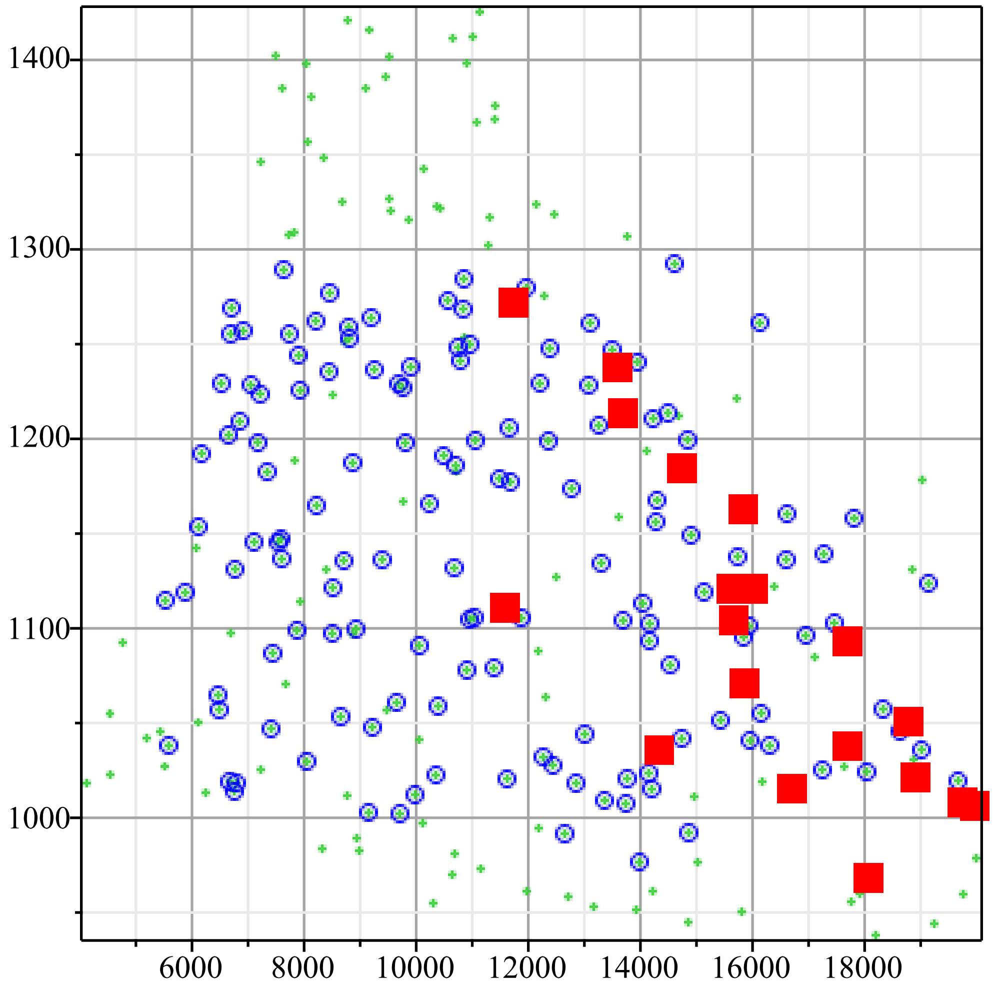


*crvsvarII*(2, 1, *F_1*, *T1*, *Gen_Par_1*, *FEA_1*, *PARETO_CR_1*);

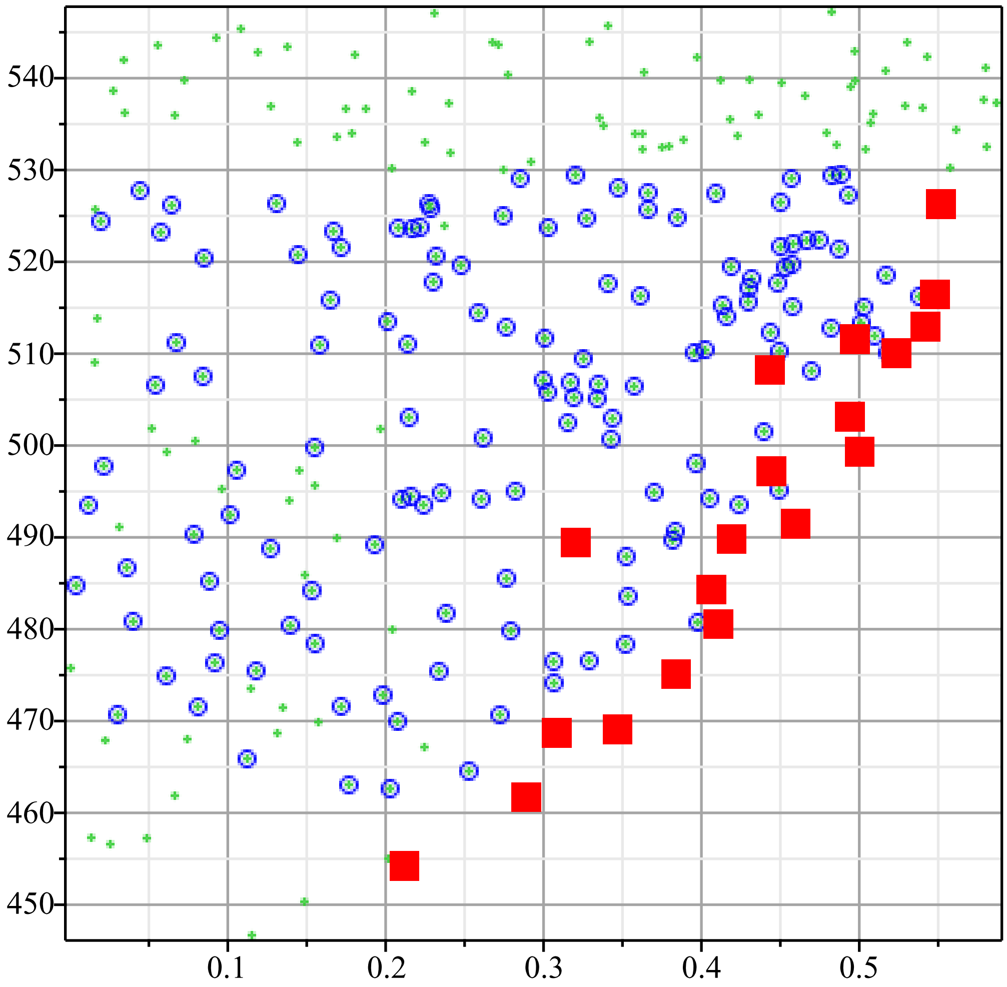


*crvsvarII*(2, 2, *F_1*, *T1*, *Gen_Par_1*, *FEA_1*, *PARETO_CR_1*);

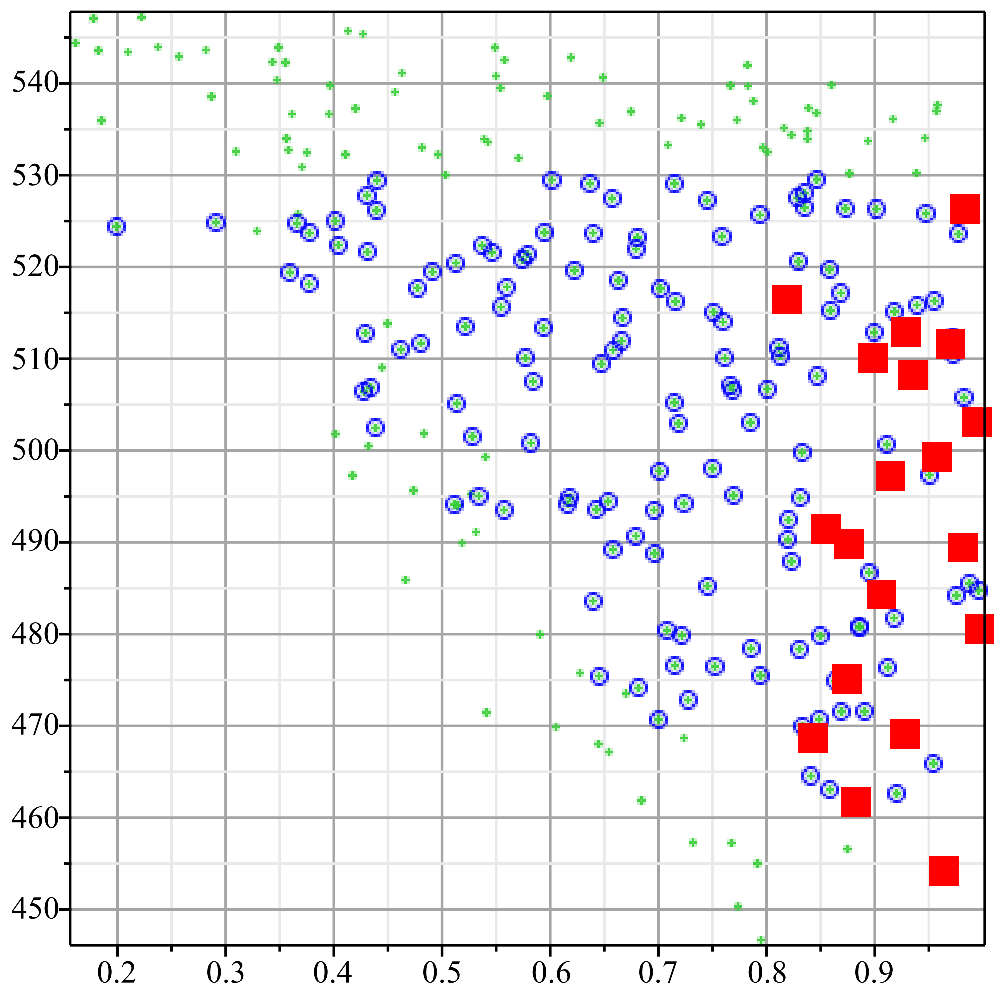


*crvsvarII*(2, 3, *F_1*, *T1*, *Gen_Par_1*, *FEA_1*, *PARETO_CR_1*);

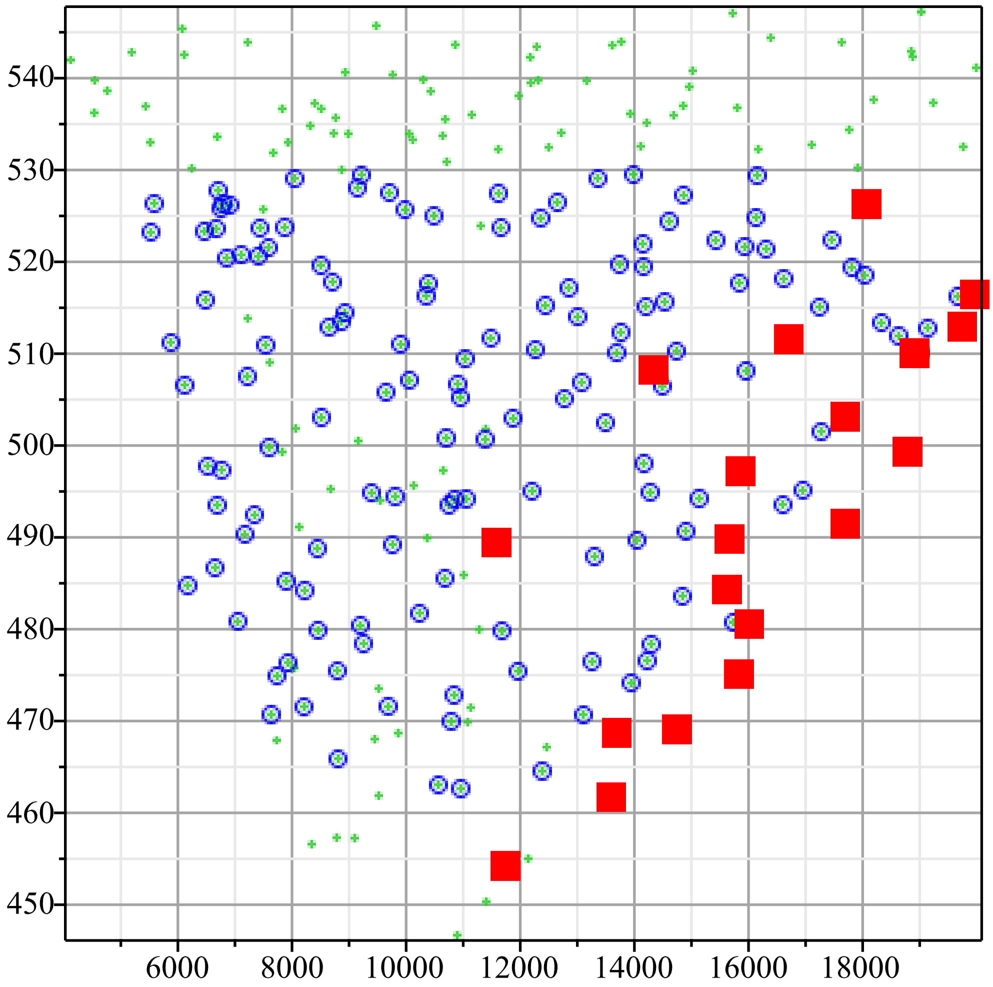


## Graphs "Criterion vs. criterion"

*crvscr*(2, 1, *F_1*, *T1*, *Gen_Par_1*, *FEA_1*, *PARETO_CR_1*);

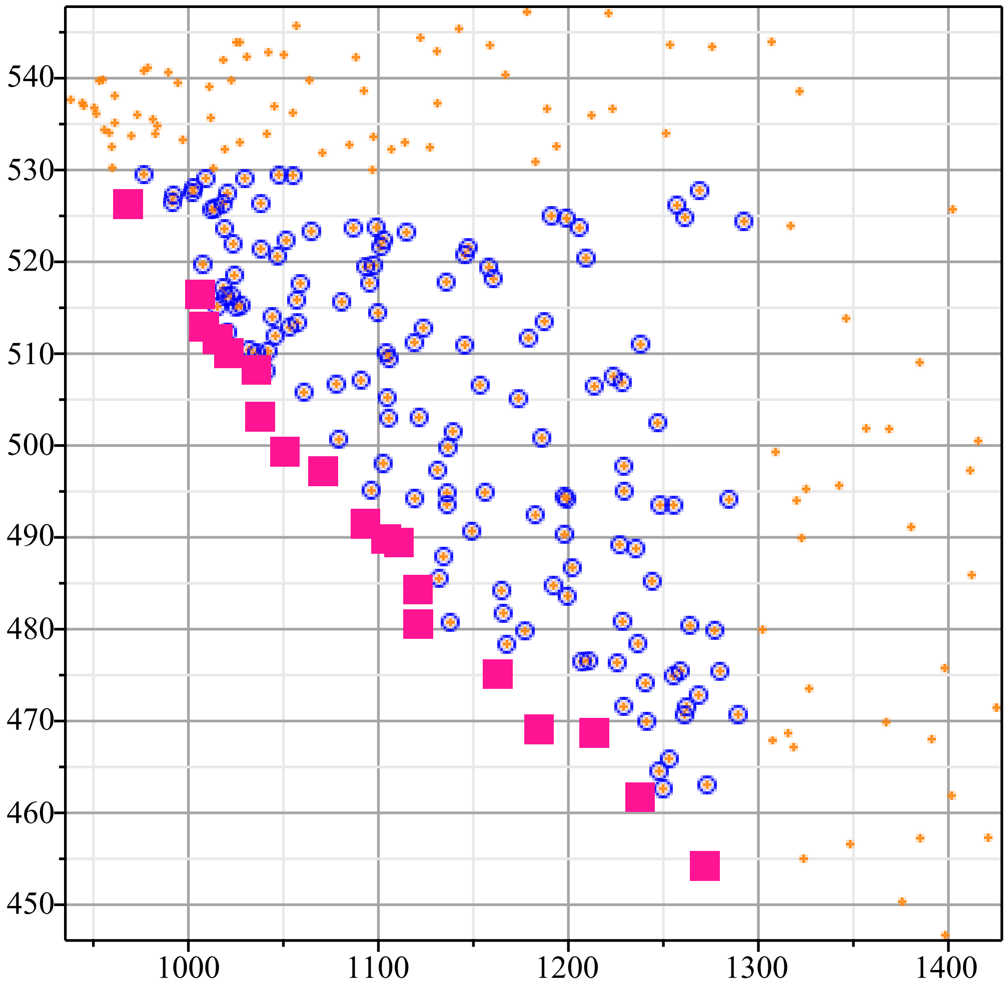


*crvscr*(1, 2, *F_1*, *T1*, *Gen_Par_1*, *FEA_1*, *PARETO_CR_1*);

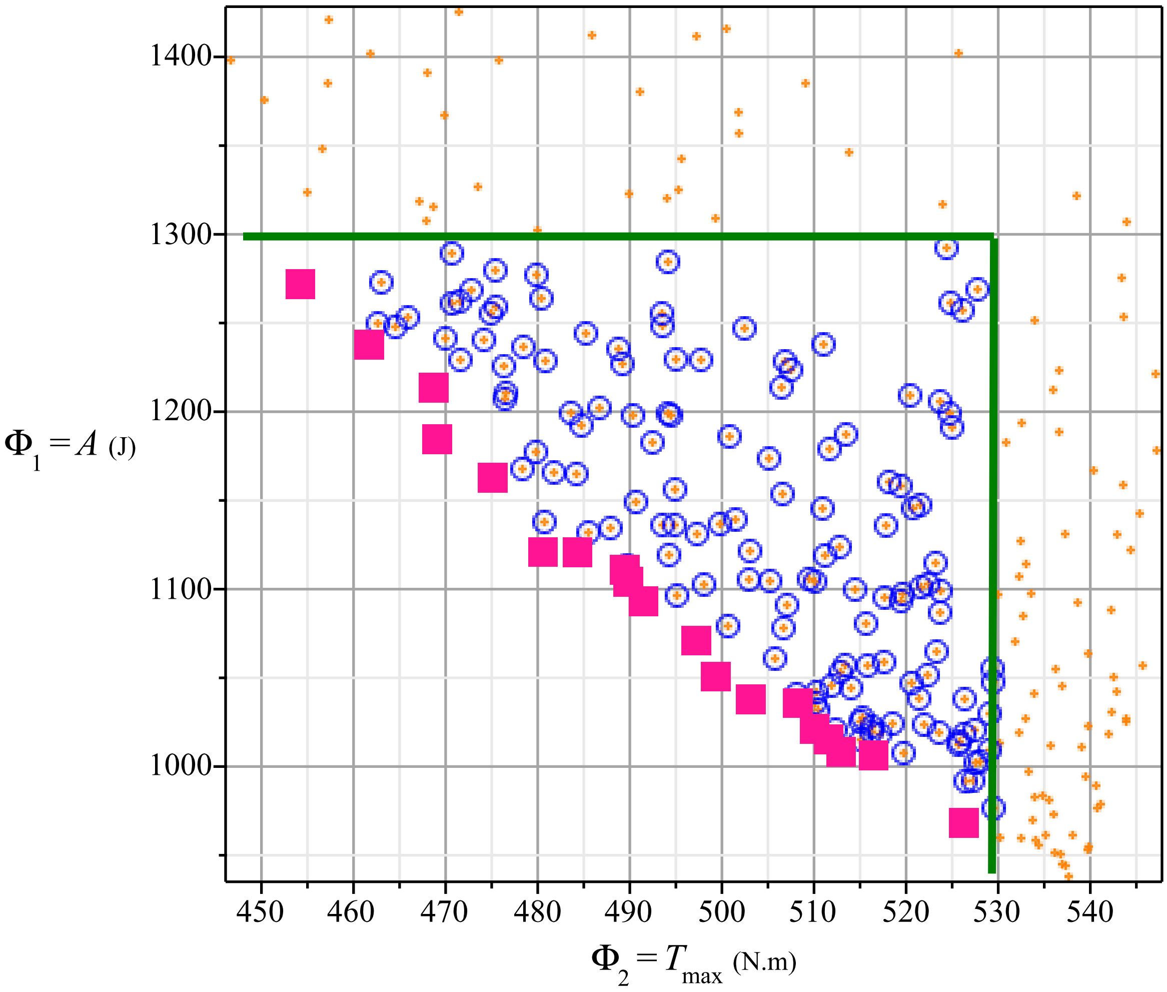


## Histograms of the distribution of feasible solutions

*histogr_vsvar*(1, 15, *Param_1*, *F_1*, *Gen_Par_1*, *T1*, *FEA_1*, *PARETO_CR_1*);

[12, 14, 13, 24, 23, 22, 28, 17, 4, 0, 0, 0, 0, 0, 0]

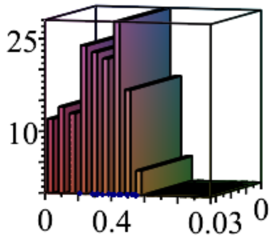


*histogr_vsvar*(2, 15, *Param_1*, *F_1*, *Gen_Par_1*, *T1*, *FEA_1*, *PARETO_CR_1*);

[0, 0, 1, 0, 1, 4, 11, 8, 13, 16, 19, 15, 26, 23, 20]

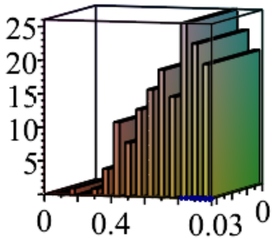


*histogr_vsvar*(3, 15, *Param_1*, *F_1*, *Gen_Par_1*, *T1*, *FEA_1*, *PARETO_CR_1*);

[0, 0, 0, 0, 9, 23, 17, 14, 20, 13, 21, 15, 10, 8, 7]

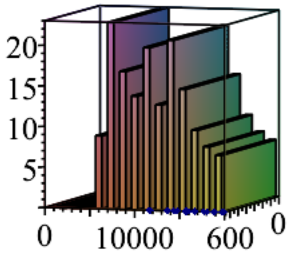


*File2* := *cat*(*currentdir*( ), "\\Excel\\TF1.xlsx");

*File2* := "D:\GGDR\BAO CHI\BAO QUOC TE\Toi uu hoa su dung lo xo TQCT\Excel\TF1.xlsx"

*XuatCach2* := *Export*(*File2*, *F_1*);

*XuatCach2* := ( )

*File3* := *cat*(*currentdir*( ), "\\Excel\\TF2.xlsx");

*File3* := "D:\GGDR\BAO CHI\BAO QUOC TE\Toi uu hoa su dung lo xo TQCT\Excel\TF2.xlsx"

$XuatCach3 := Export(File3, T1);$

$$XuatCach3 := (\ ) \tag{4}$$

$File4 := cat(currentdir(\ ),$ "\\Excel\\TF3.xlsx"$);$

$File4 :=$ "D:\GGDR\BAO CHI\BAO QUOC TE\Toi uu hoa su dung lo xo TQCT\Excel\TF3.xlsx" **(5)**

$XuatCach4 := Export(File4, T1\_sort);$

$$XuatCach4 := (\ ) \tag{6}$$

$File5 := cat(currentdir(\ ),$ "\\Excel\\TFEA_par.xlsx"$);$

$File5 :=$ **(7)**

"D:\GGDR\BAO CHI\BAO QUOC TE\Toi uu hoa su dung lo xo TQCT\Excel\TFEA_par.xlsx"

$XuatCach5 := Export(File5, PARETO\_PAR\_1);$

$$XuatCach5 := (\ ) \tag{8}$$

$File6 := cat(currentdir(\ ),$ "\\Excel\\TFEA_ob.xlsx"$);$

$File6 :=$ **(9)**

"D:\GGDR\BAO CHI\BAO QUOC TE\Toi uu hoa su dung lo xo TQCT\Excel\TFEA_ob.xlsx"

$XuatCach6 := Export(File6, PARETO\_CR\_1);$

$$XuatCach6 := (\ ) \tag{10}$$

$f2(0.3839545949, 0.8744698156, 15833.53815\ )$

$$604.2289671 \tag{11}$$
