# Supplementary material for: Multi-objective optimizing spring placement and stiffness in slider-crank mechanisms for enhanced dynamic parameters
Source: PLoS One. 2025 Sep 8;20(9):e0331341. doi: 10.1371/journal.pone.0331341 (PMC12416651; doi:10.1371/journal.pone.0331341)
Supplement: S1 Data — (ZIP) [file pone.0331341.s001.ZIP › NB(x1,x2)_(For Fig 7).pdf]

$NB\_0 := \mathbf{proc}(x1, x2)$

local $\alpha_1, \alpha_2, k, \varphi, \varepsilon_{OA}, l_1, l_2, \omega_{OA}, m_1, m_2, m_3, g, \mu, F, x_A, y_A, x_B, y_B, x_{G1}, y_{G1}, x_{G2}, y_{G2}, x_O, y_O, x_M, y_M, x_N, y_N, l_0, MN, \Delta l, F_{dh}, \varepsilon_{AB}, v_B, a_B, a_{G1x}, a_{G1y}, a_{G2x}, a_{G2y}, Sys\_dynamic, X_A, Y_A, X_O, Y_O, X_B, Y_B, M, N_B, F_{ms}, n, XX_O, XX_A, XX_B, YY_O, YY_A, YY_B, MM, NN_B, FF_{ms}, i, RR_O, RR_A, RR_B, J_1, J_2, AG_2, AG_1$;

#if not is({args}, set(numeric)) **then return** *('procname')('args')* **end if**;

$\alpha_1 := x1; \alpha_2 := x2; k := 0;$

$l_1 := 0.175; l_2 := 0.58; \omega_{OA} := 3.1416; m_1 := 41.5147; m_2 := 7.656875; m_3 := 9.8996; g := 9.81; \mu := 0.3;\ AG_2 := 0.3165732; AG_1 := 0.0019;$

$J_1 := 0.889678;$

$J_2 := 0.6204482568;$

$x_A := \varphi \to l_1 \cdot \cos(\varphi);$

$y_A := \varphi \to l_1 \cdot \sin(\varphi);$

$x_B := \varphi \to l_1 \cdot \cos(\varphi) + \left(l_2^2 - \left(l_1 \cdot \sin(\varphi)\right)^2\right)^{\frac{1}{2}};$

$y_B := \varphi \to 0;$

$x_{G1} := \varphi \to AG_1 \cdot \cos(\varphi);$

$y_{G1} := \varphi \to AG_1 \cdot \sin(\varphi);$

$x_{G2} := \varphi \to x_A(\varphi) + AG_2 \cdot \sqrt{1 - \frac{l_1^2}{l_2^2} \cdot (\sin(\varphi))^2};$

$y_{G2} := \varphi \to \left(1 - \frac{AG_2}{l_2}\right) \cdot y_A(\varphi);$

$x_O := \varphi \to 0;$

$y_O := \varphi \to 0;$

$v_B := \varphi \to -\left(1 + \frac{l_1 \cdot \cos(\varphi)}{\left(l_2^2 - l_1^2 \cdot (\sin(\varphi))^2\right)^{\frac{1}{2}}}\right) \cdot \omega_{OA} \cdot l_1 \cdot \sin(\varphi);$

$x_M := \varphi \to \alpha_1 \cdot l_1 \cdot \cos(\varphi);$

$y_M := \varphi \to \alpha_1 \cdot l_1 \cdot \sin(\varphi);$

$x_N := \varphi \to l_1 \cdot \cos(\varphi) + \alpha_2 \cdot \sqrt{l_2^2 - l_1^2 \cdot (\sin(\varphi))^2};$

$y_N := \varphi \to (1 - \alpha_2) \cdot l_1 \cdot \sin(\varphi);$

$l_0 := \varphi \to (1 - \alpha_1) \cdot l_1 + \alpha_2 \cdot l_2;$

$MN := \varphi \to 10^{-10} + \sqrt{(x_M(\varphi) - x_N(\varphi))^2 + (y_M(\varphi) - y_N(\varphi))^2};$

$\Delta l := \varphi \to MN(\varphi) - l_0(\varphi);$

$F_{dh} := \varphi \to k \cdot \Delta l(\varphi);$

$\varepsilon_{OA} := \varphi \to 0;$

$$\varepsilon_{AB} := \varphi \to \frac{l_2^{\,2} - l_1^{\,2}}{\left(l_2^{\,2} - l_1^{\,2}\cdot(\sin(\varphi))^2\right)^{\frac{3}{2}}}\cdot\left(\omega_{OA}\right)^2\cdot l_1\cdot\sin(\varphi) - \frac{l_1\cdot\cos(\varphi)}{\left(l_2^{\,2} - l_1^{\,2}\cdot(\sin(\varphi))^2\right)^{\frac{1}{2}}}\cdot\varepsilon_{OA}(\varphi);$$

$$a_B := \varphi \to -\varepsilon_{OA}(\varphi)\cdot l_1\cdot\sin(\varphi)\cdot\left(1 + \frac{l_1\cdot\cos(\varphi)}{\left(l_2^{\,2} - l_1^{\,2}\cdot(\sin(\varphi))^2\right)^{\frac{1}{2}}}\right) + l_1\cdot\omega_{OA}^{\;\;2}$$

$$\cdot\left(\frac{l_2^{\,2} - l_1^{\,2}}{\left(l_2^{\,2} - l_1^{\,2}\cdot(\sin(\varphi))^2\right)^{\frac{3}{2}}}\cdot l_1\cdot(\sin(\varphi))^2 - \cos(\varphi) - \frac{l_1\cdot(\cos(\varphi))^2}{\left(l_2^{\,2} - l_1^{\,2}\cdot(\sin(\varphi))^2\right)^{\frac{1}{2}}}\right);$$

$$a_{G1x} := \varphi \to -AG_1\cdot\left(\varepsilon_{OA}(\varphi)\cdot\sin(\varphi) + \omega_{OA}^{\;\;2}\cdot\cos(\varphi)\right);$$

$$a_{G1y} := \varphi \to AG_1\cdot\left(\varepsilon_{OA}(\varphi)\cdot\cos(\varphi) - \omega_{OA}^{\;\;2}\cdot\sin(\varphi)\right);$$

$$\#a_{G2x} := \varphi \to -l_1\sin(\varphi)\left(1 + \frac{AG_2\, l_1\cos(\varphi)}{\sqrt{1 - \frac{l_1^{\,2}\sin(\varphi)^2}{l_2^{\,2}}}\; l_2^{\,2}}\right)\varepsilon_{OA}(\varphi) - l_1\,\omega_{OA}^{\;\;2}\left(\cos(\varphi)\right.$$

$$\left. + \frac{AG_2\, l_1^{\,3}\sin(\varphi)^2\cos(\varphi)^2}{\left(1 - \frac{l_1^{\,2}\sin(\varphi)^2}{l_2^{\,2}}\right)^{3/2} l_2^{\,4}} + \frac{AG_2\, l_1\cos(2\,\varphi)}{\sqrt{1 - \frac{l_1^{\,2}\sin(\varphi)^2}{l_2^{\,2}}}\; l_2^{\,2}}\right);$$

$$a_{G2x} := \varphi \to -l_1\left(\varepsilon_{OA}(\varphi)\right)\sin(\varphi) - l_1\left(\omega_{OA}\right)^2\cos(\varphi) - \frac{AG_2\, l_1^{\,4}\sin(\varphi)^2\left(\omega_{OA}\right)^2\cos(\varphi)^2}{\left(1 - \frac{l_1^{\,2}\sin(\varphi)^2}{l_2^{\,2}}\right)^{3/2} l_2^{\,4}}$$

$$- \frac{AG_2\, l_1^{\,2}\left(\omega_{OA}\right)^2\cos(\varphi)^2}{\sqrt{1 - \frac{l_1^{\,2}\sin(\varphi)^2}{l_2^{\,2}}}\; l_2^{\,2}} - \frac{AG_2\, l_1^{\,2}\sin(\varphi)\left(\varepsilon_{OA}(\varphi)\right)\cos(\varphi)}{\sqrt{1 - \frac{l_1^{\,2}\sin(\varphi)^2}{l_2^{\,2}}}\; l_2^{\,2}} + \frac{AG_2\, l_1^{\,2}\sin(\varphi)^2\left(\omega_{OA}\right)^2}{\sqrt{1 - \frac{l_1^{\,2}\sin(\varphi)^2}{l_2^{\,2}}}\; l_2^{\,2}};$$

$$\#a_{G2y} := \varphi \to \frac{\left(-l_2 + AG_2\right) l_1\left(\omega_{OA}^{\;\;2}\sin(\varphi) - \varepsilon_{OA}(\varphi)\cos(\varphi)\right)}{l_2};$$

$$a_{G2y} := \varphi \to \left(1 - \frac{AG_2}{l_2}\right) l_1\left(\varepsilon_{OA}(\varphi)\right)\cos(\varphi) - \left(1 - \frac{AG_2}{l_2}\right) l_1\left(\omega_{OA}\right)^2\sin(\varphi);$$

$n := 360;$

$XX_{\mathrm{O}} := Matrix(n, 2) :$

$XX_A := Matrix(n, 2) :$

$YY_{\mathrm{O}} := Matrix(n, 2) :$

$YY_A := Matrix(n, 2) :$

$MM := Matrix(n, 2) :$

$XX_B := Matrix(n, 2) :$

$YY_B := Matrix(n, 2) :$

$NN_B := Matrix(n, 2) :$

$FF_{ms} := Matrix(n, 2):$
$RR_{O} := Matrix(n, 2):$
$RR_{A} := Matrix(n, 2):$
$RR_{B} := Matrix(n, 2):$
$F := Matrix(n, 1):$
for *i* **from** 271 **to** 360 **do**
$F[i] := -3233;$
od:
for *i* **from** 1 **by** 1 **to** *n* **do**

$$\varphi := \frac{(i-1)\cdot\pi}{180};$$

$$Sys_dynamic := \left\{ \begin{aligned}
& X_O + X_A + F_{dh}(\varphi)\cdot\frac{x_N(\varphi) - x_M(\varphi)}{MN(\varphi)} = m_1\cdot a_{G1x}(\varphi), \\
& Y_O + Y_A - m_1\cdot g + F_{dh}(\varphi)\cdot\frac{y_N(\varphi) - y_M(\varphi)}{MN(\varphi)} = m_1\cdot a_{G1y}(\varphi), \\
& M + \left(x_A(\varphi) - x_{G1}(\varphi)\right)\cdot Y_A - \left(y_A(\varphi) - y_{G1}(\varphi)\right)\cdot X_A + \left(x_O(\varphi) - x_{G1}(\varphi)\right)\cdot Y_O - \left(y_O(\varphi) - y_{G1}(\varphi)\right) \\
& \quad \cdot X_O + \left(x_M(\varphi) - x_{G1}(\varphi)\right)\cdot F_{dh}(\varphi)\cdot\frac{y_N(\varphi) - y_M(\varphi)}{MN(\varphi)} - \left(y_M(\varphi) - y_{G1}(\varphi)\right)\cdot F_{dh}(\varphi) \\
& \quad \cdot\frac{x_N(\varphi) - x_M(\varphi)}{MN(\varphi)} = J_1\cdot\varepsilon_{OA}(\varphi), \\
& -X_A + X_B + F_{dh}(\varphi)\cdot\frac{x_M(\varphi) - x_N(\varphi)}{MN(\varphi)} = m_2\cdot a_{G2x}(\varphi), \\
& -Y_A + Y_B - m_2\cdot g + F_{dh}(\varphi)\cdot\frac{y_M(\varphi) - y_N(\varphi)}{MN(\varphi)} = m_2\cdot a_{G2y}(\varphi), \\
& \left(x_A(\varphi) - x_{G2}(\varphi)\right)\cdot\left(-Y_A\right) - \left(y_A(\varphi) - y_{G2}(\varphi)\right)\cdot\left(-X_A\right) + \left(x_B(\varphi) - x_{G2}(\varphi)\right)\cdot Y_B - \left(y_B(\varphi)\right. \\
& \quad \left. - y_{G2}(\varphi)\right)\cdot X_B + \left(x_N(\varphi) - x_{G2}(\varphi)\right)\cdot F_{dh}(\varphi)\cdot\frac{y_M(\varphi) - y_N(\varphi)}{MN(\varphi)} - \left(y_N(\varphi) - y_{G2}(\varphi)\right)\cdot F_{dh}(\varphi) \\
& \quad \cdot\frac{x_M(\varphi) - x_N(\varphi)}{MN(\varphi)} = J_2\cdot\varepsilon_{AB}(\varphi), \\
& Y_B + m_3\cdot g = N_B, \\
& -\mu\cdot\left|N_B\right|\cdot\frac{v_B(\varphi)}{\left|v_B(\varphi)\right| + 10^{-6}} = F_{ms}, \\
& -X_B + F_{ms} + F(i) = m_3\cdot a_B(\varphi)
\end{aligned} \right\}:$$

$fsolve\left(Sys\_dynamic, \left\{X_A, Y_A, X_O, Y_O, X_B, Y_B, M, N_B, F_{ms}\right\}\right): assign(\%);$

```
XX_O(i, 1) := i; XX_A(i, 1) := i; XX_B(i, 1) = i; YY_O(i, 1) := i; YY_A(i, 1) := i; YY_B(i, 1) = i; MM(i, 1) :=
    i; NN_B(i, 1) := i; FF_ms(i, 1) := i; RR_O(i, 1) := i; RR_A(i, 1) := i; RR_B(i, 1) := i;
XX_O(i, 2) := X_O; XX_A(i, 2) := X_A; XX_B(i, 2) = X_B; YY_O(i, 2) := Y_O; YY_A(i, 2) := Y_A; YY_B(i, 2) = Y_B;
    MM(i, 2) := M; NN_B(i, 2) := N_B; FF_ms(i, 2) := F_ms;
RR_O(i, 2) := sqrt((X_O)^2 + (Y_O)^2);
RR_A(i, 2) := sqrt((X_A)^2 + (Y_A)^2);
RR_B(i, 2) := sqrt((X_B)^2 + (Y_B)^2);
unassign('X_O','X_A','X_B','Y_O','Y_A','Y_B','M','N_B','F_ms');
od:
return max(|NN_B|);
end proc:
NB_1000 := proc(x1, x2)
local α_1, α_2, k, φ, ε_OA, l_1, l_2, ω_OA, m_1, m_2, m_3, g, μ, F, x_A, y_A, x_B, y_B, x_G1, y_G1, x_G2, y_G2, x_O, y_O, x_M, y_M, x_N, y_N,
    l_0, MN, Δl, F_dh, ε_AB, v_B, a_B, a_G1x, a_G1y, a_G2x, a_G2y, Sys_dynamic, X_A, Y_A, X_O, Y_O, X_B, Y_B, M, N_B, F_ms, n,
    XX_O, XX_A, XX_B, YY_O, YY_A, YY_B, MM, NN_B, FF_ms, i, RR_O, RR_A, RR_B, J_1, J_2, AG_2, AG_1;
#if not is({args}, set(numeric)) then return ('procname')('args') end if;
α_1 := x1; α_2 := x2; k := 1000;
l_1 := 0.175; l_2 := 0.58; ω_OA := 3.1416; m_1 := 41.5147; m_2 := 7.656875; m_3 := 9.8996; g := 9.81; μ :=
    0.3; AG_2 := 0.3165732; AG_1 := 0.0019;
J_1 := 0.889678;
J_2 := 0.6204482568;
```

$x_A := \varphi \to l_1 \cdot \cos(\varphi);$

$y_A := \varphi \to l_1 \cdot \sin(\varphi);$

$x_B := \varphi \to l_1 \cdot \cos(\varphi) + \left(l_2^2 - (l_1 \cdot \sin(\varphi))^2\right)^{\frac{1}{2}};$

$y_B := \varphi \to 0;$

$x_{G1} := \varphi \to AG_1 \cdot \cos(\varphi);$

$y_{G1} := \varphi \to AG_1 \cdot \sin(\varphi);$

$x_{G2} := \varphi \to x_A(\varphi) + AG_2 \cdot \sqrt{1 - \frac{l_1^2}{l_2^2} \cdot (\sin(\varphi))^2};$

$y_{G2} := \varphi \to \left(1 - \frac{AG_2}{l_2}\right) \cdot y_A(\varphi);$

$x_O := \varphi \to 0;$

$y_O := \varphi \to 0;$

$v_B := \varphi \to -\left(1 + \frac{l_1 \cdot \cos(\varphi)}{\left(l_2^2 - l_1^2 \cdot (\sin(\varphi))^2\right)^{\frac{1}{2}}}\right) \cdot \omega_{OA} \cdot l_1 \cdot \sin(\varphi);$

$$x_M := \varphi \to \alpha_1 \cdot l_1 \cdot \cos(\varphi);$$
$$y_M := \varphi \to \alpha_1 \cdot l_1 \cdot \sin(\varphi);$$
$$x_N := \varphi \to l_1 \cdot \cos(\varphi) + \alpha_2 \cdot \sqrt{l_2^2 - l_1^2 \cdot (\sin(\varphi))^2};$$
$$y_N := \varphi \to \left(1 - \alpha_2\right) \cdot l_1 \cdot \sin(\varphi);$$
$$l_0 := \varphi \to \left(1 - \alpha_1\right) \cdot l_1 + \alpha_2 \cdot l_2;$$
$$MN := \varphi \to 10^{-10} + \sqrt{\left(x_M(\varphi) - x_N(\varphi)\right)^2 + \left(y_M(\varphi) - y_N(\varphi)\right)^2};$$
$$\Delta l := \varphi \to MN(\varphi) - l_0(\varphi);$$
$$F_{dh} := \varphi \to k \cdot \Delta l(\varphi);$$
$$\varepsilon_{OA} := \varphi \to 0;$$
$$\varepsilon_{AB} := \varphi \to \frac{l_2^2 - l_1^2}{\left(l_2^2 - l_1^2 \cdot (\sin(\varphi))^2\right)^{\frac{3}{2}}} \cdot \left(\omega_{OA}\right)^2 \cdot l_1 \cdot \sin(\varphi) - \frac{l_1 \cdot \cos(\varphi)}{\left(l_2^2 - l_1^2 \cdot (\sin(\varphi))^2\right)^{\frac{1}{2}}} \cdot \varepsilon_{OA}(\varphi);$$
$$a_B := \varphi \to -\varepsilon_{OA}(\varphi) \cdot l_1 \cdot \sin(\varphi) \cdot \left(1 + \frac{l_1 \cdot \cos(\varphi)}{\left(l_2^2 - l_1^2 \cdot (\sin(\varphi))^2\right)^{\frac{1}{2}}}\right) + l_1 \cdot \omega_{OA}^2 \cdot \left(\frac{l_2^2 - l_1^2}{\left(l_2^2 - l_1^2 \cdot (\sin(\varphi))^2\right)^{\frac{3}{2}}} \cdot l_1 \cdot (\sin(\varphi))^2 - \cos(\varphi) - \frac{l_1 \cdot (\cos(\varphi))^2}{\left(l_2^2 - l_1^2 \cdot (\sin(\varphi))^2\right)^{\frac{1}{2}}}\right);$$
$$a_{G1x} := \varphi \to -AG_1 \cdot \left(\varepsilon_{OA}(\varphi) \cdot \sin(\varphi) + \omega_{OA}^2 \cdot \cos(\varphi)\right);$$
$$a_{G1y} := \varphi \to AG_1 \cdot \left(\varepsilon_{OA}(\varphi) \cdot \cos(\varphi) - \omega_{OA}^2 \cdot \sin(\varphi)\right);$$
$$\#a_{G2x} := \varphi \to -l_1 \sin(\varphi) \left(1 + \frac{AG_2\, l_1 \cos(\varphi)}{\sqrt{1 - \frac{l_1^2 \sin(\varphi)^2}{l_2^2}}\; l_2}\right) \varepsilon_{OA}(\varphi) - l_1\, \omega_{OA}^2 \left(\cos(\varphi) + \frac{AG_2\, l_1^3 \sin(\varphi)^2 \cos(\varphi)^2}{\left(1 - \frac{l_1^2 \sin(\varphi)^2}{l_2^2}\right)^{3/2} l_2^4} + \frac{AG_2\, l_1 \cos(2\,\varphi)}{\sqrt{1 - \frac{l_1^2 \sin(\varphi)^2}{l_2^2}}\; l_2^2}\right);$$
$$a_{G2x} := \varphi \to -l_1 \left(\varepsilon_{OA}(\varphi)\right) \sin(\varphi) - l_1 \left(\omega_{OA}\right)^2 \cos(\varphi) - \frac{AG_2\, l_1^4 \sin(\varphi)^2 \left(\omega_{OA}\right)^2 \cos(\varphi)^2}{\left(1 - \frac{l_1^2 \sin(\varphi)^2}{l_2^2}\right)^{3/2} l_2^4} - \frac{AG_2\, l_1^2 \left(\omega_{OA}\right)^2 \cos(\varphi)^2}{\sqrt{1 - \frac{l_1^2 \sin(\varphi)^2}{l_2^2}}\; l_2^2} - \frac{AG_2\, l_1^2 \sin(\varphi) \left(\varepsilon_{OA}(\varphi)\right) \cos(\varphi)}{\sqrt{1 - \frac{l_1^2 \sin(\varphi)^2}{l_2^2}}\; l_2^2} + \frac{AG_2\, l_1^2 \sin(\varphi)^2 \left(\omega_{OA}\right)^2}{\sqrt{1 - \frac{l_1^2 \sin(\varphi)^2}{l_2^2}}\; l_2^2};$$
$$\#a_{G2y} := \varphi \to \frac{\left(-l_2 + AG_2\right) l_1 \left(\omega_{OA}^2 \sin(\varphi) - \varepsilon_{OA}(\varphi) \cos(\varphi)\right)}{l_2};$$

$a_{G2y} := \varphi \rightarrow \left(1 - \frac{AG_2}{l_2}\right) l_1 \left(\varepsilon_{OA}(\varphi)\right) \cos(\varphi) - \left(1 - \frac{AG_2}{l_2}\right) l_1 \left(\omega_{OA}\right)^2 \sin(\varphi);$

$n := 360;$

$XX_O := Matrix(n, 2) :$

$XX_A := Matrix(n, 2) :$

$YY_O := Matrix(n, 2) :$

$YY_A := Matrix(n, 2) :$

$MM := Matrix(n, 2) :$

$XX_B := Matrix(n, 2) :$

$YY_B := Matrix(n, 2) :$

$NN_B := Matrix(n, 2) :$

$FF_{ms} := Matrix(n, 2) :$

$RR_O := Matrix(n, 2) :$

$RR_A := Matrix(n, 2) :$

$RR_B := Matrix(n, 2) :$

$F := Matrix(n, 1) :$

for *i* **from** 271 **to** 360 **do**

$F[i] := -3233;$

od:

for *i* **from** 1 **by** 1 **to** *n* **do**

$\varphi := \frac{(i-1)\cdot\pi}{180};$

$Sys\_dynamic := \Bigg\{$

$X_O + X_A + F_{dh}(\varphi)\cdot\frac{x_N(\varphi) - x_M(\varphi)}{MN(\varphi)} = m_1\cdot a_{G1x}(\varphi),$

$Y_O + Y_A - m_1\cdot g + F_{dh}(\varphi)\cdot\frac{y_N(\varphi) - y_M(\varphi)}{MN(\varphi)} = m_1\cdot a_{G1y}(\varphi),$

$M + \left(x_A(\varphi) - x_{G1}(\varphi)\right)\cdot Y_A - \left(y_A(\varphi) - y_{G1}(\varphi)\right)\cdot X_A + \left(x_O(\varphi) - x_{G1}(\varphi)\right)\cdot Y_O - \left(y_O(\varphi) - y_{G1}(\varphi)\right)$
$\cdot X_O + \left(x_M(\varphi) - x_{G1}(\varphi)\right)\cdot F_{dh}(\varphi)\cdot\frac{y_N(\varphi) - y_M(\varphi)}{MN(\varphi)} - \left(y_M(\varphi) - y_{G1}(\varphi)\right)\cdot F_{dh}(\varphi)$
$\cdot\frac{x_N(\varphi) - x_M(\varphi)}{MN(\varphi)} = J_1\cdot\varepsilon_{OA}(\varphi),$

$-X_A + X_B + F_{dh}(\varphi)\cdot\frac{x_M(\varphi) - x_N(\varphi)}{MN(\varphi)} = m_2\cdot a_{G2x}(\varphi),$

$-Y_A + Y_B - m_2\cdot g + F_{dh}(\varphi)\cdot\frac{y_M(\varphi) - y_N(\varphi)}{MN(\varphi)} = m_2\cdot a_{G2y}(\varphi),$

$\left(x_A(\varphi) - x_{G2}(\varphi)\right)\cdot\left(-Y_A\right) - \left(y_A(\varphi) - y_{G2}(\varphi)\right)\cdot\left(-X_A\right) + \left(x_B(\varphi) - x_{G2}(\varphi)\right)\cdot Y_B - \left(y_B(\varphi)\right.$

$$- y_{G2}(\varphi)\big)\cdot X_B + \big(x_N(\varphi) - x_{G2}(\varphi)\big)\cdot F_{dh}(\varphi)\cdot \frac{y_M(\varphi) - y_N(\varphi)}{MN(\varphi)} - \big(y_N(\varphi) - y_{G2}(\varphi)\big)\cdot F_{dh}(\varphi) \cdot \frac{x_M(\varphi) - x_N(\varphi)}{MN(\varphi)} = J_2\cdot \varepsilon_{AB}(\varphi),$$

$$Y_B + m_3\cdot g = N_B,$$

$$-\mu\cdot |N_B|\cdot \frac{v_B(\varphi)}{|v_B(\varphi)| + 10^{-6}} = F_{ms},$$

$$-X_B + F_{ms} + F(i) = m_3\cdot a_B(\varphi) \Big\}:$$

$fsolve\big(Sys\_dynamic, \{X_A, Y_A, X_O, Y_O, X_B, Y_B, M, N_B, F_{ms}\}\big) : assign(\%);$

$XX_O(i,1) := i; XX_A(i,1) := i; XX_B(i,1) = i; YY_O(i,1) := i; YY_A(i,1) := i; YY_B(i,1) = i; MM(i,1) := i; NN_B(i,1) := i; FF_{ms}(i,1) := i; RR_O(i,1) := i; RR_A(i,1) := i; RR_B(i,1) := i;$

$XX_O(i,2) := X_O; XX_A(i,2) := X_A; XX_B(i,2) = X_B; YY_O(i,2) := Y_O; YY_A(i,2) := Y_A; YY_B(i,2) = Y_B; MM(i,2) := M; NN_B(i,2) := N_B; FF_{ms}(i,2) := F_{ms};$

$RR_O(i,2) := \sqrt{(X_O)^2 + (Y_O)^2};$

$RR_A(i,2) := \sqrt{(X_A)^2 + (Y_A)^2};$

$RR_B(i,2) := \sqrt{(X_B)^2 + (Y_B)^2};$

$unassign('X_O','X_A','X_B','Y_O','Y_A','Y_B','M','N_B','F_{ms}');$

od:

return $\max(|NN_B|);$

end proc:

$NB\_5000 :=$ **proc**$(x1, x2)$

local $\alpha_1, \alpha_2, k, \varphi, \varepsilon_{OA}, l_1, l_2, \omega_{OA}, m_1, m_2, m_3, g, \mu, F, x_A, y_A, x_B, y_B, x_{G1}, y_{G1}, x_{G2}, y_{G2}, x_O, y_O, x_M, y_M, x_N, y_N, l_0, MN, \Delta l, F_{dh}, \varepsilon_{AB}, v_B, a_B, a_{G1x}, a_{G1y}, a_{G2x}, a_{G2y}, Sys\_dynamic, X_A, Y_A, X_O, Y_O, X_B, Y_B, M, N_B, F_{ms}, n, XX_O, XX_A, XX_B, YY_O, YY_A, YY_B, MM, NN_B, FF_{ms}, i, RR_O, RR_A, RR_B, J_1, J_2, AG_2, AG_1;$

#if not is({args}, set(numeric)) **then return** *('procname')('args')* **end if**;

$\alpha_1 := x1; \alpha_2 := x2; k := 5000;$

$l_1 := 0.175; l_2 := 0.58; \omega_{OA} := 3.1416; m_1 := 41.5147; m_2 := 7.656875; m_3 := 9.8996; g := 9.81; \mu := 0.3;\ AG_2 := 0.3165732; AG_1 := 0.0019;$

$J_1 := 0.889678;$

$J_2 := 0.6204482568;$

$x_A := \varphi \to l_1\cdot\cos(\varphi);$

$y_A := \varphi \to l_1\cdot\sin(\varphi);$

$x_B := \varphi \to l_1\cdot\cos(\varphi) + \big(l_2^2 - (l_1\cdot\sin(\varphi))^2\big)^{\frac{1}{2}};$

$y_B := \varphi \to 0;$

$$x_{G1} := \varphi \rightarrow AG_1 \cdot \cos(\varphi);$$
$$y_{G1} := \varphi \rightarrow AG_1 \cdot \sin(\varphi);$$
$$x_{G2} := \varphi \rightarrow x_A(\varphi) + AG_2 \cdot \sqrt{1 - \frac{l_1^{\,2}}{l_2^{\,2}} \cdot (\sin(\varphi))^2};$$
$$y_{G2} := \varphi \rightarrow \left(1 - \frac{AG_2}{l_2}\right) \cdot y_A(\varphi);$$
$$x_{\mathrm{O}} := \varphi \rightarrow 0;$$
$$y_{\mathrm{O}} := \varphi \rightarrow 0;$$
$$v_B := \varphi \rightarrow -\left(1 + \frac{l_1 \cdot \cos(\varphi)}{\left(l_2^{\,2} - l_1^{\,2} \cdot (\sin(\varphi))^2\right)^{\frac{1}{2}}}\right) \cdot \omega_{OA} \cdot l_1 \cdot \sin(\varphi);$$
$$x_M := \varphi \rightarrow \alpha_1 \cdot l_1 \cdot \cos(\varphi);$$
$$y_M := \varphi \rightarrow \alpha_1 \cdot l_1 \cdot \sin(\varphi);$$
$$x_N := \varphi \rightarrow l_1 \cdot \cos(\varphi) + \alpha_2 \cdot \sqrt{l_2^{\,2} - l_1^{\,2} \cdot (\sin(\varphi))^2};$$
$$y_N := \varphi \rightarrow (1 - \alpha_2) \cdot l_1 \cdot \sin(\varphi);$$
$$l_0 := \varphi \rightarrow (1 - \alpha_1) \cdot l_1 + \alpha_2 \cdot l_2;$$
$$MN := \varphi \rightarrow 10^{-10} + \sqrt{(x_M(\varphi) - x_N(\varphi))^2 + (y_M(\varphi) - y_N(\varphi))^2};$$
$$\Delta l := \varphi \rightarrow MN(\varphi) - l_0(\varphi);$$
$$F_{dh} := \varphi \rightarrow k \cdot \Delta l(\varphi);$$
$$\varepsilon_{OA} := \varphi \rightarrow 0;$$
$$\varepsilon_{AB} := \varphi \rightarrow \frac{l_2^{\,2} - l_1^{\,2}}{\left(l_2^{\,2} - l_1^{\,2} \cdot (\sin(\varphi))^2\right)^{\frac{3}{2}}} \cdot (\omega_{OA})^2 \cdot l_1 \cdot \sin(\varphi) - \frac{l_1 \cdot \cos(\varphi)}{\left(l_2^{\,2} - l_1^{\,2} \cdot (\sin(\varphi))^2\right)^{\frac{1}{2}}} \cdot \varepsilon_{OA}(\varphi);$$
$$a_B := \varphi \rightarrow -\varepsilon_{OA}(\varphi) \cdot l_1 \cdot \sin(\varphi) \cdot \left(1 + \frac{l_1 \cdot \cos(\varphi)}{\left(l_2^{\,2} - l_1^{\,2} \cdot (\sin(\varphi))^2\right)^{\frac{1}{2}}}\right) + l_1 \cdot \omega_{OA}^{\;2} \cdot \left(\frac{l_2^{\,2} - l_1^{\,2}}{\left(l_2^{\,2} - l_1^{\,2} \cdot (\sin(\varphi))^2\right)^{\frac{3}{2}}} \cdot l_1 \cdot (\sin(\varphi))^2 - \cos(\varphi) - \frac{l_1 \cdot (\cos(\varphi))^2}{\left(l_2^{\,2} - l_1^{\,2} \cdot (\sin(\varphi))^2\right)^{\frac{1}{2}}}\right);$$
$$a_{G1x} := \varphi \rightarrow -AG_1 \cdot \left(\varepsilon_{OA}(\varphi) \cdot \sin(\varphi) + \omega_{OA}^{\;2} \cdot \cos(\varphi)\right);$$
$$a_{G1y} := \varphi \rightarrow AG_1 \cdot \left(\varepsilon_{OA}(\varphi) \cdot \cos(\varphi) - \omega_{OA}^{\;2} \cdot \sin(\varphi)\right);$$
$$\#a_{G2x} := \varphi \rightarrow -l_1 \sin(\varphi) \left(1 + \frac{AG_2\, l_1 \cos(\varphi)}{\sqrt{1 - \frac{l_1^{\,2} \sin(\varphi)^2}{l_2^{\,2}}}\; l_2^{\,2}}\right) \varepsilon_{OA}(\varphi) - l_1\, \omega_{OA}^{\;2} \left(\cos(\varphi)\right.$$

$$+\frac{AG_2\, l_1^3 \sin(\varphi)^2 \cos(\varphi)^2}{\left(1-\frac{l_1^2 \sin(\varphi)^2}{l_2^2}\right)^{3/2} l_2^4} + \frac{AG_2\, l_1 \cos(2\,\varphi)}{\sqrt{1-\frac{l_1^2 \sin(\varphi)^2}{l_2^2}}\; l_2^2}\Bigg);$$

$$a_{G2x} := \varphi \rightarrow -l_1\left(\varepsilon_{OA}(\varphi)\right)\sin(\varphi) - l_1\left(\omega_{OA}\right)^2\cos(\varphi) - \frac{AG_2\, l_1^4 \sin(\varphi)^2 \left(\omega_{OA}\right)^2 \cos(\varphi)^2}{\left(1-\frac{l_1^2 \sin(\varphi)^2}{l_2^2}\right)^{3/2} l_2^4}$$

$$-\frac{AG_2\, l_1^2 \left(\omega_{OA}\right)^2 \cos(\varphi)^2}{\sqrt{1-\frac{l_1^2 \sin(\varphi)^2}{l_2^2}}\; l_2^2} - \frac{AG_2\, l_1^2 \sin(\varphi)\left(\varepsilon_{OA}(\varphi)\right)\cos(\varphi)}{\sqrt{1-\frac{l_1^2 \sin(\varphi)^2}{l_2^2}}\; l_2^2} + \frac{AG_2\, l_1^2 \sin(\varphi)^2 \left(\omega_{OA}\right)^2}{\sqrt{1-\frac{l_1^2 \sin(\varphi)^2}{l_2^2}}\; l_2^2};$$

$$\#a_{G2y} := \varphi \rightarrow \frac{\left(-l_2 + AG_2\right) l_1 \left(\omega_{OA}^2 \sin(\varphi) - \varepsilon_{OA}(\varphi)\cos(\varphi)\right)}{l_2};$$

$$a_{G2y} := \varphi \rightarrow \left(1-\frac{AG_2}{l_2}\right) l_1\left(\varepsilon_{OA}(\varphi)\right)\cos(\varphi) - \left(1-\frac{AG_2}{l_2}\right) l_1\left(\omega_{OA}\right)^2\sin(\varphi);$$

$n := 360;$
$XX_{\mathrm{O}} := Matrix(n, 2):$
$XX_A := Matrix(n, 2):$
$YY_{\mathrm{O}} := Matrix(n, 2):$
$YY_A := Matrix(n, 2):$
$MM := Matrix(n, 2):$
$XX_B := Matrix(n, 2):$
$YY_B := Matrix(n, 2):$
$NN_B := Matrix(n, 2):$
$FF_{ms} := Matrix(n, 2):$
$RR_{\mathrm{O}} := Matrix(n, 2):$
$RR_A := Matrix(n, 2):$
$RR_B := Matrix(n, 2):$
$F := Matrix(n, 1):$
for *i* **from** 271 **to** 360 **do**
$F[i] := -3233;$
od:
for *i* **from** 1 **by** 1 **to** *n* **do**
$\varphi := \frac{(i-1)\cdot\pi}{180};$

$Sys\_dynamic := \Bigg\{$

$$X_{O} + X_A + F_{dh}(\varphi)\cdot\frac{x_N(\varphi) - x_M(\varphi)}{MN(\varphi)} = m_1\cdot a_{G1x}(\varphi),$$

$$Y_{O} + Y_A - m_1\cdot g + F_{dh}(\varphi)\cdot\frac{y_N(\varphi) - y_M(\varphi)}{MN(\varphi)} = m_1\cdot a_{G1y}(\varphi),$$

$$M + \left(x_A(\varphi) - x_{G1}(\varphi)\right)\cdot Y_A - \left(y_A(\varphi) - y_{G1}(\varphi)\right)\cdot X_A + \left(x_{O}(\varphi) - x_{G1}(\varphi)\right)\cdot Y_{O} - \left(y_{O}(\varphi) - y_{G1}(\varphi)\right)\cdot X_{O} + \left(x_M(\varphi) - x_{G1}(\varphi)\right)\cdot F_{dh}(\varphi)\cdot\frac{y_N(\varphi) - y_M(\varphi)}{MN(\varphi)} - \left(y_M(\varphi) - y_{G1}(\varphi)\right)\cdot F_{dh}(\varphi)\cdot\frac{x_N(\varphi) - x_M(\varphi)}{MN(\varphi)} = J_1\cdot\varepsilon_{OA}(\varphi),$$

$$-X_A + X_B + F_{dh}(\varphi)\cdot\frac{x_M(\varphi) - x_N(\varphi)}{MN(\varphi)} = m_2\cdot a_{G2x}(\varphi),$$

$$-Y_A + Y_B - m_2\cdot g + F_{dh}(\varphi)\cdot\frac{y_M(\varphi) - y_N(\varphi)}{MN(\varphi)} = m_2\cdot a_{G2y}(\varphi),$$

$$\left(x_A(\varphi) - x_{G2}(\varphi)\right)\cdot\left(-Y_A\right) - \left(y_A(\varphi) - y_{G2}(\varphi)\right)\cdot\left(-X_A\right) + \left(x_B(\varphi) - x_{G2}(\varphi)\right)\cdot Y_B - \left(y_B(\varphi) - y_{G2}(\varphi)\right)\cdot X_B + \left(x_N(\varphi) - x_{G2}(\varphi)\right)\cdot F_{dh}(\varphi)\cdot\frac{y_M(\varphi) - y_N(\varphi)}{MN(\varphi)} - \left(y_N(\varphi) - y_{G2}(\varphi)\right)\cdot F_{dh}(\varphi)\cdot\frac{x_M(\varphi) - x_N(\varphi)}{MN(\varphi)} = J_2\cdot\varepsilon_{AB}(\varphi),$$

$$Y_B + m_3\cdot g = N_B,$$

$$-\mu\cdot\left|N_B\right|\cdot\frac{v_B(\varphi)}{\left|v_B(\varphi)\right| + 10^{-6}} = F_{ms},$$

$$\left.-X_B + F_{ms} + F(i) = m_3\cdot a_B(\varphi)\right\}:$$

$fsolve\left(Sys\_dynamic, \{X_A, Y_A, X_{O}, Y_{O}, X_B, Y_B, M, N_B, F_{ms}\}\right) : assign(\%);$

$XX_{O}(i, 1) := i; XX_A(i, 1) := i; XX_B(i, 1) = i; YY_{O}(i, 1) := i; YY_A(i, 1) := i; YY_B(i, 1) = i; MM(i, 1) := i; NN_B(i, 1) := i; FF_{ms}(i, 1) := i; RR_{O}(i, 1) := i; RR_A(i, 1) := i; RR_B(i, 1) := i;$

$XX_{O}(i, 2) := X_{O}; XX_A(i, 2) := X_A; XX_B(i, 2) = X_B; YY_{O}(i, 2) := Y_{O}; YY_A(i, 2) := Y_A; YY_B(i, 2) = Y_B; MM(i, 2) := M; NN_B(i, 2) := N_B; FF_{ms}(i, 2) := F_{ms};$

$RR_{O}(i, 2) := \sqrt{\left(X_{O}\right)^2 + \left(Y_{O}\right)^2};$

$RR_A(i, 2) := \sqrt{\left(X_A\right)^2 + \left(Y_A\right)^2};$

$RR_B(i, 2) := \sqrt{\left(X_B\right)^2 + \left(Y_B\right)^2};$

$unassign\left('X_{O}','X_A','X_B','Y_{O}','Y_A','Y_B','M','N_B','F_{ms}'\right);$

od:

return $\max\left(\left|NN_B\right|\right);$

end proc:

$NB\_10000 :=$ **proc**$(x1, x2)$

local $\alpha_1, \alpha_2, k, \varphi, \varepsilon_{OA}, l_1, l_2, \omega_{OA}, m_1, m_2, m_3, g, \mu, F, x_A, y_A, x_B, y_B, x_{G1}, y_{G1}, x_{G2}, y_{G2}, x_O, y_O, x_M, y_M, x_N, y_N, l_0, MN, \Delta l, F_{dh}, \varepsilon_{AB}, v_B, a_B, a_{G1x}, a_{G1y}, a_{G2x}, a_{G2y}, Sys\_dynamic, X_A, Y_A, X_O, Y_O, X_B, Y_B, M, N_B, F_{ms}, n, XX_O, XX_A, XX_B, YY_O, YY_A, YY_B, MM, NN_B, FF_{ms}, i, RR_O, RR_A, RR_B, J_1, J_2, AG_2, AG_1$;

#if not is({args}, set(numeric)) **then return** *('procname')('args')* **end if;**

$\alpha_1 := x1;\ \alpha_2 := x2;\ k := 10000;$

$l_1 := 0.175;\ l_2 := 0.58;\ \omega_{OA} := 3.1416;\ m_1 := 41.5147;\ m_2 := 7.656875;\ m_3 := 9.8996;\ g := 9.81;\ \mu := 0.3;\ AG_2 := 0.3165732;\ AG_1 := 0.0019;$

$J_1 := 0.889678;$

$J_2 := 0.6204482568;$

$x_A := \varphi \rightarrow l_1 \cdot \cos(\varphi);$

$y_A := \varphi \rightarrow l_1 \cdot \sin(\varphi);$

$$x_B := \varphi \rightarrow l_1 \cdot \cos(\varphi) + \left(l_2^{\,2} - \left(l_1 \cdot \sin(\varphi)\right)^2\right)^{\frac{1}{2}};$$

$y_B := \varphi \rightarrow 0;$

$x_{G1} := \varphi \rightarrow AG_1 \cdot \cos(\varphi);$

$y_{G1} := \varphi \rightarrow AG_1 \cdot \sin(\varphi);$

$$x_{G2} := \varphi \rightarrow x_A(\varphi) + AG_2 \cdot \sqrt{1 - \frac{l_1^{\,2}}{l_2^{\,2}} \cdot (\sin(\varphi))^2};$$

$$y_{G2} := \varphi \rightarrow \left(1 - \frac{AG_2}{l_2}\right) \cdot y_A(\varphi);$$

$x_O := \varphi \rightarrow 0;$

$y_O := \varphi \rightarrow 0;$

$$v_B := \varphi \rightarrow -\left(1 + \frac{l_1 \cdot \cos(\varphi)}{\left(l_2^{\,2} - l_1^{\,2} \cdot (\sin(\varphi))^2\right)^{\frac{1}{2}}}\right) \cdot \omega_{OA} \cdot l_1 \cdot \sin(\varphi);$$

$x_M := \varphi \rightarrow \alpha_1 \cdot l_1 \cdot \cos(\varphi);$

$y_M := \varphi \rightarrow \alpha_1 \cdot l_1 \cdot \sin(\varphi);$

$$x_N := \varphi \rightarrow l_1 \cdot \cos(\varphi) + \alpha_2 \cdot \sqrt{l_2^{\,2} - l_1^{\,2} \cdot (\sin(\varphi))^2};$$

$y_N := \varphi \rightarrow (1 - \alpha_2) \cdot l_1 \cdot \sin(\varphi);$

$l_0 := \varphi \rightarrow (1 - \alpha_1) \cdot l_1 + \alpha_2 \cdot l_2;$

$$MN := \varphi \rightarrow 10^{-10} + \sqrt{(x_M(\varphi) - x_N(\varphi))^2 + (y_M(\varphi) - y_N(\varphi))^2};$$

$\Delta l := \varphi \rightarrow MN(\varphi) - l_0(\varphi);$

$F_{dh} := \varphi \rightarrow k \cdot \Delta l(\varphi);$

$\varepsilon_{OA} := \varphi \rightarrow 0;$

$$\varepsilon_{AB} := \varphi \rightarrow \frac{l_2^{\,2} - l_1^{\,2}}{\left(l_2^{\,2} - l_1^{\,2}\cdot(\sin(\varphi))^2\right)^{\frac{3}{2}}}\cdot(\omega_{OA})^2\cdot l_1\cdot\sin(\varphi) - \frac{l_1\cdot\cos(\varphi)}{\left(l_2^{\,2} - l_1^{\,2}\cdot(\sin(\varphi))^2\right)^{\frac{1}{2}}}\cdot\varepsilon_{OA}(\varphi);$$

$$a_B := \varphi \rightarrow -\varepsilon_{OA}(\varphi)\cdot l_1\cdot\sin(\varphi)\cdot\left(1 + \frac{l_1\cdot\cos(\varphi)}{\left(l_2^{\,2} - l_1^{\,2}\cdot(\sin(\varphi))^2\right)^{\frac{1}{2}}}\right) + l_1\cdot\omega_{OA}^{\;2}$$
$$\cdot\left(\frac{l_2^{\,2} - l_1^{\,2}}{\left(l_2^{\,2} - l_1^{\,2}\cdot(\sin(\varphi))^2\right)^{\frac{3}{2}}}\cdot l_1\cdot(\sin(\varphi))^2 - \cos(\varphi) - \frac{l_1\cdot(\cos(\varphi))^2}{\left(l_2^{\,2} - l_1^{\,2}\cdot(\sin(\varphi))^2\right)^{\frac{1}{2}}}\right);$$

$$a_{G1x} := \varphi \rightarrow -AG_1\cdot\left(\varepsilon_{OA}(\varphi)\cdot\sin(\varphi) + \omega_{OA}^{\;2}\cdot\cos(\varphi)\right);$$

$$a_{G1y} := \varphi \rightarrow AG_1\cdot\left(\varepsilon_{OA}(\varphi)\cdot\cos(\varphi) - \omega_{OA}^{\;2}\cdot\sin(\varphi)\right);$$

$$\#a_{G2x} := \varphi \rightarrow -l_1\sin(\varphi)\left(1 + \frac{AG_2\,l_1\cos(\varphi)}{\sqrt{1 - \frac{l_1^{\,2}\sin(\varphi)^2}{l_2^{\,2}}}\;l_2^{\,2}}\right)\varepsilon_{OA}(\varphi) - l_1\,\omega_{OA}^{\;2}\left(\cos(\varphi)\right.$$
$$\left. + \frac{AG_2\,l_1^{\,3}\sin(\varphi)^2\cos(\varphi)^2}{\left(1 - \frac{l_1^{\,2}\sin(\varphi)^2}{l_2^{\,2}}\right)^{3/2} l_2^{\,4}} + \frac{AG_2\,l_1\cos(2\,\varphi)}{\sqrt{1 - \frac{l_1^{\,2}\sin(\varphi)^2}{l_2^{\,2}}}\;l_2^{\,2}}\right);$$

$$a_{G2x} := \varphi \rightarrow -l_1\left(\varepsilon_{OA}(\varphi)\right)\sin(\varphi) - l_1\left(\omega_{OA}\right)^2\cos(\varphi) - \frac{AG_2\,l_1^{\,4}\sin(\varphi)^2\left(\omega_{OA}\right)^2\cos(\varphi)^2}{\left(1 - \frac{l_1^{\,2}\sin(\varphi)^2}{l_2^{\,2}}\right)^{3/2} l_2^{\,4}}$$
$$- \frac{AG_2\,l_1^{\,2}\left(\omega_{OA}\right)^2\cos(\varphi)^2}{\sqrt{1 - \frac{l_1^{\,2}\sin(\varphi)^2}{l_2^{\,2}}}\;l_2^{\,2}} - \frac{AG_2\,l_1^{\,2}\sin(\varphi)\left(\varepsilon_{OA}(\varphi)\right)\cos(\varphi)}{\sqrt{1 - \frac{l_1^{\,2}\sin(\varphi)^2}{l_2^{\,2}}}\;l_2^{\,2}} + \frac{AG_2\,l_1^{\,2}\sin(\varphi)^2\left(\omega_{OA}\right)^2}{\sqrt{1 - \frac{l_1^{\,2}\sin(\varphi)^2}{l_2^{\,2}}}\;l_2^{\,2}};$$

$$\#a_{G2y} := \varphi \rightarrow \frac{\left(-l_2 + AG_2\right) l_1\left(\omega_{OA}^{\;2}\sin(\varphi) - \varepsilon_{OA}(\varphi)\cos(\varphi)\right)}{l_2};$$

$$a_{G2y} := \varphi \rightarrow \left(1 - \frac{AG_2}{l_2}\right) l_1\left(\varepsilon_{OA}(\varphi)\right)\cos(\varphi) - \left(1 - \frac{AG_2}{l_2}\right) l_1\left(\omega_{OA}\right)^2\sin(\varphi);$$

$n := 360;$

$XX_{\mathrm{O}} := Matrix(n, 2):$

$XX_A := Matrix(n, 2):$

$YY_{\mathrm{O}} := Matrix(n, 2):$

$YY_A := Matrix(n, 2):$

$MM := Matrix(n, 2):$

$XX_B := Matrix(n, 2):$

$YY_B := Matrix(n, 2):$

$NN_B := Matrix(n, 2):$

$FF_{ms} := Matrix(n, 2) :$
$RR_{O} := Matrix(n, 2) :$
$RR_{A} := Matrix(n, 2) :$
$RR_{B} := Matrix(n, 2) :$
$F := Matrix(n, 1) :$
for *i* **from** 271 **to** 360 **do**
$F[i] := -3233;$
od:
for *i* **from** 1 **by** 1 **to** *n* **do**

$$\varphi := \frac{(i-1)\cdot\pi}{180};$$

$$Sys\_dynamic := \Bigg\{ X_O + X_A + F_{dh}(\varphi)\cdot\frac{x_N(\varphi) - x_M(\varphi)}{MN(\varphi)} = m_1\cdot a_{G1x}(\varphi),$$

$$Y_O + Y_A - m_1\cdot g + F_{dh}(\varphi)\cdot\frac{y_N(\varphi) - y_M(\varphi)}{MN(\varphi)} = m_1\cdot a_{G1y}(\varphi),$$

$$M + \big(x_A(\varphi) - x_{G1}(\varphi)\big)\cdot Y_A - \big(y_A(\varphi) - y_{G1}(\varphi)\big)\cdot X_A + \big(x_O(\varphi) - x_{G1}(\varphi)\big)\cdot Y_O - \big(y_O(\varphi) - y_{G1}(\varphi)\big)\cdot X_O + \big(x_M(\varphi) - x_{G1}(\varphi)\big)\cdot F_{dh}(\varphi)\cdot\frac{y_N(\varphi) - y_M(\varphi)}{MN(\varphi)} - \big(y_M(\varphi) - y_{G1}(\varphi)\big)\cdot F_{dh}(\varphi)\cdot\frac{x_N(\varphi) - x_M(\varphi)}{MN(\varphi)} = J_1\cdot\varepsilon_{OA}(\varphi),$$

$$-X_A + X_B + F_{dh}(\varphi)\cdot\frac{x_M(\varphi) - x_N(\varphi)}{MN(\varphi)} = m_2\cdot a_{G2x}(\varphi),$$

$$-Y_A + Y_B - m_2\cdot g + F_{dh}(\varphi)\cdot\frac{y_M(\varphi) - y_N(\varphi)}{MN(\varphi)} = m_2\cdot a_{G2y}(\varphi),$$

$$\big(x_A(\varphi) - x_{G2}(\varphi)\big)\cdot(-Y_A) - \big(y_A(\varphi) - y_{G2}(\varphi)\big)\cdot(-X_A) + \big(x_B(\varphi) - x_{G2}(\varphi)\big)\cdot Y_B - \big(y_B(\varphi) - y_{G2}(\varphi)\big)\cdot X_B + \big(x_N(\varphi) - x_{G2}(\varphi)\big)\cdot F_{dh}(\varphi)\cdot\frac{y_M(\varphi) - y_N(\varphi)}{MN(\varphi)} - \big(y_N(\varphi) - y_{G2}(\varphi)\big)\cdot F_{dh}(\varphi)\cdot\frac{x_M(\varphi) - x_N(\varphi)}{MN(\varphi)} = J_2\cdot\varepsilon_{AB}(\varphi),$$

$$Y_B + m_3\cdot g = N_B,$$

$$-\mu\cdot|N_B|\cdot\frac{v_B(\varphi)}{|v_B(\varphi)| + 10^{-6}} = F_{ms},$$

$$-X_B + F_{ms} + F(i) = m_3\cdot a_B(\varphi) \Bigg\}:$$

$fsolve\big(Sys\_dynamic, \{X_A, Y_A, X_O, Y_O, X_B, Y_B, M, N_B, F_{ms}\}\big) : assign(\%);$

$XX_{O}(i,1) := i; XX_{A}(i,1) := i; XX_{B}(i,1) = i; YY_{O}(i,1) := i; YY_{A}(i,1) := i; YY_{B}(i,1) = i; MM(i,1) := i; NN_{B}(i,1) := i; FF_{ms}(i,1) := i; RR_{O}(i,1) := i; RR_{A}(i,1) := i; RR_{B}(i,1) := i;$

$XX_{O}(i,2) := X_{O}; XX_{A}(i,2) := X_{A}; XX_{B}(i,2) = X_{B}; YY_{O}(i,2) := Y_{O}; YY_{A}(i,2) := Y_{A}; YY_{B}(i,2) = Y_{B}; MM(i,2) := M; NN_{B}(i,2) := N_{B}; FF_{ms}(i,2) := F_{ms};$

$RR_{O}(i,2) := \sqrt{(X_{O})^2 + (Y_{O})^2};$

$RR_{A}(i,2) := \sqrt{(X_{A})^2 + (Y_{A})^2};$

$RR_{B}(i,2) := \sqrt{(X_{B})^2 + (Y_{B})^2};$

$unassign('X_{O}','X_{A}','X_{B}','Y_{O}','Y_{A}','Y_{B}','M','N_{B}','F_{ms}');$

od:

return $\max(|NN_{B}|);$

end proc:

$NB\_15000 :=$ **proc**$(x1, x2)$

local $\alpha_1, \alpha_2, k, \varphi, \varepsilon_{OA}, l_1, l_2, \omega_{OA}, m_1, m_2, m_3, g, \mu, F, x_A, y_A, x_B, y_B, x_{G1}, y_{G1}, x_{G2}, y_{G2}, x_O, y_O, x_M, y_M, x_N, y_N, l_0, MN, \Delta l, F_{dh}, \varepsilon_{AB}, v_B, a_B, a_{G1x}, a_{G1y}, a_{G2x}, a_{G2y}, Sys\_dynamic, X_A, Y_A, X_O, Y_O, X_B, Y_B, M, N_B, F_{ms}, n, XX_O, XX_A, XX_B, YY_O, YY_A, YY_B, MM, NN_B, FF_{ms}, i, RR_O, RR_A, RR_B, J_1, J_2, AG_2, AG_1;$

#if not is({args}, set(numeric)) **then return** *('procname')('args')* **end if**;

$\alpha_1 := x1; \alpha_2 := x2; k := 15000;$

$l_1 := 0.175; l_2 := 0.58; \omega_{OA} := 3.1416; m_1 := 41.5147; m_2 := 7.656875; m_3 := 9.8996; g := 9.81; \mu := 0.3; AG_2 := 0.3165732; AG_1 := 0.0019;$

$J_1 := 0.889678;$

$J_2 := 0.6204482568;$

$x_A := \varphi \to l_1 \cdot \cos(\varphi);$

$y_A := \varphi \to l_1 \cdot \sin(\varphi);$

$x_B := \varphi \to l_1 \cdot \cos(\varphi) + \left(l_2^2 - (l_1 \cdot \sin(\varphi))^2\right)^{\frac{1}{2}};$

$y_B := \varphi \to 0;$

$x_{G1} := \varphi \to AG_1 \cdot \cos(\varphi);$

$y_{G1} := \varphi \to AG_1 \cdot \sin(\varphi);$

$x_{G2} := \varphi \to x_A(\varphi) + AG_2 \cdot \sqrt{1 - \frac{l_1^2}{l_2^2} \cdot (\sin(\varphi))^2};$

$y_{G2} := \varphi \to \left(1 - \frac{AG_2}{l_2}\right) \cdot y_A(\varphi);$

$x_O := \varphi \to 0;$

$y_O := \varphi \to 0;$

$v_B := \varphi \to -\left(1 + \frac{l_1 \cdot \cos(\varphi)}{\left(l_2^2 - l_1^2 \cdot (\sin(\varphi))^2\right)^{\frac{1}{2}}}\right) \cdot \omega_{OA} \cdot l_1 \cdot \sin(\varphi);$

$$x_M := \varphi \to \alpha_1 \cdot l_1 \cdot \cos(\varphi);$$
$$y_M := \varphi \to \alpha_1 \cdot l_1 \cdot \sin(\varphi);$$
$$x_N := \varphi \to l_1 \cdot \cos(\varphi) + \alpha_2 \cdot \sqrt{l_2^2 - l_1^2 \cdot (\sin(\varphi))^2};$$
$$y_N := \varphi \to \left(1 - \alpha_2\right) \cdot l_1 \cdot \sin(\varphi);$$
$$l_0 := \varphi \to \left(1 - \alpha_1\right) \cdot l_1 + \alpha_2 \cdot l_2;$$
$$MN := \varphi \to 10^{-10} + \sqrt{\left(x_M(\varphi) - x_N(\varphi)\right)^2 + \left(y_M(\varphi) - y_N(\varphi)\right)^2};$$
$$\Delta l := \varphi \to MN(\varphi) - l_0(\varphi);$$
$$F_{dh} := \varphi \to k \cdot \Delta l(\varphi);$$
$$\varepsilon_{OA} := \varphi \to 0;$$
$$\varepsilon_{AB} := \varphi \to \frac{l_2^2 - l_1^2}{\left(l_2^2 - l_1^2 \cdot (\sin(\varphi))^2\right)^{\frac{3}{2}}} \cdot \left(\omega_{OA}\right)^2 \cdot l_1 \cdot \sin(\varphi) - \frac{l_1 \cdot \cos(\varphi)}{\left(l_2^2 - l_1^2 \cdot (\sin(\varphi))^2\right)^{\frac{1}{2}}} \cdot \varepsilon_{OA}(\varphi);$$
$$a_B := \varphi \to -\varepsilon_{OA}(\varphi) \cdot l_1 \cdot \sin(\varphi) \cdot \left(1 + \frac{l_1 \cdot \cos(\varphi)}{\left(l_2^2 - l_1^2 \cdot (\sin(\varphi))^2\right)^{\frac{1}{2}}}\right) + l_1 \cdot \omega_{OA}^2 \cdot \left(\frac{l_2^2 - l_1^2}{\left(l_2^2 - l_1^2 \cdot (\sin(\varphi))^2\right)^{\frac{3}{2}}} \cdot l_1 \cdot (\sin(\varphi))^2 - \cos(\varphi) - \frac{l_1 \cdot (\cos(\varphi))^2}{\left(l_2^2 - l_1^2 \cdot (\sin(\varphi))^2\right)^{\frac{1}{2}}}\right);$$
$$a_{G1x} := \varphi \to -AG_1 \cdot \left(\varepsilon_{OA}(\varphi) \cdot \sin(\varphi) + \omega_{OA}^2 \cdot \cos(\varphi)\right);$$
$$a_{G1y} := \varphi \to AG_1 \cdot \left(\varepsilon_{OA}(\varphi) \cdot \cos(\varphi) - \omega_{OA}^2 \cdot \sin(\varphi)\right);$$
$$\#a_{G2x} := \varphi \to -l_1 \sin(\varphi) \left(1 + \frac{AG_2\, l_1 \cos(\varphi)}{\sqrt{1 - \frac{l_1^2 \sin(\varphi)^2}{l_2^2}}\; l_2^2}\right) \varepsilon_{OA}(\varphi) - l_1\, \omega_{OA}^2 \left(\cos(\varphi) + \frac{AG_2\, l_1^3 \sin(\varphi)^2 \cos(\varphi)^2}{\left(1 - \frac{l_1^2 \sin(\varphi)^2}{l_2^2}\right)^{3/2} l_2^4} + \frac{AG_2\, l_1 \cos(2\varphi)}{\sqrt{1 - \frac{l_1^2 \sin(\varphi)^2}{l_2^2}}\; l_2^2}\right);$$
$$a_{G2x} := \varphi \to -l_1 \left(\varepsilon_{OA}(\varphi)\right) \sin(\varphi) - l_1 \left(\omega_{OA}\right)^2 \cos(\varphi) - \frac{AG_2\, l_1^4 \sin(\varphi)^2 \left(\omega_{OA}\right)^2 \cos(\varphi)^2}{\left(1 - \frac{l_1^2 \sin(\varphi)^2}{l_2^2}\right)^{3/2} l_2^4} - \frac{AG_2\, l_1^2 \left(\omega_{OA}\right)^2 \cos(\varphi)^2}{\sqrt{1 - \frac{l_1^2 \sin(\varphi)^2}{l_2^2}}\; l_2^2} - \frac{AG_2\, l_1^2 \sin(\varphi) \left(\varepsilon_{OA}(\varphi)\right) \cos(\varphi)}{\sqrt{1 - \frac{l_1^2 \sin(\varphi)^2}{l_2^2}}\; l_2^2} + \frac{AG_2\, l_1^2 \sin(\varphi)^2 \left(\omega_{OA}\right)^2}{\sqrt{1 - \frac{l_1^2 \sin(\varphi)^2}{l_2^2}}\; l_2^2};$$
$$\#a_{G2y} := \varphi \to \frac{\left(-l_2 + AG_2\right) l_1 \left(\omega_{OA}^2 \sin(\varphi) - \varepsilon_{OA}(\varphi) \cos(\varphi)\right)}{l_2};$$

$a_{G2y} := \varphi \rightarrow \left(1 - \frac{AG_2}{l_2}\right) l_1 \left(\varepsilon_{OA}(\varphi)\right) \cos(\varphi) - \left(1 - \frac{AG_2}{l_2}\right) l_1 \left(\omega_{OA}\right)^2 \sin(\varphi);$

$n := 360;$

$XX_O := Matrix(n, 2) :$

$XX_A := Matrix(n, 2) :$

$YY_O := Matrix(n, 2) :$

$YY_A := Matrix(n, 2) :$

$MM := Matrix(n, 2) :$

$XX_B := Matrix(n, 2) :$

$YY_B := Matrix(n, 2) :$

$NN_B := Matrix(n, 2) :$

$FF_{ms} := Matrix(n, 2) :$

$RR_O := Matrix(n, 2) :$

$RR_A := Matrix(n, 2) :$

$RR_B := Matrix(n, 2) :$

$F := Matrix(n, 1) :$

for *i* **from** 271 **to** 360 **do**

$F[i] := -3233;$

od:

for *i* **from** 1 **by** 1 **to** *n* **do**

$\varphi := \frac{(i-1)\cdot\pi}{180};$

$Sys\_dynamic := \Bigg\{$

$X_O + X_A + F_{dh}(\varphi)\cdot\frac{x_N(\varphi) - x_M(\varphi)}{MN(\varphi)} = m_1\cdot a_{G1x}(\varphi),$

$Y_O + Y_A - m_1\cdot g + F_{dh}(\varphi)\cdot\frac{y_N(\varphi) - y_M(\varphi)}{MN(\varphi)} = m_1\cdot a_{G1y}(\varphi),$

$M + \left(x_A(\varphi) - x_{G1}(\varphi)\right)\cdot Y_A - \left(y_A(\varphi) - y_{G1}(\varphi)\right)\cdot X_A + \left(x_O(\varphi) - x_{G1}(\varphi)\right)\cdot Y_O - \left(y_O(\varphi) - y_{G1}(\varphi)\right)$
$\cdot X_O + \left(x_M(\varphi) - x_{G1}(\varphi)\right)\cdot F_{dh}(\varphi)\cdot\frac{y_N(\varphi) - y_M(\varphi)}{MN(\varphi)} - \left(y_M(\varphi) - y_{G1}(\varphi)\right)\cdot F_{dh}(\varphi)$
$\cdot\frac{x_N(\varphi) - x_M(\varphi)}{MN(\varphi)} = J_1\cdot\varepsilon_{OA}(\varphi),$

$-X_A + X_B + F_{dh}(\varphi)\cdot\frac{x_M(\varphi) - x_N(\varphi)}{MN(\varphi)} = m_2\cdot a_{G2x}(\varphi),$

$-Y_A + Y_B - m_2\cdot g + F_{dh}(\varphi)\cdot\frac{y_M(\varphi) - y_N(\varphi)}{MN(\varphi)} = m_2\cdot a_{G2y}(\varphi),$

$\left(x_A(\varphi) - x_{G2}(\varphi)\right)\cdot\left(-Y_A\right) - \left(y_A(\varphi) - y_{G2}(\varphi)\right)\cdot\left(-X_A\right) + \left(x_B(\varphi) - x_{G2}(\varphi)\right)\cdot Y_B - \left(y_B(\varphi)\right.$

$- y_{G2}(\varphi)\big)\cdot X_B + \big(x_N(\varphi) - x_{G2}(\varphi)\big)\cdot F_{dh}(\varphi)\cdot \dfrac{y_M(\varphi) - y_N(\varphi)}{MN(\varphi)} - \big(y_N(\varphi) - y_{G2}(\varphi)\big)\cdot F_{dh}(\varphi)$

$\cdot \dfrac{x_M(\varphi) - x_N(\varphi)}{MN(\varphi)} = J_2\cdot\varepsilon_{AB}(\varphi),$

$Y_B + m_3\cdot g = N_B,$

$-\mu\cdot|N_B|\cdot \dfrac{v_B(\varphi)}{|v_B(\varphi)| + 10^{-6}} = F_{ms},$

$\left.-X_B + F_{ms} + F(i) = m_3\cdot a_B(\varphi)\right\}:$

$fsolve\big(Sys\_dynamic, \{X_A, Y_A, X_O, Y_O, X_B, Y_B, M, N_B, F_{ms}\}\big) : assign(\%);$

$XX_O(i,1) := i; XX_A(i,1) := i; XX_B(i,1) = i; YY_O(i,1) := i; YY_A(i,1) := i; YY_B(i,1) = i; MM(i,1) :=$
$i; NN_B(i,1) := i; FF_{ms}(i,1) := i; RR_O(i,1) := i; RR_A(i,1) := i; RR_B(i,1) := i;$

$XX_O(i,2) := X_O; XX_A(i,2) := X_A; XX_B(i,2) = X_B; YY_O(i,2) := Y_O; YY_A(i,2) := Y_A; YY_B(i,2) = Y_B;$
$MM(i,2) := M; NN_B(i,2) := N_B; FF_{ms}(i,2) := F_{ms};$

$RR_O(i,2) := \sqrt{(X_O)^2 + (Y_O)^2};$

$RR_A(i,2) := \sqrt{(X_A)^2 + (Y_A)^2};$

$RR_B(i,2) := \sqrt{(X_B)^2 + (Y_B)^2};$

$unassign('X_O','X_A','X_B','Y_O','Y_A','Y_B','M','N_B','F_{ms}');$

od:

return $\max(|NN_B|);$

end proc:

$NB\_20000 :=$ **proc**$(x1, x2)$

local $\alpha_1, \alpha_2, k, \varphi, \varepsilon_{OA}, l_1, l_2, \omega_{OA}, m_1, m_2, m_3, g, \mu, F, x_A, y_A, x_B, y_B, x_{G1}, y_{G1}, x_{G2}, y_{G2}, x_O, y_O, x_M, y_M, x_N, y_N,$
$l_0, MN, \Delta l, F_{dh}, \varepsilon_{AB}, v_B, a_B, a_{G1x}, a_{G1y}, a_{G2x}, a_{G2y}, Sys\_dynamic, X_A, Y_A, X_O, Y_O, X_B, Y_B, M, N_B, F_{ms}, n,$
$XX_O, XX_A, XX_B, YY_O, YY_A, YY_B, MM, NN_B, FF_{ms}, i, RR_O, RR_A, RR_B, J_1, J_2, AG_2, AG_1;$

#if not is$(\{args\}, set(numeric))$ **then return** $('procname')('args')$ **end if;**

$\alpha_1 := x1; \alpha_2 := x2; k := 20000;$

$l_1 := 0.175; l_2 := 0.58; \omega_{OA} := 3.1416; m_1 := 41.5147; m_2 := 7.656875; m_3 := 9.8996; g := 9.81; \mu :=$
$0.3;\ AG_2 := 0.3165732; AG_1 := 0.0019;$

$J_1 := 0.889678;$

$J_2 := 0.6204482568;$

$x_A := \varphi \to l_1\cdot\cos(\varphi);$

$y_A := \varphi \to l_1\cdot\sin(\varphi);$

$x_B := \varphi \to l_1\cdot\cos(\varphi) + \left(l_2^{\,2} - (l_1\cdot\sin(\varphi))^2\right)^{\frac{1}{2}};$

$y_B := \varphi \to 0;$

$$x_{G1} := \varphi \to AG_1 \cdot \cos(\varphi);$$

$$y_{G1} := \varphi \to AG_1 \cdot \sin(\varphi);$$

$$x_{G2} := \varphi \to x_A(\varphi) + AG_2 \cdot \sqrt{1 - \frac{l_1^2}{l_2^2} \cdot (\sin(\varphi))^2};$$

$$y_{G2} := \varphi \to \left(1 - \frac{AG_2}{l_2}\right) \cdot y_A(\varphi);$$

$$x_{\mathrm{O}} := \varphi \to 0;$$

$$y_{\mathrm{O}} := \varphi \to 0;$$

$$v_B := \varphi \to -\left(1 + \frac{l_1 \cdot \cos(\varphi)}{\left(l_2^2 - l_1^2 \cdot (\sin(\varphi))^2\right)^{\frac{1}{2}}}\right) \cdot \omega_{OA} \cdot l_1 \cdot \sin(\varphi);$$

$$x_M := \varphi \to \alpha_1 \cdot l_1 \cdot \cos(\varphi);$$

$$y_M := \varphi \to \alpha_1 \cdot l_1 \cdot \sin(\varphi);$$

$$x_N := \varphi \to l_1 \cdot \cos(\varphi) + \alpha_2 \cdot \sqrt{l_2^2 - l_1^2 \cdot (\sin(\varphi))^2};$$

$$y_N := \varphi \to \left(1 - \alpha_2\right) \cdot l_1 \cdot \sin(\varphi);$$

$$l_0 := \varphi \to \left(1 - \alpha_1\right) \cdot l_1 + \alpha_2 \cdot l_2;$$

$$MN := \varphi \to 10^{-10} + \sqrt{\left(x_M(\varphi) - x_N(\varphi)\right)^2 + \left(y_M(\varphi) - y_N(\varphi)\right)^2};$$

$$\Delta l := \varphi \to MN(\varphi) - l_0(\varphi);$$

$$F_{dh} := \varphi \to k \cdot \Delta l(\varphi);$$

$$\varepsilon_{OA} := \varphi \to 0;$$

$$\varepsilon_{AB} := \varphi \to \frac{l_2^2 - l_1^2}{\left(l_2^2 - l_1^2 \cdot (\sin(\varphi))^2\right)^{\frac{3}{2}}} \cdot \left(\omega_{OA}\right)^2 \cdot l_1 \cdot \sin(\varphi) - \frac{l_1 \cdot \cos(\varphi)}{\left(l_2^2 - l_1^2 \cdot (\sin(\varphi))^2\right)^{\frac{1}{2}}} \cdot \varepsilon_{OA}(\varphi);$$

$$a_B := \varphi \to -\varepsilon_{OA}(\varphi) \cdot l_1 \cdot \sin(\varphi) \cdot \left(1 + \frac{l_1 \cdot \cos(\varphi)}{\left(l_2^2 - l_1^2 \cdot (\sin(\varphi))^2\right)^{\frac{1}{2}}}\right) + l_1 \cdot \omega_{OA}^{\;2}$$
$$\cdot \left(\frac{l_2^2 - l_1^2}{\left(l_2^2 - l_1^2 \cdot (\sin(\varphi))^2\right)^{\frac{3}{2}}} \cdot l_1 \cdot (\sin(\varphi))^2 - \cos(\varphi) - \frac{l_1 \cdot (\cos(\varphi))^2}{\left(l_2^2 - l_1^2 \cdot (\sin(\varphi))^2\right)^{\frac{1}{2}}}\right);$$

$$a_{G1x} := \varphi \to -AG_1 \cdot \left(\varepsilon_{OA}(\varphi) \cdot \sin(\varphi) + \omega_{OA}^{\;2} \cdot \cos(\varphi)\right);$$

$$a_{G1y} := \varphi \to AG_1 \cdot \left(\varepsilon_{OA}(\varphi) \cdot \cos(\varphi) - \omega_{OA}^{\;2} \cdot \sin(\varphi)\right);$$

$$\#a_{G2x} := \varphi \to -l_1 \sin(\varphi) \left(1 + \frac{AG_2\, l_1 \cos(\varphi)}{\sqrt{1 - \frac{l_1^2 \sin(\varphi)^2}{l_2^2}\; l_2^2}}\right) \varepsilon_{OA}(\varphi) - l_1\, \omega_{OA}^{\;2} \left(\cos(\varphi)\right.$$

$$+\frac{AG_2\, l_1^3 \sin(\varphi)^2 \cos(\varphi)^2}{\left(1-\frac{l_1^2 \sin(\varphi)^2}{l_2^2}\right)^{3/2} l_2^4} + \frac{AG_2\, l_1 \cos(2\,\varphi)}{\sqrt{1-\frac{l_1^2 \sin(\varphi)^2}{l_2^2}}\; l_2^2}\Bigg);$$

$$a_{G2x} := \varphi \rightarrow -l_1\left(\varepsilon_{OA}(\varphi)\right)\sin(\varphi) - l_1\left(\omega_{OA}\right)^2\cos(\varphi) - \frac{AG_2\, l_1^4 \sin(\varphi)^2 \left(\omega_{OA}\right)^2 \cos(\varphi)^2}{\left(1-\frac{l_1^2 \sin(\varphi)^2}{l_2^2}\right)^{3/2} l_2^4}$$

$$-\frac{AG_2\, l_1^2 \left(\omega_{OA}\right)^2 \cos(\varphi)^2}{\sqrt{1-\frac{l_1^2 \sin(\varphi)^2}{l_2^2}}\; l_2^2} - \frac{AG_2\, l_1^2 \sin(\varphi)\left(\varepsilon_{OA}(\varphi)\right)\cos(\varphi)}{\sqrt{1-\frac{l_1^2 \sin(\varphi)^2}{l_2^2}}\; l_2^2} + \frac{AG_2\, l_1^2 \sin(\varphi)^2 \left(\omega_{OA}\right)^2}{\sqrt{1-\frac{l_1^2 \sin(\varphi)^2}{l_2^2}}\; l_2^2};$$

$$\#a_{G2y} := \varphi \rightarrow \frac{\left(-l_2 + AG_2\right) l_1 \left(\omega_{OA}^2 \sin(\varphi) - \varepsilon_{OA}(\varphi)\cos(\varphi)\right)}{l_2};$$

$$a_{G2y} := \varphi \rightarrow \left(1-\frac{AG_2}{l_2}\right) l_1 \left(\varepsilon_{OA}(\varphi)\right)\cos(\varphi) - \left(1-\frac{AG_2}{l_2}\right) l_1 \left(\omega_{OA}\right)^2 \sin(\varphi);$$

$n := 360;$

$XX_{\mathrm{O}} := Matrix(n, 2) :$

$XX_A := Matrix(n, 2) :$

$YY_{\mathrm{O}} := Matrix(n, 2) :$

$YY_A := Matrix(n, 2) :$

$MM := Matrix(n, 2) :$

$XX_B := Matrix(n, 2) :$

$YY_B := Matrix(n, 2) :$

$NN_B := Matrix(n, 2) :$

$FF_{ms} := Matrix(n, 2) :$

$RR_{\mathrm{O}} := Matrix(n, 2) :$

$RR_A := Matrix(n, 2) :$

$RR_B := Matrix(n, 2) :$

$F := Matrix(n, 1) :$

for *i* **from** 271 **to** 360 **do**

$F[i] := -3233;$

od:

for *i* **from** 1 **by** 1 **to** *n* **do**

$$\varphi := \frac{(i-1)\cdot\pi}{180};$$

$Sys\_dynamic := \Bigg\{$

$$X_{\mathrm{O}} + X_{A} + F_{dh}(\varphi)\cdot\frac{x_{N}(\varphi) - x_{M}(\varphi)}{MN(\varphi)} = m_{1}\cdot a_{G1x}(\varphi),$$

$$Y_{\mathrm{O}} + Y_{A} - m_{1}\cdot g + F_{dh}(\varphi)\cdot\frac{y_{N}(\varphi) - y_{M}(\varphi)}{MN(\varphi)} = m_{1}\cdot a_{G1y}(\varphi),$$

$$M + \left(x_{A}(\varphi) - x_{G1}(\varphi)\right)\cdot Y_{A} - \left(y_{A}(\varphi) - y_{G1}(\varphi)\right)\cdot X_{A} + \left(x_{\mathrm{O}}(\varphi) - x_{G1}(\varphi)\right)\cdot Y_{\mathrm{O}} - \left(y_{\mathrm{O}}(\varphi) - y_{G1}(\varphi)\right)$$
$$\cdot X_{\mathrm{O}} + \left(x_{M}(\varphi) - x_{G1}(\varphi)\right)\cdot F_{dh}(\varphi)\cdot\frac{y_{N}(\varphi) - y_{M}(\varphi)}{MN(\varphi)} - \left(y_{M}(\varphi) - y_{G1}(\varphi)\right)\cdot F_{dh}(\varphi)$$
$$\cdot\frac{x_{N}(\varphi) - x_{M}(\varphi)}{MN(\varphi)} = J_{1}\cdot\varepsilon_{OA}(\varphi),$$

$$-X_{A} + X_{B} + F_{dh}(\varphi)\cdot\frac{x_{M}(\varphi) - x_{N}(\varphi)}{MN(\varphi)} = m_{2}\cdot a_{G2x}(\varphi),$$

$$-Y_{A} + Y_{B} - m_{2}\cdot g + F_{dh}(\varphi)\cdot\frac{y_{M}(\varphi) - y_{N}(\varphi)}{MN(\varphi)} = m_{2}\cdot a_{G2y}(\varphi),$$

$$\left(x_{A}(\varphi) - x_{G2}(\varphi)\right)\cdot\left(-Y_{A}\right) - \left(y_{A}(\varphi) - y_{G2}(\varphi)\right)\cdot\left(-X_{A}\right) + \left(x_{B}(\varphi) - x_{G2}(\varphi)\right)\cdot Y_{B} - \left(y_{B}(\varphi)\right.$$
$$\left. - y_{G2}(\varphi)\right)\cdot X_{B} + \left(x_{N}(\varphi) - x_{G2}(\varphi)\right)\cdot F_{dh}(\varphi)\cdot\frac{y_{M}(\varphi) - y_{N}(\varphi)}{MN(\varphi)} - \left(y_{N}(\varphi) - y_{G2}(\varphi)\right)\cdot F_{dh}(\varphi)$$
$$\cdot\frac{x_{M}(\varphi) - x_{N}(\varphi)}{MN(\varphi)} = J_{2}\cdot\varepsilon_{AB}(\varphi),$$

$$Y_{B} + m_{3}\cdot g = N_{B},$$

$$-\mu\cdot\left|N_{B}\right|\cdot\frac{v_{B}(\varphi)}{\left|v_{B}(\varphi)\right| + 10^{-6}} = F_{ms},$$

$$\left.-X_{B} + F_{ms} + F(i) = m_{3}\cdot a_{B}(\varphi)\right\}:$$

$fsolve\left(Sys\_dynamic, \{X_{A}, Y_{A}, X_{\mathrm{O}}, Y_{\mathrm{O}}, X_{B}, Y_{B}, M, N_{B}, F_{ms}\}\right) : assign(\%);$

$XX_{\mathrm{O}}(i,1) := i; XX_{A}(i,1) := i; XX_{B}(i,1) = i; YY_{\mathrm{O}}(i,1) := i; YY_{A}(i,1) := i; YY_{B}(i,1) = i; MM(i,1) := i; NN_{B}(i,1) := i; FF_{ms}(i,1) := i; RR_{\mathrm{O}}(i,1) := i; RR_{A}(i,1) := i; RR_{B}(i,1) := i;$

$XX_{\mathrm{O}}(i,2) := X_{\mathrm{O}}; XX_{A}(i,2) := X_{A}; XX_{B}(i,2) = X_{B}; YY_{\mathrm{O}}(i,2) := Y_{\mathrm{O}}; YY_{A}(i,2) := Y_{A}; YY_{B}(i,2) = Y_{B}; MM(i,2) := M; NN_{B}(i,2) := N_{B}; FF_{ms}(i,2) := F_{ms};$

$RR_{\mathrm{O}}(i,2) := \sqrt{\left(X_{\mathrm{O}}\right)^{2} + \left(Y_{\mathrm{O}}\right)^{2}};$

$RR_{A}(i,2) := \sqrt{\left(X_{A}\right)^{2} + \left(Y_{A}\right)^{2}};$

$RR_{B}(i,2) := \sqrt{\left(X_{B}\right)^{2} + \left(Y_{B}\right)^{2}};$

$unassign\left('X_{\mathrm{O}}','X_{A}','X_{B}','Y_{\mathrm{O}}','Y_{A}','Y_{B}','M','N_{B}','F_{ms}'\right);$

od:

return $\max\left(\left|NN_{B}\right|\right);$

end proc:

$c\_0 := plot3d(NB\_0, 0..1, 0..1);$

$c\_1000 := plot3d(NB\_1000, 0..1, 0..1);$
$c\_5000 := plot3d(NB\_5000, 0..1, 0..1);$
$c\_10000 := plot3d(NB\_10000, 0..1, 0..1);$
$c\_15000 := plot3d(NB\_15000, 0..1, 0..1);$
$c\_20000 := plot3d(NB\_20000, 0..1, 0..1);$
$plots{:}\text{-}display(c\_0, c\_1000, c\_5000, c\_10000, c\_15000, c\_20000);$

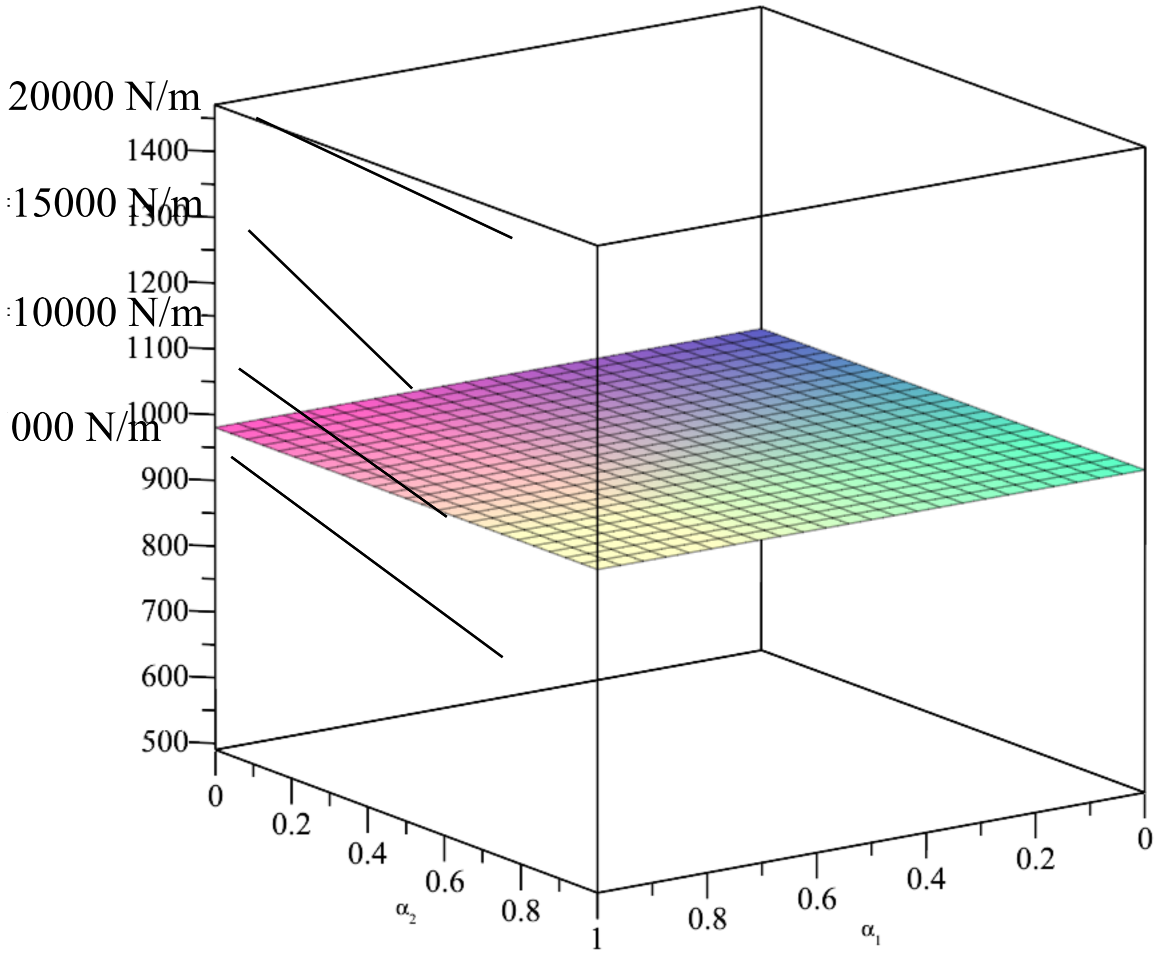

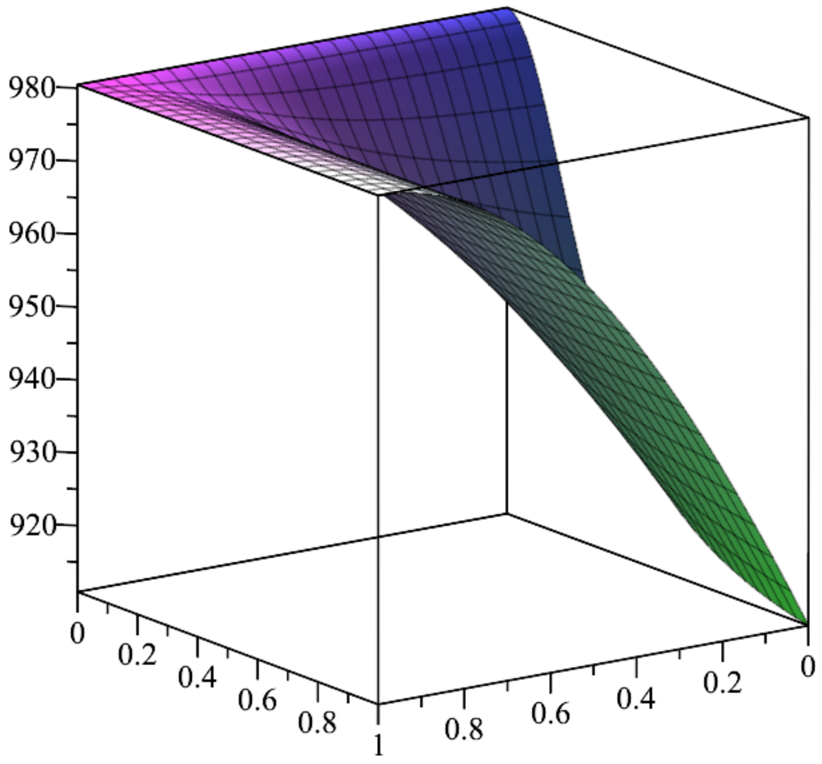
980
970
960
950
940
930
920
0
0.2
0.4
0.6
0.8
1
0.8
0.6
0.4
0.2
0

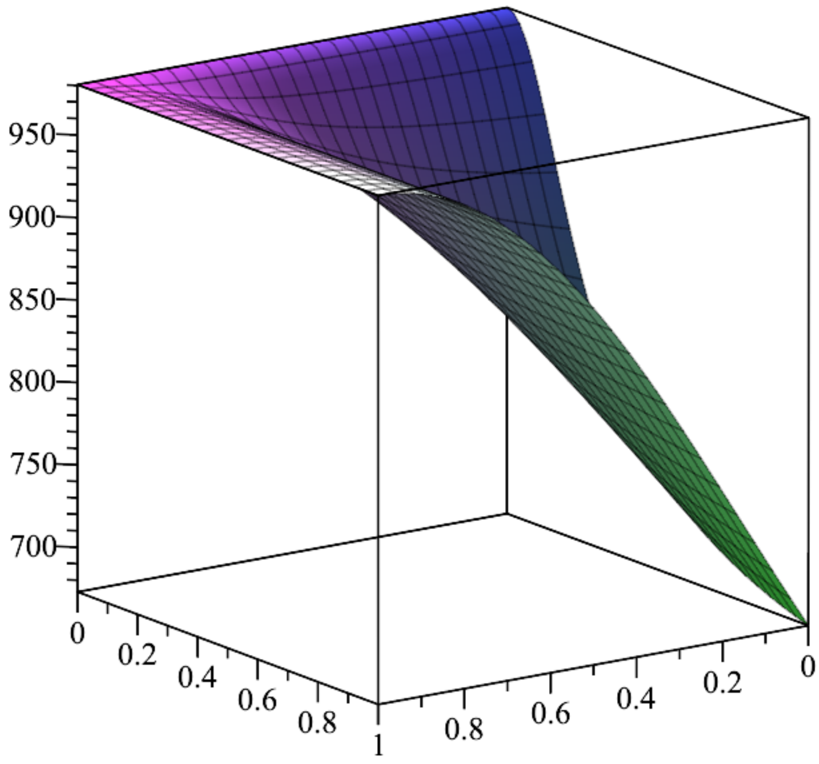

950
900
850
800
750
700
0
0.2
0.4
0.6
0.8
1
0.8
0.6
0.4
0.2
0

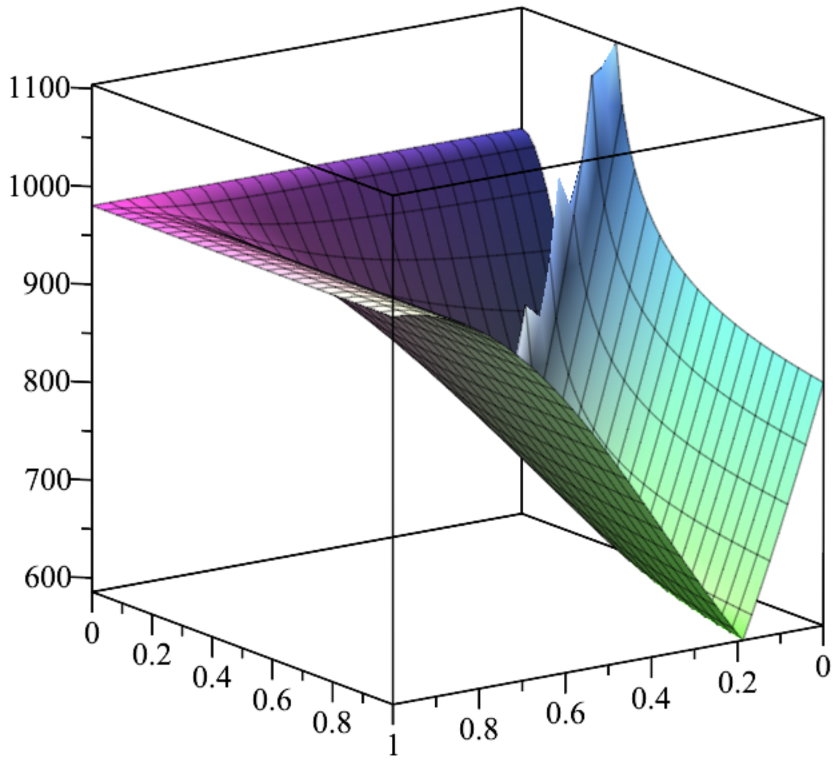

1100
1000
900
800
700
600
0
0.2
0.4
0.6
0.8
1
0.8
0.6
0.4
0.2
0

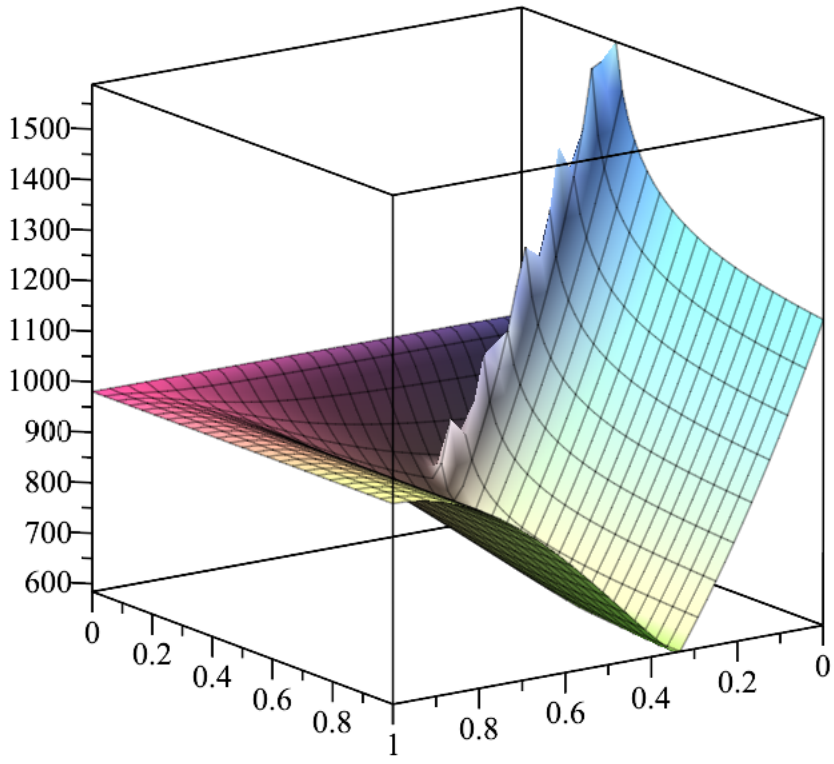

1500
1400
1300
1200
1100
1000
900
800
700
600
0
0.2
0.4
0.6
0.8
1
0.8
0.6
0.4
0.2
0

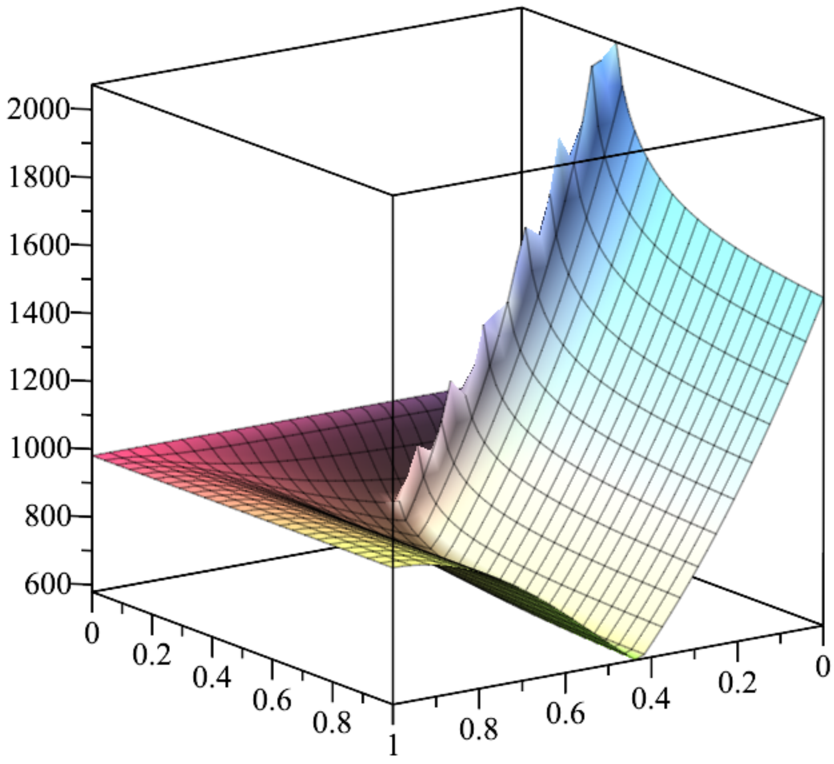

2000
1800
1600
1400
1200
1000
800
600
0
0.2
0.4
0.6
0.8
1
0.8
0.6
0.4
0.2
0

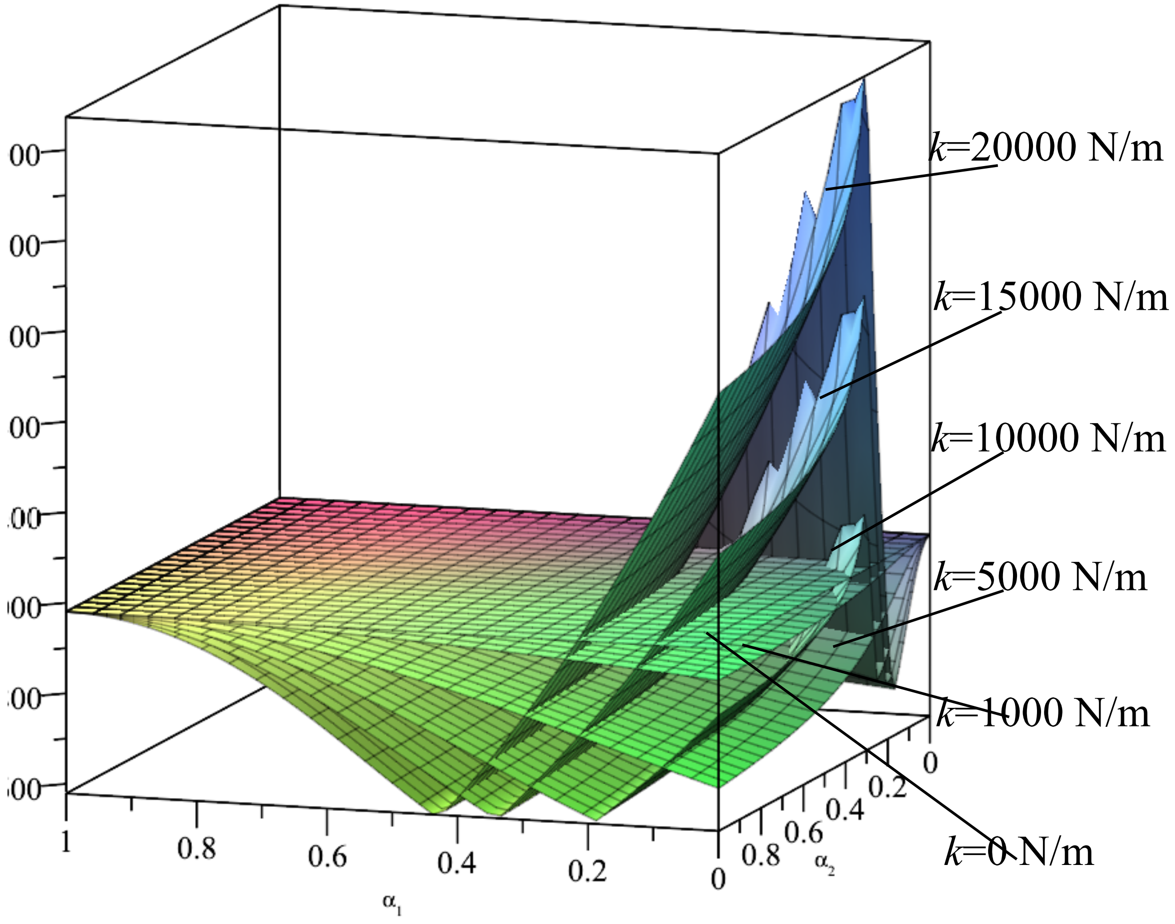
k=20000 N/m
k=15000 N/m
k=10000 N/m
k=5000 N/m
k=1000 N/m
k=0 N/m
1
0.8
0.6
0.4
0.2
0
α1
0.8
0.6
0.4
0.2
0
α2
